# Simulation Models for Exploring Magnetic Reconnection

**DOI:** 10.1007/s11214-025-01210-5

**Published:** 2025-09-09

**Authors:** Michael Shay, Subash Adhikari, Naoki Beesho, Joachim Birn, Jörg Büchner, Paul Cassak, Li-Jen Chen, Yuxi Chen, Giulia Cozzani, James Drake, Fan Guo, Michael Hesse, Neeraj Jain, Yann Pfau-Kempf, Yu Lin, Yi-Hsin Liu, Mitsuo Oka, Yuri Omelchenko, Minna Palmroth, Oreste Pezzi, Patricia H. Reiff, Marc Swisdak, Frank Toffoletto, Gabor Toth, Richard A. Wolf

**Affiliations:** 1 Bartol Research Institute, Department of Physics and Astronomy, University of Delaware, Newark, 19716 DE USA; 2 Department of Physics and Astronomy, West Virginia University, Morgantown, 26506 WV USA; 3 Department of Astronomy, University of Maryland, College Park, 20742 MD USA; 4 Center for Space Plasma Physics, Space Science Institute, Boulder, 80301 CO USA; 5 Max Planck Institute for Solar System Research, Göttingen, 27077 Germany; 6 NASA Goddard Space Flight Center, Greenbelt, 20771 MD USA; 7 Department of Physics, University of Helsinki, P.O. Box 68, 00014 Uusimaa Finland; 8 Department of Physics, University of Maryland, College Park, 20740 MD USA; 9 Institute for Research in Electronics and Applied Physics, University of Maryland, College Park, 20740 MD USA; 10 Los Alamos National Laboratory, Los Alamos, 87545 NM USA; 11 NASA Ames Research Center, Moffett Field, 94035 CA USA; 12 Center for Astronomy and Astrophysics, Technical University Berlin, Berlin, 10623 Germany; 13 Physics Department, Auburn University, Auburn, 36832 AL USA; 14 Department of Physics and Astronomy, Dartmouth College, Hanover, 03750 NH USA; 15 Space Sciences Laboratory, University of California, Berkeley, 94720 CA USA; 16 Trinum Research Inc., San Diego, 92126 CA USA; 17 Istituto per la Scienza e Tecnologia dei Plasmi (ISTP), Consiglio Nazionale delle Ricerche, Bari, I-70126 Italy; 18 Department of Physics and Astronomy, Rice University, Houston, 77005 TX USA; 19 University of Michigan, Ann Arbor, 48109 MI USA; 20 LPC2E, OSUC, Univ Orleans, CNRS, CNES, Orleans, F-45071 France; 21 Now at Advanced Computing Facility, CSC - IT Center for Science, Espoo, 02101 Uusimaa Finland

**Keywords:** Plasma simulation, Magnetic reconnection, Plasma physics, Magnetosphere, Solar corona, Turbulence, Numerical methods

## Abstract

Simulations have played a critical role in the advancement of our knowledge of magnetic reconnection. However, due to the inherently multiscale nature of reconnection, it is impossible to simulate all physics at all scales. For this reason, a wide range of simulation methods have been crafted to study particular aspects and consequences of magnetic reconnection. This article reviews many of these methods, laying out critical assumptions, numerical techniques, and giving examples of scientific results. Plasma models described include magnetohydrodynamics (MHD), Hall MHD, Hybrid, kinetic particle-in-cell (PIC), kinetic Vlasov, Fluid models with embedded PIC, Fluid models with direct feedback from energetic populations, and the Rice Convection Model (RCM).

## Introduction

Numerical computation has always played an important role in science. The term “computer” was used during the Renaissance to describe a person who performed mathematical calculations, and such computers were used extensively to calculate the positions of the planets. However, with the advent of digital computers last century, the role of such computation has exploded and revolutionized science in general. The study of magnetic reconnection has seen such a revolution in the last several decades as both numerical power has increased and numerical techniques have become more sophisticated.

Magnetic reconnection is considered a multiscale process because it allows physics that emerges at very small length and time scales to have global consequences in the system. A straightforward example of this large separation of scales is magnetic reconnection on the sun side of Earth’s magnetosphere. In this region, magnetic field lines are finally broken on a length scale of the order of $5\,\mathrm{km}$ which is the electron inertial length $d_{e} \equiv c/\omega _{pe}$. However, the dynamical effects of this breaking of field lines include driving global convection of the magnetosphere, a system spanning 100s of Earth radii ($R_{E}$) which is hundreds of thousands of $d_{e}$. A grid scale of about a $d_{e}$ over 100 Earth Radii requires about 100,000 spatial grid points in only 1 dimension. Clearly, accurately resolving the physics breaking the frozen-in constraint while simulating global scales is impossible.

The impossibility of globally simulating the whole 3D system and resolving all scales has led to the generation of a wide range of simulation models, each of which has its own strengths and weaknesses. Through many decades of research, scientists have carefully crafted these models for the particular application or applications they are studying. Typically, the more realistic physics that is included in the simulation, the more computationally expensive it is. Studies of the basic physics of magnetic reconnection (Biskamp 1996) have very often used kinetic PIC simulations which include all relevant physics, but require a simplified geometry and boundary conditions. Global magnetospheric simulations include the complex boundaries associated with the solar wind and the ionosphere, but until recently were required to be fluid models due to the cost of including kinetic effects.

In this paper we will provide an overview of the primary simulation models that are currently being used to study magnetic reconnection. Please note, however, that the topic of plasma simulation is extremely complex and detailed and cannot be fully covered in a single book, much less a single article. To assist the reader, we have included a table of information for representative simulations codes associated with the types of models described in this article (See Table 1 at the end of this section). If the reader wishes to dive even deeper into a particular model, there are many references available, many of which are cited in the individual sections of this paper and in the table. There are also excellent books devoted to the subject (e.g., Büchner et al. 2003; Büchner 2023).

**Table 1:** List of representative simulation codes discussed in this manuscript

Code	Language	Parallelization	Reference	Public
	URL			
Regional MHD	Fortran 77	None	Birn et al. (2025)	No
	https://doi.org/10.1029/2024JA033648			
Global MHD (BATSRUS)	Fortran 90	MPI, OpenMP, OpenACC	Tóth et al. (2012)	Yes
	https://github.com/SWMFsoftware/BATSRUS			
Test particle in MHD	Fortran 77	None	Birn et al. (2022)	No
	https://doi.org/10.3389/fspas.2022.908730			
Hall MHD/EMHD (F3D)	Fortran 90	MPI	Shay et al. (2004)	No
	https://doi.org/10.1063/1.1705650			
Hybrid PIC/EMHD (CHIEF)	Fortran 90, C++	MPI	Muñoz et al. (2018)	No
	-			
Global Hybrid PIC (HYPERS)	Fortran 77, C++	MPI	Omelchenko et al. (2021a)	Yes^1^
	https://ccmc.gsfc.nasa.gov/models/HYPERS-Global~2021/			
Kinetic PIC (P3D)	Fortran 90	MPI	Zeiler et al. (2002)	Yes
	https://github.com/spudam/P3D-PLASMA-PIC			
Kinetic PIC (VPIC)	C++	MPI / OpenMP / Kokkos	Bowers et al. (2008), Bird et al. (2021)	Yes
	https://github.com/lanl/vpic / https://github.com/lanl/vpic-kokkos			
Kinetic PIC (ExPIC)	Fortran 77	MPI	Bessho et al. (2019)	No
	https://doi.org/10.1029/2019GL083397			
Embedded Kinetic PIC (FLEKS)	C++	MPI	Chen et al. (2023)	Yes
	https://github.com/SWMFsoftware/FLEKS			
Kglobal	Fortran 90	MPI	Drake et al. (2019)	No
	-			
Hybrid Vlasov (HVM)	Fortran 90, Fortran 77	MPI, OpenMP, OpenACC, CUDA	Valentini et al. (2007)	No
	https://doi.org/10.1016/j.jcp.2007.01.001			
Global Hybrid Vlasov (Vlasiator)	C++	MPI, OpenMP	Palmroth et al. (2018a), Ganse et al. (2023)	Yes
	https://github.com/fmihpc/vlasiator			
Inner Magnetosphere (RCM)	Fortran 95	MPI	Toffoletto et al. (2003)	Yes^2^
	-			

^1^Available at NASA CCMC

^2^By request

In the field of magnetic reconnection research, more than one system of units is used. As of this writing, one can generally say that scientists specializing in theory/simulation primarily use cgs units and scientist specializing in observational analysis use SI units. We have chosen as much as possible to use cgs units in this paper, although the section on the Rice Convection Model has been left in SI units. For an excellent description on how to convert units between cgs and SI, please see the *NRL Plasma Formulary* (Huba 1998).

In magnetic reconnection the diffusion region occurs in thin boundary layers where the physics changes, ultimately allowing magnetic topology to change (Liu et al. 2025a). Although not exhaustive, the new physics which emerges in the diffusion region can be characterized by examining Ohm’s law, which comes from the fluid electron momentum equation.

1 $$ {\mathbf{E}} = -\frac{{\mathbf{u}} \times {\mathbf{B}}}{c} + \eta {\mathbf{J}} + \frac{\mathbf{J}\times \mathbf{B}}{n_{e} ec} + \frac{m_{e}}{e^{2}} \frac{\partial}{\partial t}\left ( \frac{\mathbf{J}}{n_{e}}\right ) - \frac{m_{e}}{e^{2}}\left (\frac{\mathbf{J}}{n_{e} e} \right )\cdot \nabla \left (\frac{\mathbf{J}}{n_{e}} \right ) -\frac{1}{n_{e} e}\nabla \cdot { \mathbf{p}}_{e}, $$

where ${\mathbf{E}}$ and ${\mathbf{B}}$ are the electric and magnetic fields, $\mathbf{J}$ is the current density, ${\mathbf{u}}$ is the single fluid bulk flow velocity, $\eta $ is the resistivity, ${\mathbf{p}}_{e}$ is the electron pressure tensor, $n_{e}$ is the number density, $e$ is the proton charge, $m_{e}$ is the electron mass and $c$ is the speed of light. The first term on the right hand side of Eq. (1) is the ideal term, the second term is the resistive term, the third term is the hall term, the fourth and fifth terms collectively represent electron inertia and the final term is due to the electron pressure tensor.

For the organization of the paper, we choose to move generally from fluid models to kinetic models as exemplified by terms on the right hand side of Eq. (1). we start with magnetohydrodynamics (MHD) and gradually increase in physical complexity until reaching fully kinetic simulations. We end with the Rice Convection Model (RCM), a widely used model for the inner magnetosphere, which acts as an inner boundary for magnetic field lines which are reconnecting in the magnetosphere. Section 2 describes MHD – first two terms. Section 3 describes Hall MHD including electron inertia – third and fourth terms. Section 4 describes Hybrid Simulations – generally also third and fourth terms. Section 5 describes kinetic particle-in-cell simulations – the physics of the fifth term is added, although it arises from the collective effects of many individual electrons. Section 6 describes embedding PIC codes into fluid models like MHD. Section 7 describes Kglobal, an MHD model which self consistently evolves energetic particles. Section 8 describes kinetic Vlasov models. And Section 9 describes the Rice Convection Model.

## MHD

### Equations of MHD

Magnetohydrodynamics (MHD) is the simplest fluid model used to study large scale plasma dynamics. MHD is based on the assumption that the characteristic length and time scales of the system under study are much larger than the length and time scales of the plasma species, usually Debye length ($\lambda _{D}$) or gyroradius and gyroperiod. Therefore, MHD represents the slow evolution of plasmas, often electrons and ions as a single fluid. The macroscopic behavior of the fluid in presence of a magnetic field is described by MHD using hydrodynamics and Maxwell’s equations.

Let us consider a fluid (in this case a plasma with ions and electrons), moving with a flow velocity $\mathbf{u}$, characterized by a mass density $\rho = m_{i} n_{i}$ where $m_{i}$ is the mass of protons ($m_{i}\gg m_{e}$) and $n_{i}$ is the number density of protons (with quasi-neutrality $n_{i}=n_{e}=n$), thermal pressure $p$, and a magnetic field $\mathbf{B}$. The evolution of these fields in space and time are governed by the MHD equations given by

2 $$ \frac{\partial \rho}{\partial t} = -\nabla \cdot (\rho \mathbf{u}), $$

3 $$ \rho \frac{\partial \mathbf{u}}{\partial t} + \rho (\mathbf{u}\cdot \nabla ) \mathbf{u} = -\nabla p + \frac{1}{4\pi}(\nabla \times \mathbf{B}) \times \mathbf{B} + \nu \nabla ^{2} \mathbf{u}, $$

4 $$ \frac{d}{dt}\left (\frac{p}{\rho ^{\gamma}}\right ) = 0, $$

5 $$ \frac{\partial \mathbf{B}}{\partial t} = \nabla \times (\mathbf{u} \times \mathbf{B}) + \frac{\eta c^{2}}{4\pi} \nabla ^{2} \mathbf{B}, $$

where $\gamma $ is the adiabatic index (usually $5/3$), $\nu $ is the dynamic viscosity, $\eta $ is the resistivity and $\eta c^{2}/4\pi $ collectively is known as the magnetic diffusivity. Here Eq. (2) is the continuity equation representing conservation of mass density, Eq. (3) is the momentum conservation equation, Eq. (4) is the simple adiabatic gas equation representing the conservation of energy and Eq. (5) is the induction equation, where the first term on the right is the advection term and the second one represents diffusion. Note, as a result of MHD approximations, the displacement current term is omitted in the induction equation. In an ideal situation, there are no dissipative processes and therefore $\nu $ and $\eta =0$ gives ideal MHD equations. Studies have shown that ideal MHD description is a very good approximation to study dynamical properties of strongly magnetized plasmas.

### Regional MHD Simulations

Several large-scale MHD approaches do not model the entire magnetosphere but only sections of it, such as certain magnetopause regions (dealt with elsewhere in this volume) or the magnetotail. Here we focus particularly on the magnetotail. The basic numerical approach used in regional MHD simulations is essentially similar to that used in (some) global simulations. It is typically based on explicit finite difference methods to solve the MHD equations. Minor differences might exist in adding resistive terms, which are usually necessary in local MHD to initiate reconnection. On the other hand, many global models use finite volume approaches in part due to the complex geometries involved (e.g., Powell et al. 1999).

The main difference, however, consists of the setup or initialization. Whereas global simulations typically involve of period of interaction with the solar wind to create a realistic magnetotail, local MHD simulations generally start from some equilibrium or near-equilibrium that models the stretched magnetotail (e.g., Schindler 1972). This approach provides more flexibility in treating different scenarios, for instance, varying the tail flaring between $y$ and $z$ (Birn and Hesse 2000) or including a local $B_{z}$ hump (Merkin and Sitnov 2016; Birn et al. 2018). This flexibility has also proven useful in PIC simulations that go beyond the commonly used initial 1D Harris sheet, most notably in addressing the holy grail of reconnection onset (e.g., Liu et al. 2014; see also Liu et al. 2025a).

On the other side, interactions with the ionosphere or the solar wind are incorporated only in some ad hoc fashion, if at all. Regional magnetotail MHD simulations therefore have been most successful in treating dynamic tail phenomena on relatively short time scales that are typically substorm related. The successes include

1. The demonstration that X-line formation and plasmoid ejection can be part of a 2D or 3D tearing-type instability of the tail (e.g., Birn and Hones 1981).
2. The demonstration that the build-up of the substorm current wedge (SCW), involving dipolarization and Region-1-type field-aligned currents (McPherron et al. 1973), can be due to the braking and azimuthal diversion of earthward flow from a near-tail reconnection site (Birn and Hesse 1991; Scholer and Otto 1991). This basic picture has been modified more recently, most notably by the addition of a Region-2 current system connecting to the ionosphere at lower latitude (Birn and Hesse 2014; Kepko et al. 2015), in agreement with observations (Sergeev et al. 2014). While the buildup of the SCW in the simulations is based on the shear and vorticity of the earthward flow, the persistence of the currents relies on the changes of the magnetic flux and pressure patterns brought about by the severance of a plasmoid and the resulting los of entropy and redistribution of the pressure. It is noteworthy that these features can be, and have been, found also in global simulations. In the regional simulations, however, they arise as consequences of an instability without involvement of external driving or feedback from the ionosphere. This would be harder to extract from the global simulations.
3. Regional MHD simulations have also been used to address the evolution prior to the onset of reconnection in the tail, demonstrating, specifically, the formation of a thin concentrated current sheet embedded in the near-tail plasma sheet. These approaches have included interaction with the solar wind in two complementary, ad-hoc ways. In one approach magnetic flux is added to the tail lobes (Birn and Schindler 2002), the other is based on low-latitude magnetic flux reduction from convection around the Earth toward the dayside (Hsieh and Otto 2014, 2015). Both mechanisms are expected from solar wind interaction. For more details, see the review by Sitnov et al. (2019).
4. Recently, regional MHD simulations have also demonstrated that the magnetotail may become unstable even under ideal 2D MHD constraints, when it includes a region of inverse (i.e. tailward) gradient of the normal magnetic field $B_{z}$ (denoted ‘$B_{z}$ hump’ instability; (Merkin and Sitnov 2016; Birn et al. 2018)).

### Global MHD

Global MHD models representing the (outer) magnetosphere of Earth typically extend around $100-200\,R_{E}$ in the flank and tail directions, and around $30\,R_{E}$ (beyond the bow shock) towards the Sun, where the solar wind is coming from. When, occasionally, the solar wind becomes sub-Alfvenic, the bow shock disappears and Alfvén wings form. In this case the upstream boundary has to be moved much further to minimize the boundary effects. It is computationally very demanding to obtain an accurate solution in such a large domain while resolving various structures such as the current sheets at the dayside magnetopause and in the tail. There are various approaches to overcome this difficulty, including the block-adaptive grid of the BATSRUS code (Powell et al. 1999; Tóth et al. 2012), the stretched Cartesian grid of OpenGGCM (Raeder et al. 1997) or the stretched spherical grid of the Lyon-Fedder-Mobarry (LFM) code (Lyon et al. 2004).

A general issue associated with all MHD simulations is preserving $\nabla \cdot \mathbf{B} = 0$ (e.g., Tóth et al. 2012). This issue also creates issues associated with the upstream boundary condition. Typically, we have observations at a single point near L1, a Lagrange point in between the Sun and the Earth, and assume that the solar wind and interplanetary magnetic field (IMF) have no variation in the transverse direction. If $B_{x}$ varies, and it certainly does, these assumptions lead to a finite $\nabla \cdot \mathbf {B} = \partial B_{x}/\partial x$ propagating into the domain. There are various approaches to handle this situation. One is to ignore the problem and propagate the finite $\nabla \cdot \mathbf {B}$ with the flow using some variation of the 8-wave scheme (Powell 1994). Another common approach is to set $B_{x}$ to a constant value, for example 0. Finally, one can relax the condition that the transverse gradients in the $y$ and $z$ directions are zero, and guess those gradients from the temporal evolution of $B_{x}$. Unfortunately this is an underspecified problem, so there is no unique solution. A typical approach is to smooth $B_{x}$ in time, and apply some minimum variance constraint. While theoretically nice, in practice the minimum variance approach does not work great. The likely reason is that the magnetic field is turbulent, so local changes in $B_{x}$ are not representative of the large scale tilt of the propagation plain.

The inner boundary conditions are usually applied at a sphere of radius 1.5 $R_{E}$ to 3 $R_{E}$ surrounding the Earth. One can use a semi-empirical electrodynamic solver (Ridley et al. 2004), or a fully empirical model (Weimer 1996, 2001) to calculate the $\mathbf {E}\times \mathbf {B}$ drift velocity at the inner boundary. Another important use of an electrodynamic solver is that it can also provide the $\mathbf {E}\times \mathbf {B}$ drift to an inner magnetosphere model (Wolf et al. 1982; Toffoletto et al. 2003; Buzulukova et al. 2010; Liemohn et al. 2001; Jordanova et al. 1994; Zaharia et al. 2006), which can calculate realistic ring current and associated pressure (and density) in the closed field line region during geomagnetic storms (Liemohn et al. 2018). The global MHD model can then relax its pressure (and density) towards the values supplied by the inner magnetosphere model. In return, the global MHD model can supply the plasma boundary conditions for the inner magnetosphere model at the edge of the closed field region as well as the magnetic field configuration (De Zeeuw et al. 2004; Meng et al. 2013).

Global models can properly represent the overall dynamics of the interaction of the solar wind with the magnetosphere, including the formation of the bow shock and the magnetopause, as well as the main current sheet in the magnetotail. Magnetic reconnection will happen on the dayside magnetopause and in the magnetotail in agreement with the theory of the Dungey cycle (Dungey 1961). The magnetic reconnection in the MHD simulation is not represented by the actual kinetic physics but it is approximated by numerical diffusion or artificial resistivity. Despite these caveats, global MHD models generate reconnection sites where the magnetic field changes sign and the reconnection rate is approximately correct (see Appendix A of Wang et al. (2022a)).

When the grid is relatively coarse, the numerical diffusion will easily adjust to reconnect the incoming magnetic flux carried by the solar wind. For a constant solar wind and IMF driving the simulation will settle to a steady state solution. Using fine grids in combination with low dissipation numerical methods can lead to a more dynamic reconnection process in the model. On the dayside, simulations can produce Flux Transfer Events (Raeder 2006) and in the tail flux ropes can be produced even by ideal MHD simulations. If the flux ropes in the tail are triggered by sign changes of the IMF $B_{z}$, the MHD simulations can match observations very well. A more challenging problem is reproducing substorms and sawtooth events. MHD models cannot do this well (Haiducek et al. 2020), and one needs to add either ionospheric outflow to regulate the reconnection rate (Brambles et al. 2013; Zhang et al. 2020), or kinetic reconnection physics (Wang et al. 2022a) to produce the typical spatial and temporal scales of sawtooth oscillations.

### Test Particles in MHD Simulations

Test particle approaches consist of tracing charged particle orbits in electromagnetic fields that are either prescribed in some plausible fashion or obtained from a simulation that typically does not contain individual particle information, most commonly based on MHD. The approach bridges the gap between large-scale MHD and small-scale particle simulations. In contrast to the latter, it can treat together realistic 3D space and large evolution time scales and realistic electron mass. However, it is not self-consistent and relies on whether the MHD model or the postulated $\mathbf{E}$, $\mathbf{B}$ fields capture the main physics. But that may also be considered an advantage as it permits studying the effects of large-scale fields in isolation.

In the magnetospheric context ions are usually treated by integration of the full orbit

6 $$ \frac{D\mathbf{u}}{D t} = \frac{e}{m}\Bigg( \mathbf{E} + \frac{1}{\gamma c} \textbf{u} \times \textbf{B}\Bigg). $$

Here $\gamma $ is the relativistic factor, which may be more relevant for electrons, $\mathbf{u} = \gamma \mathbf{w}$, where $\mathbf{w}$ is the particle velocity, $c$ is the speed of light and $D/Dt = \partial /\partial t + \mathbf{w}\cdot \nabla $ denotes the derivative along the full orbit. This equation is typically evolved using a high order Runge-Kutta (Press et al. 1992) or the Boris method (Birdsall and Langdon 1991). Full integration of Equation (6) over extended orbits is not practical for electrons, as it is more time consuming and might accumulate too large errors. Also, the adiabatic drift approximation, based on conservation of the magnetic moment $\mu $, is valid over larger areas in the magnetosphere and can be adequate for identifying typical acceleration mechanisms (e.g., Delcourt and Sauvaud 1994; Li et al. 1998; Zaharia et al. 2000; Gabrielse et al. 2012).

However, when the full history of electron orbits is considered; this may involve encounters of the reconnection site and low magnetic field, or high curvature regions, where the conservation of $\mu $ breaks down, and full orbit integration is required. Consequently, several codes have been developed that involve a transition between full orbits, integrated by Eq. (6), and drift orbits (e.g., Birn et al. 2004; Schriver et al. 2005; Ashour-Abdalla et al. 2011; Sorathia et al. 2017). The drift is described by the guiding center drift velocity (e.g., Birn et al. 2004)

7 $$ \mathbf{v}_{d} = \mathbf{v}_{E} -\frac{\mu c}{\gamma e} \frac{\mathbf{B}\times \nabla B}{B^{2}}- \frac{\gamma m_{e} cv_{\parallel}}{e} \frac{\mathbf{B}}{B^{2}}\times \frac{d\mathbf{b}}{dt} -\frac{m_{e} c}{e}\frac{\mathbf{B}}{B^{2}} \times \frac{d(\gamma \mathbf{v}_{E})}{dt} $$

where $\mu $ is the (relativistic) magnetic moment, $\mathbf{v}_{E}=\mathbf{E}\times \mathbf{B}/B^{2}$ and $\mathbf{b}=\mathbf{B}/B$. In addition, the field-aligned velocity is advanced by

8 $$ \frac{du_{\parallel}}{dt} = -\frac{e}{m_{e}}E_{\parallel }- \frac{\mu}{\gamma m_{e}}\frac{\partial B}{\partial s} - \big( \mathbf{u}_{E}+\mathbf{u}_{\nabla B}\big)\cdot \frac{d\mathbf{b}}{\partial t} $$

where now $\mathbf{u} = \gamma \mathbf{v}$ and $\mathbf{v} = \mathbf{v}_{E}+\mathbf{v}_{d}+\mathbf{v}_{\parallel}$ describes the guiding center velocity, $\mathbf{v}_{\nabla B}$ is the grad B drift, given by the second term on the right side of Eq. (7), and $d/dt$ is the derivative along the guiding center path. The transition between full orbit and drift orbit is typically determined from an adiabaticity criterion that is based on the ratio between the local field line curvature radius and the gyro radius based on the local magnetic field strength (e.g., Buchner and Zelenyi 1989). On the switch from drift to full orbit a phase has to be generated, which is typically chosen randomly. Although this can alter individual orbits and make them not reversible, it was found to have no significant effect on general conclusions about sources and properties of distributions (e.g., Birn et al. 2004).

Two different techniques are used, tracing particle motion either forward or backward in time. Forward tracing requires larger numbers of particles, sometimes comparable to those in PIC simulations, to obtain sufficient numbers at the points of interest (e.g., Scholer and Jamitzky 1987; Sachsenweger et al. 1989; Peroomian and El-Alaoui 2008; Ukhorskiy et al. 2017, 2018). However, since the particles are not interacting this approach is even more suitable for parallel processing than full particle simulations. In principle, this approach can also include wave scattering and collisions (albeit in an ad-hoc non self-consistent manner) to add to the simple collisionless advance.

Backward tracing is generally based on Liouville’s theorem of the conservation of phase space density $f$ to map $f$ from source locations to the final location of interest (e.g., Curran and Goertz 1989; Birn and Hesse 1994; Birn et al. 2004). It requires fewer orbits to identify properties at selected final locations, but relies on the validity of Liouville’s theorem, i.e. the absence of collisions. Backward tracing permits an easier identification of different sources contributing to the final population. Thus, sometimes a combination of both techniques is employed (e.g., Ashour-Abdalla et al. 2011).

Further complications are related to the use of MHD simulation results. Since the fields are given only on a finite grid, they have to be interpolated in space and time.

1. The advance of the drift equations (7) and (8) requires a third order spatial interpolation in B for continuous transition between grid cells, which could lead to spurious maxima or minima. This can be avoided, however, by employing a monotonicity algorithm (Hyman 1983; Birn et al. 2004).
2. Simple interpolation of the electric field could also yield spurious parallel components. This can be avoided, however, by various techniques, for instance, by interpolating $E_{\parallel}$ and $E_{\perp}$ separately (e.g., Birn et al. 2022).

## Hall MHD

### Introduction

The ideal-MHD model, as discussed in Sect. 2.1, is well-suited for magnetized plasmas when the dynamics is slow compared to the gyration time of charged particles around the magnetic fields and the length scales over which quantities vary is much larger than the gyroradius of the charged particles. However, going back many years in the study of neutral fluids, fluid models can lead to incorrect and paradoxical results at boundaries layers [e.g., d’Alembert’s paradox (Sect. 4.7 of Choudhuri 1998)]. In a magnetized plasma, these problems can occur where plasmas of two different origins abut against each other (such as at Earth’s magnetopause), at shocks and discontinuities such as Earth’s bow shock, and at localized regions where the magnetic field goes to zero, such as in the solar corona near sunspots.

Magnetic reconnection, in particular, occurs at a boundary layer at a region where at least two components of the magnetic field go to zero, so it is a key example of a physical process that cannot be faithfully modeled by ideal-MHD. Often in numerical simulations, ideal-MHD is used anyway, with numerical dissipation allowing reconnection to occur with the hope that it mimics the actual process. Another approach employs resistivity to model the effect of collisions; this is a useful approach in systems for which collisions are dynamically relevant, but many settings where reconnection occurs – especially in space and the solar corona – are weakly collisional or effectively collisionless (Priest and Forbes 2000; Cassak and Shay 2012). There are examples where either approach can be good enough for the questions being asked. For other questions that rely on a faithful representation of the physics in the regions where ideal- and resistive-MHD break down, a new model is necessary. In this section, we discuss a number of approaches within the fluid description that are used to go beyond ideal- and resistive-MHD simulations. In later sections, simulation techniques using the kinetic theory of gases are discussed. There are previous review papers discussing Hall-MHD and numerical approaches (Vasyliunas 1975; Huba 1995, 2003; Gómez 2006).

### The Hall-MHD Model

The equations of Hall-MHD are similar to those of MHD with one key difference. In resistive-MHD, Ohm’s law is given (in cgs units) by

9 $$ {\mathbf{E}} + \frac{{\mathbf{u}} \times {\mathbf{B}}}{c} = \eta {\mathbf{J}}, $$

where ${\mathbf{E}}$ and ${\mathbf{B}}$ are the electric and magnetic fields, ${\mathbf{u}}$ is the single fluid bulk flow velocity, $\eta $ is the resistivity, and ${\mathbf{J}} = (c/4\pi ) \nabla \times {\mathbf{B}}$ is the current density; in ideal-MHD, $\eta $ is set to zero. To go beyond this model, we revisit where Ohm’s law comes from.

The equation of motion of an electron fluid (i.e., Newton’s 2nd law) in a fully ionized plasma (in cgs units) is (Braginskii 1965)

10 $$ m_{e} \frac{d{\mathbf{u}}_{e}}{dt} = -e \left ({\mathbf{E}} + \frac{{\mathbf{u}}_{e} \times {\mathbf{B}}}{c}\right ) - \frac{1}{n_{e}} \nabla \cdot {\mathbf{p}}_{e} + {\mathbf{R}}_{e}, $$

where $m_{e}$ is the electron mass, ${\mathbf{u}}_{e}$ is the electron bulk flow velocity, $-e$ is the electron charge, $n_{e}$ is the electron density, ${\mathbf{p}}_{e}$ is the electron pressure which we write more generally as a tensor for now, and ${\mathbf{R}}_{e}$ represents the rate of change of momentum resulting from collisions between electrons and other electrons or other charged or neutral particles in the plasma. There are rigorous ways to determine the role of collisions (e.g., Braginskii 1965) that we do not employ here. Instead, we use the often used simpler approach that assumes ${\mathbf{R}}_{e} = m_{e} \nu _{ei} ({\mathbf{u}}_{i} - {\mathbf{u}}_{e})$, where $\nu _{ei}$ is the electron-ion collision frequency, and ${\mathbf{u}}_{i}$ is the ion bulk flow velocity, and for simplicity we assume the plasma has only electrons and ions (it is fully ionized).

To recover the resistive-MHD Ohm’s law from this equation, first the “electron inertia term” $m_{e} d{\mathbf{u}}_{e} / dt$ and the “electron pressure gradient term” $-(\nabla \cdot {\mathbf{p}}_{e}) / n_{e}$ are ignored for reasons we return to in Sect. 3.6. Second, the single fluid bulk flow velocity used in MHD is ${\mathbf{u}} = (m_{i} n_{i} {\mathbf{u}}_{i} + m_{e} n_{e} {\mathbf{u}}_{e})/(m_{i} n_{i} + m_{e} n_{e})$ and the current density ${\mathbf{J}} = n_{i} q_{i} {\mathbf{u}}_{i} - n_{e} q_{e} {\mathbf{u}}_{e} \simeq n_{e} q_{e}({\mathbf{u}}_{i} - {\mathbf{u}}_{e})$, where the latter form uses the assumption of quasi-neutrality $n_{i} q_{i} - n_{e} q_{e} \simeq 0$ with $q_{i}$ and $q_{e}$ being the charge of ions and electrons respectively. Using these expressions to write ${\mathbf{u}}_{e}$ in terms of ${\mathbf{u}}$ and ${\mathbf{J}}$ gives ${\mathbf{u}}_{e} = {\mathbf{u}} - ({\mathbf{J}}/ne) [m_{i} n_{i} / (m_{i} n_{i} + m_{e} n_{e})] \simeq {\mathbf{u}} - ({\mathbf{J}}/ne)$, where in the latter form, we use the approximation that $m_{i} \gg m_{e}$, since it is at least 1836 in an electron-ion plasma. Using these approximations in Eq. (10) and dividing by $e$ gives

11 $$ {\mathbf{E}} + \frac{{\mathbf{u}} \times {\mathbf{B}}}{c} = \frac{{\mathbf{J}} \times {\mathbf{B}}}{n_{e} e c} + \eta {\mathbf{J}}, $$

where the resistivity $\eta $ is defined as $m_{e} \nu _{ei} / n_{e} e^{2}$. If one additionally ignores the ${\mathbf{J}} \times {\mathbf{B}} / n_{e} e c$ term, what remains is the resistive-MHD Ohm’s law in Eq. (9). If instead, one ignores the resistive term $\eta {\mathbf{J}}$, the result is

12 $$ {\mathbf{E}} + \frac{{\mathbf{u}} \times {\mathbf{B}}}{c} = \frac{{\mathbf{J}} \times {\mathbf{B}}}{n_{e} e c}. $$

The term on the right is called the “Hall electric field” ${\mathbf{E}}_{H}$ (or simply the “Hall term”), and Eq. (12) is called the “Hall-MHD Ohm’s law”. Simply coupling this equation to the rest of the ideal-MHD equations gives the Hall-MHD model:

13 $$\begin{aligned} \frac{\partial \rho}{\partial t} + \nabla \cdot (\rho {\mathbf{u}}) = & 0, \end{aligned}$$

14 $$\begin{aligned} \rho \left [\frac{\partial {\mathbf{u}}}{\partial t} + ({\mathbf{u}} \cdot \nabla ) {\mathbf{u}} \right ] = & -\nabla p + \frac{{\mathbf{J}} \times {\mathbf{B}}}{c}, \end{aligned}$$

15 $$\begin{aligned} \frac{\partial p}{\partial t} + ({\mathbf{u}} \cdot \nabla ) p = & - \gamma p (\nabla \cdot {\mathbf{u}}) \end{aligned}$$

16 $$\begin{aligned} \frac{\partial {\mathbf{B}}}{\partial t} = & -c \nabla \times {\mathbf{E}}, \end{aligned}$$

17 $$\begin{aligned} {\mathbf{E}} + \frac{{\mathbf{u}} \times {\mathbf{B}}}{c} = & \frac{{\mathbf{J}} \times {\mathbf{B}}}{n_{e} e c} \end{aligned}$$

18 $$\begin{aligned} \nabla \times {\mathbf{B}} = & \frac{4\pi {\mathbf{J}}}{c} \end{aligned}$$

with the auxiliary equation $\nabla \cdot {\mathbf{B}} = 0$, and where $\gamma $ is the single fluid ratio of specific heats, typically taken to be 5/3. Note $n_{e}$ in Eq. (17) is related to the MHD mass density via $\rho = m_{i} n_{i} + m_{e} n_{e}$, so $n_{e} = \rho / (m_{i} e / q_{i} + m_{e}) \simeq Z \rho / m_{i}$, where we again assume $m_{i} \gg m_{e}$, we use quasi-neutrality to write $q_{i} n_{i} \simeq e n_{e}$, and define $Z = q_{i} / e$ as the degree of ionization. With these assumptions, Eqs. (13) - (18) form a closed set of equations, and therefore can be used to model physical systems. Technically, these equations actually give the “ideal Hall-MHD model” since resistivity is not retained here.

It is important to note a confusing aspect of these equations. The Hall term is proportional to ${\mathbf{J}} \times {\mathbf{B}}$, and Eq. (14) also contains a term including ${\mathbf{J}} \times {\mathbf{B}}$. It is tempting to draw relations between the two terms because of this outward similarity, but this should not be done. The two terms have different dimensions: ${\mathbf{J}} \times {\mathbf{B}} / c$ is a force density and ${\mathbf{J}} \times {\mathbf{B}} / n_{e} e c$ is an electric field. They have completely different manifestations and impacts on the physics, and therefore actually are not related despite their similar forms.

### Hall-MHD Physics

The only difference between the ideal-MHD and Hall-MHD models is the Hall electric field, and here we investigate the physics introduced by this term. Let $\tilde{L}$ be a characteristic length scale over which the plasma properties vary, and let $\tilde{r}_{Li}$ be the characteristic (Larmor) radius of the ions as they gyrate around a magnetic field of characteristic strength $\tilde{B}$. The Hall electric field is small and can be neglected if $\tilde{L} \gg \tilde{r}_{Li}$, and doing so brings us back to ideal-MHD. It is important to retain the Hall electric field, and therefore use Hall-MHD, when $\tilde{L} \lesssim \tilde{r}_{Li}$.

**Fig. 1:**
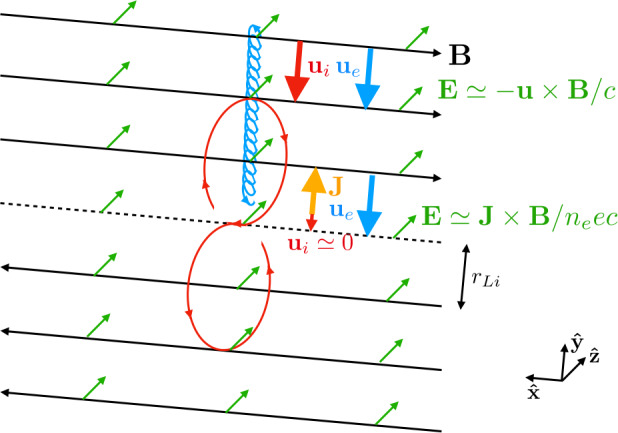
**Physics of the Hall effect in reversing magnetic fields.** A reversing anti-parallel magnetic field in the $\pm {\mathbf{\hat{x}}}$ direction is drawn using black arrows, with the neutral line as the dashed line. A uniform electric field in the ${\mathbf{\hat{z}}}$ direction is drawn in green. The smaller blue trajectory is that of an electron $E \times B$ drifting towards the neutral line with bulk velocity ${\mathbf{u}}_{e}$. An ion $E \times B$ drifting far from the neutral line has bulk ion velocity ${\mathbf{u}}_{i}$ identical to ${\mathbf{u}}_{e}$, so the electric field in this region is given by ${\mathbf{E}} = -{\mathbf{u}} \times {\mathbf{B}} / c$, where ${\mathbf{u}}$ is the single fluid (MHD) bulk flow velocity. Within an ion gyroradius $r_{Li}$ of the neutral line, the electron continues with bulk flow ${\mathbf{u}}_{e}$, while the ion demagnetizes (in the red trajectory) and ${\mathbf{u}}_{i}$ becomes small. In this region, there is a non-zero current density ${\mathbf{J}}$ (orange), and the electric field in this region is predominantly given by the Hall electric field ${\mathbf{E}} = {\mathbf{J}} \times {\mathbf{B}} / n_{e} e c$

To see why structure below the ion gyroscale gives rise to the Hall effect, consider a magnetic field ${\mathbf{B}}$ that reverses direction over a scale $\tilde{r}_{Li}$ or less, but on larger scales than the characteristic electron gyroradius $\tilde{r}_{Le}$. For specificity, consider a magnetic field pointing in the $\pm {\mathbf{\hat{x}}}$ direction, as sketched as the black arrows in Fig. 1. The neutral line, where the magnetic field strength vanishes, is the dashed black line. Suppose also there is a uniform electric field ${\mathbf{E}}$ pointing everywhere in the ${\mathbf{\hat{z}}}$ direction, as sketched as the green arrows. (We use a reversed magnetic field and uniform electric field for illustrative purposes due to its relation to the reconnection process, but the Hall effect is important for any magnetic field configuration that varies on $\tilde{r}_{Li}$ scales.)

At distances from the magnetic field reversal that greatly exceed $\tilde{r}_{Li}$, ions and electrons undergo the $E \times B$ drift that gives rise to a bulk flow towards the neutral line; the bulk flow velocity of ions and electrons, ${\mathbf{u}}_{i}$ and ${\mathbf{u}}_{e}$, respectively, are identical. The $E \times B$ drift of the electrons above the neutral line is sketched as the blue curve. In the region farther from the neutral line than $\tilde{r}_{Li}$, the electric field is given by ${\mathbf{E}} = -{\mathbf{u}} \times {\mathbf{B}} / c$, where ${\mathbf{u}} = {\mathbf{u}}_{i} = {\mathbf{u}}_{e}$ is the MHD bulk flow velocity.

As the ions reach a distance from the neutral line that is equal to its gyroradius, the ions cross the neutral line and are immersed in a magnetic field pointing in the opposite direction. Their gyromotion changes direction, and they make figure 8 orbits, sketched as the red curve. (They also accelerate in the ${\mathbf{\hat{z}}}$ direction due to the electric field, but this is omitted from the sketch.) Consequently, their bulk velocity becomes small, ${\mathbf{u}}_{i} \simeq 0$.

Since we assumed $\tilde{L} > \tilde{r}_{Le}$, the electrons have a smaller gyroradius and do not see the magnetic field reversal, so they continue to undergo the $E \times B$ drift towards the neutral line. The key is that the ions and electrons are undergoing different dynamics between distances from the neutral line of $\tilde{r}_{Li}$ and $\tilde{r}_{Le}$!

In this region, the difference in the bulk motion between ions and electrons implies there is a net current density ${\mathbf{J}}$, sketched as the orange arrow, and called the Hall current. The current density is perpendicular to the magnetic field ${\mathbf{B}}$. Then, between $\tilde{r}_{Li}$ and $\tilde{r}_{Le}$ from the neutral line, the electric field is given by the Hall electric field ${\mathbf{E}}_{H} = {\mathbf{J}} \times {\mathbf{B}} / n_{e} e c$. This exemplifies why the Hall electric field is important between ion and electron gyroscales.

This situation in a plasma is analogous to the Hall effect in condensed matter physics, where it was originally discovered by Edwin Hall (a graduate student) in 1879 (Hall et al. 1879). It has extensive applications in that field of study. The derivation generalizing shear Alfvén waves to include the Hall effect happened as early as 1954 by Jim Dungey (Dungey 1954), the same person who first understood magnetic reconnection and gave the process its name.

### Linear Waves in Ideal Hall-MHD

It would take us too far afield to elucidate how the Hall electric field modifies all the physics of ideal-MHD. Rather, we highlight one important example – linear waves. In ideal-MHD, there are three propagating linear waves available to a uniform plasma: the shear Alfvén wave and the fast and slow magnetosonic waves. The Hall electric field introduces two wave modes that become important between ion and electron gyroscales – the whistler wave and the kinetic Alfvén wave.

#### The Whistler Wave

First, we consider transverse waves propagating along a uniform background magnetic field of strength $B_{0}$, which without loss of generality we take to be in the $y$ direction, in a plasma of equilibrium mass density $\rho _{0} \simeq m_{i} n_{i}$ and with no equilibrium bulk flow or current ${\mathbf{u}}_{0} = 0$ and ${\mathbf{J}}_{0} = 0$. The wave vector ${\mathbf{k}}$ is also in the $y$ direction. Linearizing the Hall-MHD equations (13)-(18) about the given equilibrium with ${\mathbf{k}}$ parallel to ${\mathbf{B}}_{0}$ and solving for the dispersion relation gives

19 $$ \omega ^{2} = k^{2} v_{A0}^{2} \left ( 1 + \frac{k^{2} d_{i0}^{2}}{2} + \sqrt{k^{2} d_{i0}^{2} + \frac{k^{4} d_{i0}^{4}}{4}}\right ), $$

where $\omega $ is the wave frequency, $v_{A0} = B_{0} / (4 \pi \rho _{0})^{1/2}$ is the Alfvén speed, and $d_{i0} = (m_{i} c^{2} / 4 \pi n_{0} q_{i}^{2})^{1/2}$ is the ion inertial length.

In the limit of $k d_{i0} \rightarrow 0$, this dispersion relation reduces to $\omega ^{2} \rightarrow k^{2} v_{A0}^{2}$, which is simply the shear Alfvén wave from ideal-MHD. In the other extreme, consider the limit $k d_{i0} \rightarrow \infty $. The 1 in Eq. (19) becomes negligible compared to $k^{2} d_{i0}^{2}/2$ outside the square root, and the $k^{2} d_{i0}^{2}$ is negligible compared to $k^{4} d_{i0}^{4}/4$ inside the square root, so to low order the dispersion relation becomes

20 $$ \omega ^{2} \rightarrow k^{4} v_{A0}^{2} d_{i0}^{2} \hspace{1cm} ({\mathrm{as}} \ k d_{i0} \rightarrow \infty ). $$

This is the dispersion relation for the so-called “whistler wave.”

**Fig. 2:**
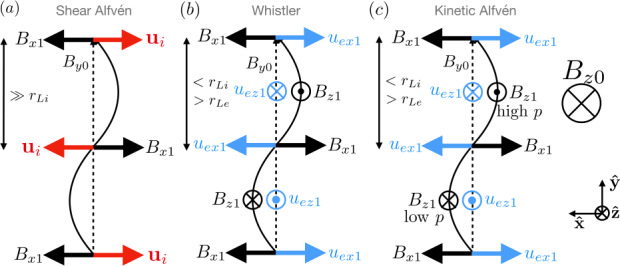
**Sketch of how the Hall effect impacts linear waves in MHD.** The wave structure for (a) shear Alfvén waves, (b) parallel propagating whistler waves, and (c) kinetic Alfvén waves. The equilibrium magnetic field $B_{y0}$ is the dashed black arrow. The perturbed magnetic field ${\mathbf{B}}_{1}$ are the black arrows. The perturbed ion bulk flow ${\mathbf{u}}_{i}$ are the red arrows, which occur in (a) because the wavelength is much larger than the ion gyroradius. In (b) and (c), the wavelength is at or below ion gyroscales, so the bulk flow is due to electron motion in the blue arrows. In (c), there is a large out-of-plane equilibrium magnetic field $B_{z0}$, so the magnetic perturbation $B_{z1}$ changes the magnetic pressure which to first order and requires a change to the gas pressure $p$

Physically, the whistler wave is the sub-ion gyroscale counterpart of the shear Alfvén wave. The plasma properties in a shear Alfvén wave are sketched in Fig. 2(a). The equilibrium magnetic field $B_{y0}$ is the dashed black arrow. A magnetic perturbation transverse to the equilibrium $B_{x1}$ is the large black arrow. Since the size of the wave is far larger than the ion gyroradius, the frozen-in ions and electrons feel a restoring force analogous to plucking a guitar string, generating bulk flows in the $x$ direction (shown for ions as the red arrows). The shear Alfvén wave is a linearly polarized wave.

The whistler wave, sketched in panel (b), occurs when the magnetic field varies between ion and electron gyroscales. Then, the ions are not frozen-in and do not respond to the plucked magnetic field line, but the electrons are frozen in and feel a restoring force. The $x$ component of the perturbed electron flow $u_{ex1}$ is sketched as the blue arrows. Since the electrons have a bulk velocity but the ions do not, this means there is a net current density, which induces a oscillating magnetic field out of the plane, labeled as $B_{z1}$. Similarly, the varying $B_{x1}$ requires a $u_{ez1}$ to sustain the current. This turns the wave into a circularly polarized wave. The polarization is right handed, *i.e.,* a receiver sees the magnetic field rotate in a counterclockwise direction.

There is a crucial difference between the shear Alfvén wave and the whistler wave. The sheer Alfvén wave has a phase speed $\omega / k = v_{A0}$, which is a constant independent of $\omega $ and $k$, so a shear Alfvén wave travels at the same speed regardless of its wavelength. It is an example of a non-dispersive wave that retains its waveform as it propagates (like a light wave in vacuum or a sound wave in a neutral fluid). In contrast, the phase speed for the whistler wave, from Eq. (20), is $\omega / k = k v_{A0} d_{i0}$. Thus, the phase speed is faster for shorter wavelength waves. This is an example of a dispersive wave, since a wave packet does not retain its shape. The dispersive nature is what gives the wave its name; it makes a characteristic “whistle” from high frequencies descending to low frequencies when it is detected at a location away from where it was generated. For example, when a lightning strike in one hemisphere excites a whistler wave on Earth’s magnetic field, the high frequencies arrive before the low frequencies in the other hemisphere.

This analysis reveals the critical scale at which the Hall effect becomes important for transverse bending of the magnetic field. The three terms in Eq. (19) proportional to $d_{i0}$ are absent if the Hall term is left out of the governing equations (*i.e.,* in ideal-MHD). By comparing the first term to the other terms, we see they become important when $k d_{i0}$ is on the order of 1. This means that the Hall term becomes important at length scales below $d_{i0}$.

The ion inertial scale is related to a form of the ion gyroradius. To see this, note that an ion moving at the Alfvén speed $v_{A0}$ that gyrates around a magnetic field of strength $B_{0}$ has a gyroradius $r_{Li}$ given by

21 $$ r_{Li} = \frac{v_{A0}}{\Omega _{ci0}} = d_{i0}, $$

where $\Omega _{ci0} = q_{i} B_{0} / m_{i} c$ is the ion gyrofrequency. Thus, the scale at which the Hall effect becomes important for transverse perturbations is when gradient scales are comparable to the ion inertial scale. Using characteristic scales for Earth’s dayside magnetosheath, $d_{i0} \simeq $ 70 km; for the solar corona, $d_{i0} \simeq $ 2 m. Thus, we have a feel for length scales over which we need to use Hall-MHD instead of ideal-MHD in two important space applications.

#### The Kinetic Alfvén Wave

Now, we consider the kinetic Alfvén wave. These waves are almost completely longitudinal, but not perfectly longitudinal. To allow for a strong analogy to the whistler wave, consider a uniform magnetic field that has a very large $z$ component $B_{z0} \gg 0$ and very small $y$ component $B_{y0}$, as sketched in Fig. 2(c). As in panels (a) and (b), the wave propagates in the $y$ direction. If $B_{z0}$ were zero, it would be the whistler wave. Physically, if the wavelength is between ion and electron gyroscales, this perturbation reacts similar to a whistler in that it sets up an electron flow that bends the magnetic field out of the plane. The perturbed magnetic field therefore has a component in the same or opposite direction as $B_{z0}$. Where $B_{z0}$ and the perturbed magnetic field are parallel, the magnetic pressure $(B_{z0} + B_{z1})^{2}/8\pi $ is greater than the initial pressure $B_{z0}^{2}/8\pi $ to first order in the perturbed field. (Note, for the whistler wave, the pressure difference $B_{z1}^{2}/8\pi $ is second order in the perturbed magnetic field, so it is negligible.) Similarly, where $B_{z1}$ opposes $B_{z0}$, the magnetic pressure decreases to first order. Now, if the plasma is overall low $\beta $ (since we took $B_{z0}$ to be large), this magnetic pressure imbalance cannot be maintained, so the gas pressure has to change in order to balance pressure. Electrons move along the magnetic field and ions move across the magnetic field to move from the high magnetic pressure region to the low magnetic pressure region, setting up a high and low gas pressure region as denoted in panel (c). This describes the physics of the kinetic Alfvén wave.

To find the dispersion relation of the kinetic Alfvén wave, we back up to the full dispersion relation of any wave in Hall-MHD. Linearizing Eqs. (13) - (18) around a uniform magnetic field, density, and pressure with an arbitrary linear perturbation and solving gives the following dispersion relation (Rogers et al. 2001):

22 $$\begin{aligned} \omega ^{6} & - (c_{ms}^{2} + v_{Ak}^{2} + k^{2} v_{Ak}^{2} d_{i0}^{2}) k^{2} \omega ^{4} + \\ & [c_{ms}^{2} + c_{s}^{2} (1 + k^{2} d_{i0}^{2})] k^{4} v_{Ak}^{2} \omega ^{2} - k^{6} v_{Ak}^{4} c_{s}^{2} = 0, \end{aligned}$$

where $v_{A}^{2} = B_{0}^{2}/(4\pi \rho _{0})$ is the total Alfvén speed, $c_{s}^{2} = \gamma p_{0} / \rho _{0}$ is the sound speed, $c_{ms}^{2} = c_{s}^{2} + v_{A}^{2}$ is the total fast magnetosonic speed, and $v_{Ak}^{2} = B_{y0}^{2} / 4 \pi \rho _{0}$ is the Alfvén speed based only on $B_{y0}$. In the $k^{2} d_{i0} \rightarrow 0$ long wavelength limit, this equation reduces to the dispersion relation for ideal-MHD waves. In the limit of large $k d_{i0}$, one solution is a high frequency solution which is approximately given by balancing the first two terms in Eq. (22), which gives $\omega ^{2} \simeq k^{4} v_{Ak}^{4} d_{i0}^{2}$, the whistler wave dispersion relation in Eq. (20). There is also a medium frequency solution which arises from balancing the middle two terms in Eq. (22). This ratio in general is

23 $$ \omega ^{2} \simeq \frac{c_{ms}^{2} + c_{s}^{2} (1 + k^{2} d_{i0}^{2})}{c_{ms}^{2} + v_{Ak}^{2} + k^{2} v_{Ak}^{2} d_{i0}^{2}} k^{2} v_{Ak}^{2}. $$

In the limit in which $c_{ms}^{2} \ll c_{s}^{2} k^{2} d_{i0}^{2}$ and $c_{ms}^{2} \gg v_{Ak}^{2} + k^{2} v_{Ak}^{2} d_{i0}^{2}$, the resulting dispersion relation is $\omega ^{2} \simeq (c_{s}^{2}/c_{ms}^{2}) k^{4} d_{i0}^{2} v_{Ak}^{2}$, which in the $v_{A}^{2} \gg c_{s}^{2}$ (low $\beta $) limit gives $\omega ^{2} \simeq (c_{s}^{2}/v_{A}^{2}) k^{4} d_{i0}^{2} v_{Ak}^{2}$. Since $k^{2} v_{Ak}^{2} = k_{\|}^{2} v_{A}^{2}$, this becomes

24 $$ \omega ^{2} \simeq k_{\|}^{2} k^{2} v_{A}^{2} \rho _{s}^{2}, $$

where $\rho _{s}^{2} = c_{s}^{2} / \Omega _{ci}^{2}$ is the ion Larmor radius based on the sound speed. This is the dispersion relation for the kinetic Alfvén wave. As with the whistler wave, the kinetic Alfvén wave is dispersive with $\omega / k \propto k_{\|}$, so it gets faster for smaller wavelengths. The length scale at which the Hall term becomes important for the kinetic Alfvén wave is $\rho _{s}$ (as opposed to $d_{i0}$ for the whistler wave).

### A Numerical Algorithm for Hall-MHD

Including the Hall electric field has a significant impact on numerical simulations relative to MHD simulations. One way to think of why this is the case is that the waves in ideal-MHD are non-dispersive, so waves at any scale from the large scale down to the computational grid scale travel at the same speed. In Hall-MHD, as discussed in the previous section, both whistler and kinetic Alfvén waves are dispersive, so waves at the grid scale (which needs to be sub-ion gyroscale to capture the Hall electric field) are considerably faster than waves at the large scale. This makes the Hall-MHD equations “stiff” – one must use a much smaller time step in Hall-MHD than in ideal-MHD, leading to a significant increase in the run time and expense of the simulation.

There are numerous algorithms that can be used to numerically evolve the equations in time. We provide one in detail, and mention references to other algorithms that have been used. We highlight the F3D code (Shay et al. 2004), which has been used for many years to study magnetic reconnection. First, the evolution equations are written in conservative form as

25 $$\begin{aligned} \frac{\partial n}{\partial t} + \nabla \cdot {\mathbf{J}}_{i} = & 0, \end{aligned}$$

26 $$\begin{aligned} \frac{\partial {\mathbf{J}}_{i}}{\partial t} + \nabla \cdot \left ( \frac{{\mathbf{J}}_{i} {\mathbf{J}}_{i}}{n} + \frac{p{\mathbf{I}}}{m_{i}} + \frac{{\mathbf{B}} {\mathbf{B}}}{4 \pi m_{i}} - \frac{B^{2} {\mathbf{I}}}{8\pi m_{i}} \right ) = & 0, \end{aligned}$$

27 $$\begin{aligned} \frac{\partial p}{\partial t} + \nabla \cdot ({\mathbf{u}} p) + (\gamma - 1) p (\nabla \cdot {\mathbf{u}}) = & 0, \end{aligned}$$

28 $$\begin{aligned} \frac{\partial {\mathbf{B}}}{\partial t} + c \nabla \times {\mathbf{E}} = & 0, \end{aligned}$$

with auxiliary equations ${\mathbf{E}} = {\mathbf{J}} \times {\mathbf{B}} / n e c - {\mathbf{J}}_{i} \times {\mathbf{B}} / n c$ and ${\mathbf{J}} = (c/4\pi )\nabla \times {\mathbf{B}}$. Here, $n \simeq \rho / m_{i}$ is the number density of ions (approximately equal to the number density of electrons due to quasi-neutrality) and ${\mathbf{J}}_{i} = n {\mathbf{u}}$ is the ion flux density.

In F3D, these equations are stepped forward using the trapezoidal leapfrog technique, a predictor-corrector method that is well-equipped to handle conservative equations (Zalesak 1979, 1981). Each of the above equations can be written as a conservative equation of the form (Guzdar et al. 1993)

29 $$ \frac{\partial \psi}{\partial t} + \nabla \cdot {\mathbf{{\mathcal{F}}}} - D \nabla ^{2} \psi + F(\xi ) = 0, $$

where $\psi $ is the plasma variable in question, ℱ is a suitably defined flux, $D$ is a second order diffusion coefficient which can be added to the equations to represent resistivity, viscosity, or a numerical dissipation to improve code stability, and $F(\xi )$ is a suitably defined sink/source term in terms of any other plasma variables $\xi $. (Equation (28) is not exactly in this form, but an analogous expression holds.)

To write the numerical algorithm, we use the standard notation where a superscript $n$ on a plasma variable refers to the time step in question, so the initial values are set at $n = 0$, the first time step is $n = 1$, and so on. To evolve $\psi ^{n}$ to $\psi ^{n+1}$ in a time step $\Delta t$, the trapezoidal leapfrog algorithm is

30 $$\begin{aligned} \psi ^{n+1/2} = & \frac{\psi ^{n-1} + \psi ^{n}}{2} + \Delta t \left [ - \nabla \cdot {\mathcal{F}}^{n} \right . \\ & + \left . D \nabla ^{2} \psi ^{n-1} - F(\xi ^{n}) \right ] \end{aligned}$$

31 $$\begin{aligned} \psi ^{n+1} = & \psi ^{n} \Delta t \left [ - \nabla \cdot {\mathcal{F}}^{n+1/2} \right . \\ & + \left . D \nabla ^{2} \psi ^{n} - F(\xi ^{n+1/2}) \right ]. \end{aligned}$$

The first equation uses the data at the $n$’th time step and the data at the previous $n-1$’st time step to “predict” $\psi $ half a time step in the future. Then, the flux and source terms are evaluated at this intermediate time step to evolve $\psi $ the next half time step to the desired step $n + 1$. To go from $n = 0$ to $n = 1$, data is needed at $n = -1$, which is simply taken to be the same as the data at $n = 0$. This algorithm is second order in the time step $\Delta t$, meaning that the error from the algorithm is approximately a coefficient times the square of the time step. This is an example of an “explicit” time stepping algorithm because the data at the future time step is found completely using known data at the current or previous time steps.

The above shows how the equation is stepped forward temporally, but one also needs to calculate spatial derivatives. The approach F3D uses to calculate spatial derivatives is with a finite difference technique, which means the spatial derivatives are simply approximated by the derivative over the size of a grid scale instead of over an infinitesimal distance. For example, one approximation for the partial derivative in the $x$ direction on a grid with grid scale $\Delta x$ is $\partial \psi _{j} / {\partial x} \simeq (\psi _{j+1} -\psi _{j-1})/2 \Delta x$, where the $j$ subscript refers to the index of the spatial cell for which the derivative is desired. This is a second-order scheme because the error relative to the exact derivative scales like $\Delta x^{2}$. The F3D code uses the approximation

32 $$ \frac{\partial \psi _{j}}{\partial x} \simeq \frac{2}{3\Delta x} ( \psi _{j+1} -\psi _{j-1}) - \frac{1}{12 \Delta x} (\psi _{j+2}-\psi _{j-2}), $$

which is fourth order (the error scales like $\Delta x^{4}$), and the cost for this higher order derivative is that it requires data from two adjacent cells on each side rather than one. Analogous expressions hold for spatial derivatives in the $y$ and $z$ directions. Second order derivatives are given by the associated fourth order accurate approximation $\partial ^{2} \psi _{j}/\partial x^{2} \simeq [-(2/3) (\psi _{j+1} - \psi _{j-1}) - (1/12)(\psi _{j+2}+\psi _{j-2})]/(\Delta x)^{2}$.

The F3D code is written in Fortran 90 and is parallelized using Message Passing Interface (MPI) for use on supercomputers. The computational domain is rectangular with a fixed, regular grid. It can be run in two or three dimensions. When in two dimensions, the vectors can have an out of plane component even though all quantities are invariant in the out-of-plane direction; this is often referred to in the literature as “2.5 dimensional”. The F3D code does not explicitly enforce that $\nabla \cdot {\mathbf{B}} = 0$, but it has been demonstrated that the value is small when the initial magnetic field is divergence free. Numerous aspects of the F3D code are on user-controlled switches that can turn terms or effects on or off, and the initial plasma variable profiles and values are controlled by the user. Other features of F3D that are used to go beyond the Hall-MHD model will be treated in Sect. 3.6.

To run the F3D code, the user chooses the simulation domain size and desired grid scale to resolve the relevant physics, which is typically at least 5 times smaller than the relevant ion gyroradius. As F3D is an explicit finite difference code, the time step $\Delta t$ can be no larger than allowed by the so-called Courant-Friedrichs-Lewy (CFL) condition (Press et al. 1992), which requires $\Delta t \leq \Delta x / v_{{\mathrm{fastest}}}$, where $v_{{\mathrm{fastest}}}$ is the fastest speed that can occur in the system. For Hall-MHD, the fastest speed is typically the whistler or kinetic Alfvèn wave speed at or near the grid scale, but can be the fast magnetosonic speed for some ambient plasma conditions. 2D reconnection simulations with F3D are typically performed with time step about 40% of the CFL condition.

Any employed diffusion coefficients then need to be chosen. Often, the resistivity is not used for Hall-MHD, but a fourth-order diffusion numerical dissipation of the form $-D_{4}(\partial ^{4} \psi /\partial x^{4}+\partial ^{4} \psi / \partial y^{4}+\partial ^{4} \psi /\partial z^{4})$ on the right hand side of Eq. (29) is included in F3D to damp structures at the grid scale while minimally affecting larger scale structures. In order to preserve the five point stencil used for the other finite differences, second order accuracy in the fourth order derivatives is employed, with $\partial ^{4} \psi _{j}/\partial x^{4} \simeq [\psi _{j+2} - 4 \psi _{j+1} + 6 \psi _{j} - 4 \psi _{j-1} + \psi _{j-2}]/(\Delta x)^{4}$. The appropriate diffusion coefficient scales with $D_{4} \sim v_{{\mathrm{fastest}}} [\pi / (\Delta x)]^{3}$. An appropriate value for this coefficient is when it is large enough to control numerical issues at the grid scale while not impacting the large scale physics. A good approach to optimize this value is to run multiple simulations with only varying $D_{4}$, and finding a range of values for which the numerics are good and the large scale features are only weakly dependent on $D_{4}$; it is typically within an order of magnitude of the scaling prediction.

We have focused on F3D as an example of a Hall-MHD code because of its algorithmic simplicity and because it has long been used to study reconnection. There are drawbacks to the code. As a finite difference code, it does not capture shocks, and therefore if the number density gets fairly small in any given simulation the code typically crashes. The code performs well up to about 1000-2000 processors on high powered supercomputers; MHD codes without the Hall effect can be made to scale much better to 10s of thousands of processors. It is also restricted to a rectangular geometry with a regular grid.

There are a number of other Hall-MHD codes that have been used to study magnetic reconnection, some of which we gather here. Some codes used to study Hall-MHD reconnection in a rectangular domain have included VOODOO (Huba 2003), HMHD (Lottermoser and Scholar 1997), another code called HMHD (Huang et al. 2011), and the UI Hall-MHD code (Ma and Bhattacharjee 2001). Codes that have been used to study Hall-MHD in the context of planetary magnetospheres (including Earth’s) are a multi-fluid code (Winglee 2004), Block Adaptive Tree Solar-wind Roe Upwind Scheme (BATS-R-US) (Tóth et al. 2008), and Gkyell (Wang et al. 2018). Codes used in the tokamak geometry include NIMROD (Glasser et al. 1999) and M3D-C1 (Jardin et al. 2008).

### Further Extensions of Hall-MHD

Here, we briefly discuss fluid model extensions beyond ideal-MHD that contain the Hall electric field and other terms. We start with terms that were dropped from Eq. (10).

#### Hall-MHD with Electron Inertia

One extension of Hall-MHD is to retain the electron inertia term $m_{e} d{\mathbf{u}}_{e}/dt$ from Eq. (10). We call this model “Hall-MHD with electron inertia;” it is often called the “two-fluid model” in the literature, but we refrain from this nomenclature since a completely different set of equations is also typically given the same name.

Using the electron inertia term as is would lead to a new variable ${\mathbf{u}}_{e}$ with a time derivative in the model. Instead, the standard approach is to recognize that the prefactor $m_{e}$ is small for electron-ion plasmas, so the only way this term important is if the electrons are moving very fast. The ions would be too slow to keep up in such a case, so on time scales where this term is important, we can treat the ions as approximately stationary. Then, the electron bulk flow velocity ${\mathbf{u}}_{e}$ is related to the current density via ${\mathbf{u}}_{e} = - {\mathbf{J}} / n_{e} e$. Since ${\mathbf{J}}$ and $n_{e}$ are already included in the Hall-MHD description, the set of equations remains closed. Such a simplification is convenient but not absolutely necessary. For example, Sect. 4.4 describes a hybrid code in which the ion flows and density effects in the electron inertia term are included.

Analytically, the inertial electric field from dividing Eq. (10) by $-e$ is given by $-(m_{e}/e)d{\mathbf{u}}_{e}/dt$, and replacing ${\mathbf{u}}_{e}$ by $-{\mathbf{J}}/n_{e} e$ gives $(m_{e}/e^{2})d({\mathbf{J}}/n_{e})/dt$. Then, Ohm’s law in Hall-MHD with electron inertia becomes

33 $$ {\mathbf{E}} + \frac{{\mathbf{u}} \times {\mathbf{B}}}{c} = \frac{{\mathbf{J}} \times {\mathbf{B}}}{n_{e} e c} + \frac{m_{e}}{e^{2}} \frac{d}{dt}\left (\frac{{\mathbf{J}}}{n_{e}}\right ). $$

It is important to note that the same approximation ${\mathbf{u}}_{e} \simeq -{\mathbf{J}} / n_{e} e$ is used in the convective derivative term, so that $d/dt \simeq \partial / \partial t - (1/n_{e} e){\mathbf{J}} \cdot \nabla $.

One needs to treat the $\partial / \partial t$ term on the right hand side of Eq. (33). To do so, we eliminate ${\mathbf{E}}$ in Faraday’s law using Eq. (33); some algebra reveals that the equation becomes

34 $$ \frac{\partial {\mathbf{B}}^{\prime}}{\partial t} = - c \nabla \times { \mathbf{E}}^{\prime}, $$

where ${\mathbf{B}}^{\prime }= (1-d_{e}^{2} \nabla ^{2}){\mathbf{B}}$ is an auxiliary magnetic field and ${\mathbf{E}}^{\prime }= {\mathbf{J}} \times {\mathbf{B}}^{\prime }/ nec -{\mathbf{J}}_{i} \times {\mathbf{B}} / nec$ is an auxiliary electric field, and the factor of $n$ in the inertia term is treated as a constant on the time scales of interest. Coupling this equation with Eqs. (25)-(27) gives a closed set of equations.

To solve these equations, we note that Eq. (34) looks just like the usual Faraday’s law except for the primes, so the same numerical technique can be used to solve for ${\mathbf{B}}^{\prime}$. Once ${\mathbf{B}}^{\prime}$ is found, one uses that variable as the known source term in the equation $(1 - d_{e}^{2} \nabla ^{2}) {\mathbf{B}} = {\mathbf{B}}^{\prime}$ to solve for ${\mathbf{B}}$. This is an elliptic differential equation with many algorithms that can be used to solve it. The simplest may be a relaxation technique (Press et al. 1992), but it is relatively slow, especially when used for codes that have been parallelized for use on a supercomputer. A faster version of relaxation is called multigrid (Trottenberg et al. 2000). F3D employs the Fast Fourier Transform approach.

We now briefly discuss the physics introduced by the electron inertia term and the advantages for including it in simulations. By comparing the electron inertia term to the Hall term, we determine the condition under which it is important to retain the electron inertia term. We know the Hall electric field is important at scales below the ion inertial scale $d_{i}$, but vanishes at the X-line where ${\mathbf{B}} = 0$. The electron inertia term can be important at small scales, so we seek the scale at which it becomes comparable to the Hall electric field. Setting them equal gives $J_{y} B_{x} / nec \sim (m_{e}/e^{2}) [({\mathbf{J}}/ne) \cdot \nabla ] (J_{z}/n)$. In the scaling sense, we use $B_{x} \sim B_{up}$, $J_{z} \sim cB_{up}/4 \pi \delta $ where $\delta $ is the scale at which the two terms are equal, so $J_{y} B_{up} / nec \sim (m_{e}/e^{2}) (J_{y}/ne \delta ) (cB_{up}/4 \pi \delta n)$, which simplifies to $\delta ^{2} \sim d_{e}^{2}$, where $d_{e}^{2} = m_{e} c^{2}/ 4 \pi n e^{2}$ is the electron inertial scale. Thus, at length scales below $d_{e}$, the electron inertia term can be important.

Consequently, one reason researchers include the electron inertia term into their Hall-MHD model is to aim to capture electron scale physics more accurately than without it. We point out, however, that the MHD model itself was derived with the assumption that $m_{e} / m_{i}$ is small, and therefore including the electron inertia term as we have done does not actually provide a self-consistent treatment of sub-$d_{e}$ scale physics. The two fluid model or the kinetic approach is needed to more accurately capture electron scale physics.

Then why include electron inertia? The answer is that it helps with the numerics. To see this, we consider waves in the Hall-MHD with electron inertia system, generalizing the treatment in Sects. 3.4.1 and 3.4.2. The dispersion relation for perfectly parallel propagating waves in Hall-MHD with electron inertia, generalizing Eq. (19), becomes

35 $$ \omega ^{2} = \frac{k^{2} v_{A0}^{2}}{D_{e}} \left ( 1 + \frac{k^{2} d_{i0}^{2}}{2 D_{e}} + \sqrt{ \frac{k^{2} d_{i0}^{2}}{D_{e}} + \frac{k^{4} d_{i0}^{4}}{4 D_{e}^{2}}} \right ), $$

where $D_{e} = 1 + k^{2} d_{e}^{2}$. When $k d_{e}$ is negligible, this dispersion relation reduces to the Hall-MHD result in Eq. (19). When $k d_{e} \gg 1$, the waves become electron cyclotron waves with $\omega ^{2} = \Omega _{ce}^{2}$, where $\Omega _{ce} = e B_{0} / m_{e} c$. The reason this is useful numerically is that the whistler wave is dispersive, and in Hall-MHD the phase speed goes to infinity as the wavelength goes to zero. In Hall-MHD with electron inertia, the whistler rolls over to the electron cyclotron wave which does not propagate, so there is a maximum speed of the waves. This means that the time step required to run the simulation does not go to zero, and the simulations with very high resolution are less stiff and therefore cheaper to carry out.

#### Electron Magnetohydrodynamics (EMHD)

The electron-MHD (EMHD) model (Kingsep et al. 1990) is used when the large (MHD) scales are not of interest, and only the scales between electron and ion scales are of interest. In such a limit, the MHD terms concerning ion velocity are dropped. Because of this, the density and pressure can no longer change (on the time scales of interest), so the only remaining governing equation is Faraday’s law, with the electric field given solely by the Hall electric field:

36 $$\begin{aligned} \frac{\partial {\mathbf{B}}}{\partial t} = & - c \nabla \times {\mathbf{E}}, \end{aligned}$$

37 $$\begin{aligned} {\mathbf{E}} = & \frac{{\mathbf{J}} \times {\mathbf{B}}}{n e c}. \end{aligned}$$

Using Ampère’s law ${\mathbf{J}} = (c/4\pi ) \nabla \times {\mathbf{B}}$, it is common to combine these equations into a single equation for ${\mathbf{B}}$,

38 $$ \frac{\partial {\mathbf{B}}}{\partial t} = - \frac{c}{4\pi ne} \nabla \times \left [ (\nabla \times {\mathbf{B}}) \times {\mathbf{B}} \right ]. $$

This closed vector equation comprises the EMHD model. One can include electron inertia in a manner analogous to the full Hall-MHD model: $\partial {\mathbf{B}}^{\prime }/ \partial t = - (1/n_{e} e) \nabla \times ({\mathbf{J}} \times {\mathbf{B}}^{\prime})$, where ${\mathbf{J}} = (c/4\pi ) \nabla \times {\mathbf{B}}$ and ${\mathbf{B}}^{\prime }= (1 - d_{e}^{2} \nabla ^{2}) {\mathbf{B}}$.

#### The Electron Pressure Term in Hall-MHD

We now consider the electron pressure term in Eq. (10). There, electron pressure is written as a tensor ${\mathbf{p}}_{e}$, which is the most general form following directly from the Vlasov/Boltzmann equation in kinetic theory. Retaining it in Ohm’s law would give a term on the right hand side of Eq. (33) of the form $-(1/n_{e} e) \nabla \cdot {\mathbf{p}}_{e}$. We now consider a few commonly used approximations to simplify this.

##### Isotropic Electron Pressure

In MHD, the total pressure is assumed to be isotropic, so that ${\mathbf{p}} = p {\mathbf{I}}$. In this case, the term entering Ohm’s law is $-(1/n_{e} e) \nabla p_{e}$. When substituted into Faraday’s law [Eq. (16)], this term gives a contribution of $(c/e) \nabla \times (\nabla p_{e}/n) = (c/e) \nabla (1/n_{e}) \times \nabla p_{e} = -(c/en_{e}^{2}) \nabla n_{e} \times \nabla p_{e}$. This contribution to the electric field is called the “Biermann battery” (Biermann 1950) and it is often of great importance in reconnection in high energy density plasmas (Fox et al. 2012). In most space applications that employ a fluid model, the electrons are assumed to be adiabatic with $p_{e}/n_{e}^{\gamma}$ equal to a constant or isothermal with $p_{e}/n_{e}$ equal to a constant. In either limit, the Biermann battery term vanishes identically.

One may presume from this result that the electron pressure gradient term does not have any contribution to Hall-MHD, but this is not true. Including the scalar electron pressure gradient in the Hall-MHD Ohm’s law gives

39 $$ {\mathbf{E}} + \frac{{\mathbf{u}}_{e} \times {\mathbf{B}}}{c} = -\frac{1}{n_{e} e} \nabla p_{e}, $$

where we write ${\mathbf{u}}_{e} \simeq {\mathbf{u}} - {\mathbf{J}} / n_{e} e$ for the bulk electron velocity. Taking the cross product of this equation with ${\mathbf{B}}$ and solving for the bulk flow velocity ${\mathbf{u}}_{e,\perp}$ perpendicular to the magnetic field ${\mathbf{B}}$ gives

40 $$ {\mathbf{u}}_{e \perp} = c \frac{{\mathbf{E}} \times {\mathbf{B}}}{B^{2}} + \frac{c}{n_{e} e B^{2}} \nabla p_{e} \times {\mathbf{B}}. $$

This equation implies the electron perpendicular bulk flow velocity is a sum of the $E \times B$ drift $c {\mathbf{E}} \times {\mathbf{B}} / B^{2}$ and the electron diamagnetic drift speed ${\mathbf{u}}_{*e} = c \nabla p_{e} \times {\mathbf{B}} / n_{e} e B^{2}$. This important result shows that the electron diamagnetic drift is captured in Hall-MHD provided the electron pressure is non-zero. An important implication of this is that magnetic flux convects at the sum of the $E \times B$ and electron diamagnetic drift speed when the electron pressure is retained in Ohm’s law.

##### Gyrotropic Electron Pressure

The next level of approximation for the electron pressure tensor is motivated by the fact that electrons in a magnetic field often have a different temperature in the directions parallel and perpendicular to the magnetic field, *i.e.,* the electrons are gyrotropic. The electron pressure parallel to the magnetic field is $p_{e,\|}$ and perpendicular to the magnetic field is $p_{e,\perp}$. The general way to write a gyrotropic electron pressure tensor ${\mathbf{p}}_{e,g}$ for a magnetic field in an arbitrary direction ${\mathbf{\hat{b}}} = {\mathbf{B}} / B$ is (Parker 1957a)

41 $$ {\mathbf{p}}_{e,g} = p_{e,\perp} {\mathbf{I}} + (p_{e,\|}-p_{e,\perp}) {\mathbf{\hat{b}}} {\mathbf{\hat{b}}}. $$

Using this form in the generalized Ohm’s law for the electron pressure, and an analogous term in the pressure gradient force in the momentum equation allows for the modeling of a plasma with a gyrotropic pressure.

Having a closed set of equations requires closures on the parallel and perpendicular pressures. The most widely known closure is the Chew-Goldberger-Low (CGL) closure, which assumes that there is no heat flux and the plasma is magnetized (Chew et al. 1956). A direct calculation using the Vlasov equation and these assumptions gives the CGL “double adiabatic laws”

42 $$ \frac{d}{dt} \left ( \frac{p_{\perp}}{n B} \right ) = 0, \hspace{0.5cm} \frac{d}{dt} \left ( \frac{p_{\|} B^{2}}{n^{3}} \right ) = 0, $$

A second model of great importance to reconnection is the Egedal closure (Egedal et al. 2013). This arises in the upstream and downstream regions of magnetic reconnection regions. The key physics is that the magnetic fields in this region are mirror fields that can trap electrons. The presence of an electric field heats them parallel to the electric field, leading to elongated gyrotropic distributions in the parallel direction. For large magnetic field strength, the equations reduce to the isothermal equation of state. For small magnetic field strength, the equations reduce to the CGL equations. The two limits were interpolated to find a closure that could be implemented into a fluid code; see Egedal et al. (2013) for details.

### Examples of Reconnection Simulation Results with the Hall Electric Field

#### Ion-Coupled Reconnection

**Fig. 3:**
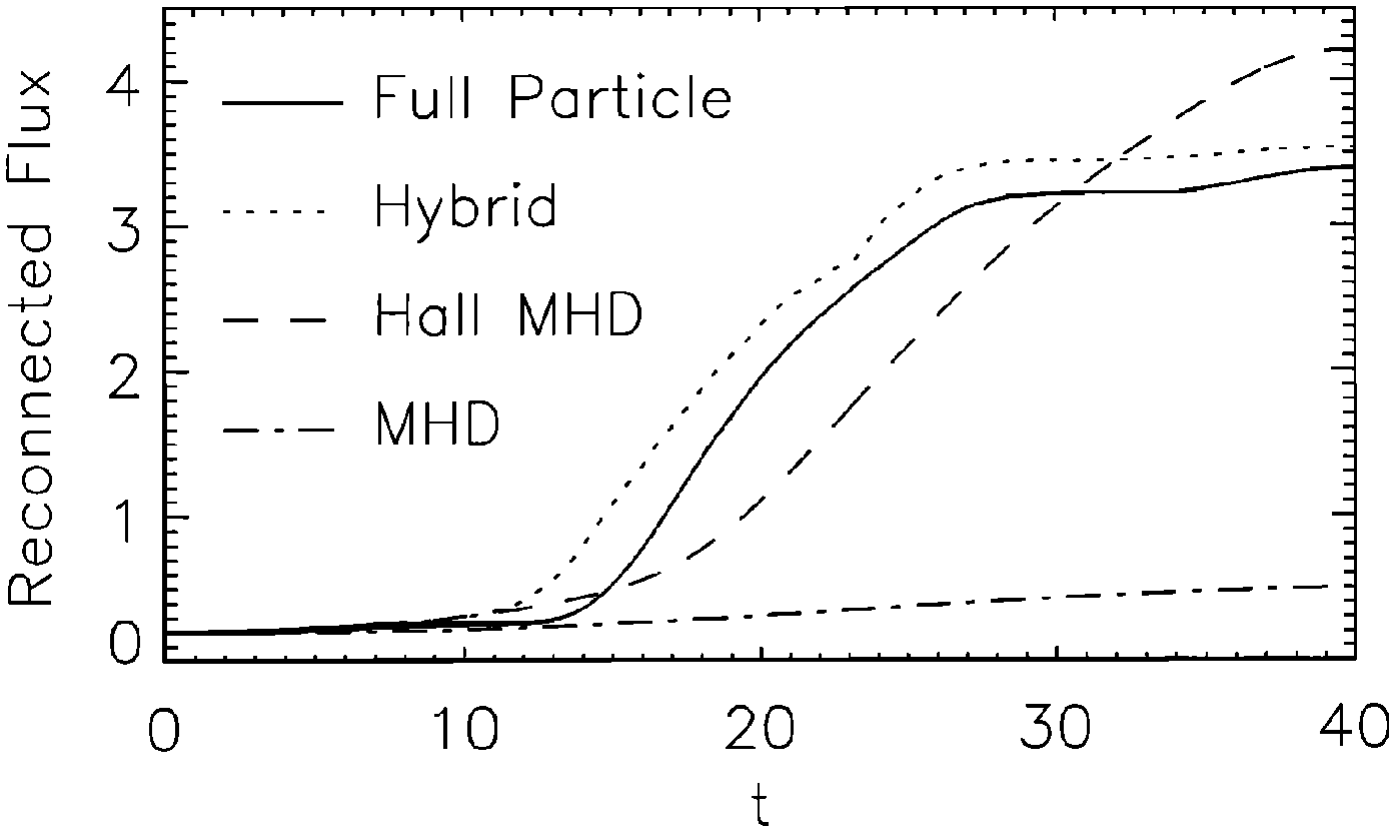
**“GEM Challenge” result showing Hall-MHD simulations faithfully obtain the rate of change of reconnected flux obtained in kinetic models.** The legend describes the simulation approach for each curve; resistive-MHD is far slower than the other models. Adapted from Birn et al. (2001)

The Hall-MHD model holds a significant place of importance in the history of reconnection simulations. From the earliest days of reconnection research, it was known that the Sweet-Parker model (Sweet 1958; Parker 1957b) was too slow to explain solar flares (Parker 1963), and it is also too slow to explain magnetotail reconnection (Parker 1973) and the sawtooth crash in tokamaks (Edwards et al. 1986; Yamada et al. 1994). It was discovered that using a localized resistivity leads to reconnection fast enough to explain the observed reconnection rates (Ugai and Tsuda 1977; Sato and Hayashi 1979), but the resistivity model was not derived from first principles and it remains inconclusive whether it can be. Then, reconnection with the Hall electric field was found to also produce rates comparable to observed values without relying on ad hoc terms (Aydemir 1992; Wang and Bhattacharjee 1993; Kleva et al. 1995; Ma and Bhattacharjee 1996). The “GEM Challenge” study ((Birn et al. 2001) and references therein), one of the most cited papers ever about reconnection, compared comparable simulations with different simulation models, and all models containing the Hall electric field led to fast reconnection with a reconnection rate approximately 0.1, as shown in Fig. 3. We note that this figure contains results from Hybrid and PIC simulations, which are described in Chaps. 4 and 5 respectively. It was only recently that an explanation of why the Hall electric field contributes to make the reconnection rate 0.1 was presented (Liu et al. 2022). Thus, Hall-MHD represents the minimal first-principles physics model that reproduces the reconnection rate achieved in kinetic models.

**Fig. 4:**
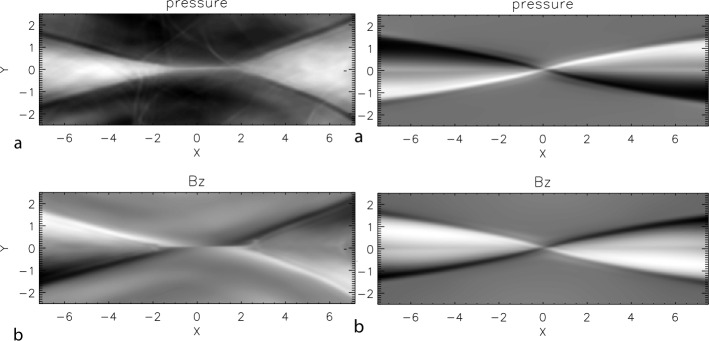
**Pressure and out-of-plane magnetic field in Hall MHD reconnection simulations.** The legend describes the simulation approach for each curve; resistive-MHD is far slower than the other models. Adapted from Rogers et al. (2003)

Another characteristic feature of collisionless reconnection is the quadrupolar structure of the out-of-plane magnetic field. For anti-parallel reconnection, it was posited that the Hall electric field would cause there to be a quadrupolar out-of-plane magnetic field due to the in-plane currents within the ion diffusion region (where the ions decouple from the magnetic field but the electrons remain frozen-in) (Sonnerup 1979). Although this is a completely nonlinear effect, it is analogous to the out-of-plane magnetic field generation in the whistler wave shown in Fig. 2(b) (Drake and Shay 2007). It can also be thought of as the out-of-plane current dragging the reconnecting field out of the reconnection plane (Mandt et al. 1994). Hall-MHD simulations of reconnection produce a quadrupolar out-of-plane magnetic field (Huba and Rudakov 2002), as shown in the lower left plot in Fig. 4 from simulations in Rogers et al. (2003). When there is a strong out-of-plane (guide) magnetic field, the quadrupolar structure persists, but a quadrupolar structure in the gas pressure also arises with opposite polarity (Kleva et al. 1995). The physical reason is analogous to the gas pressure perturbation formation in kinetic Alfvén waves as shown in Fig. 2(c). These quadrupolar structures arise in Hall-MHD simulations of reconnection, as shown in the right two panels of Fig. 4 from a simulation with guide field three times as strong as the reconnecting magnetic field. Despite the ability to produce quadrupolar structure in these quantities, it is now known that the detailed structure of the quadrupolar structures is not precisely the same as observed in kinetic simulations (Shay et al. 2007; Karimabadi et al. 2007) or magnetospheric observations (Phan et al. 2007). It was shown that the inclusion of an electron pressure anisotropy leads to better agreement with kinetic modeling (Ohia et al. 2012). An electron pressure anisotropy with the CGL relations and without the Hall term can also produce reconnection rates comparable to Hall reconnection (Cassak et al. 2015).

Another aspect of Hall-MHD reconnection that is not captured in collisional (Sweet-Parker) reconnection is how reconnection that is localized in the out-of-plane direction spreads in that direction. Spreading has been studied in a number of Hall-MHD studies (Huba and Rudakov 2002; Shay et al. 2003; Huba and Rudakov 2003; Karimabadi et al. 2004; Nakamura et al. 2012; Shepherd and Cassak 2012; Arencibia et al. 2021, 2023) and EMHD studies (Jain et al. 2013) and they agree with kinetic simulations of reconnection spreading (Lapenta et al. 2006). In particular, it was shown that anti-parallel reconnection does not spread in resistive-MHD, but it does spread in Hall-MHD and kinetic models (Nakamura et al. 2012; Arencibia et al. 2021).

#### Electron-Only Reconnection

Collisionless magnetic reconnection in electron scale current sheets has been investigated using fixed ion models (both fluid and kinetic) for several decades before the observational discovery of electron-only reconnection by MMS (Bulanov et al. 1992; Mandt et al. 1994; Drake et al. 1994, 1997; Shay and Drake 1998; Attico et al. 2000; Chacón et al. 2007; Zocco et al. 2009; Jain and Sharma 2009; Jain et al. 2012; Jain and Büchner 2015; Jain and Sharma 2015a,b). It was, however, not termed as electron only reconnection as it was not conceived at that time that reconnection without ion participation is possible. It was rather termed as “early phase of reconnection” (Jain and Sharma 2009; Jain et al. 2012; Jain and Sharma 2015a,b). Later, electron only reconnection in Earth’s magnetotail was also interpreted as an early phase of the standard reconnection (Hubbert et al. 2022; Farrugia et al. 2021) and there is ongoing discussion if the observed reconnection events without ion coupling are electron-only reconnection events or just an early phase of standard reconnection (Lu et al. 2020, 2022; Wang et al. 2020b; Yi et al. 2022).

Jain and Sharma (2009) were the first to propose that the early phase of reconnection after its onset in electron scale current sheets will be dominated by electron dynamics without coupling to ion dynamics and carried out EMHD simulations to study the physics of the early phase. This study was followed by further theoretical and simulation studies, both in 2-D and 3-D, using EMHD model and comparison with space observations by Cluster spacecraft (Jain et al. 2012; Jain and Sharma 2015a,b; Jain and Büchner 2014a,b, 2015; Jain et al. 2017b,a). These and other EMHD studies are also relevant for electron only reconnection.

**Fig. 5:**
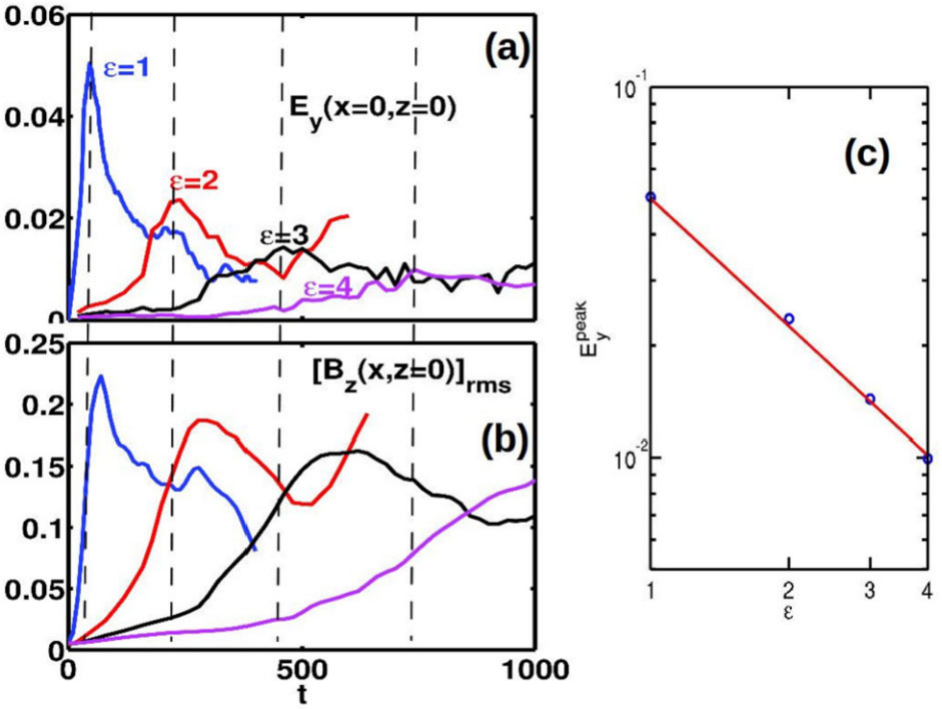
Evolution of (a) out-of-plane electric field $E_{y}$ at the X-point ($x=0$, $z=0$) and (b) root-mean-square value of the normal component of magnetic field $B_{z}$ evaluated over the length of the reconnecting current sheet (between two outflow regions) Vertical dashed lines mark the times at which $E_{y}$ attains its peak value $E_{y}^{peak}$ for different values of the current sheet half-thickness $\epsilon $. (c) Scaling of $E_{y}^{peak}$ with $\epsilon $ (in log -scale) from simulations (blue circles) and a fit $E_{y}^{peak}=0.05/\epsilon ^{1.15}$ (red line). Adapted from Jain and Sharma (2015b)

Figure 5 shows results from the 2-D EMHD (x-z plane) simulations of magnetic reconnection in electron scale current sheets of different half-thicknesses ($\epsilon $) (Jain and Sharma 2015b). In these simulations, the equilibrium current density is $\mathbf{J}=-n_{0}eu_{ey0}\hat{y}=(B_{0}\,c/4\pi \,\epsilon ) \, \mathrm{sech}^{2}(z/\epsilon )\hat{y}$ corresponding to the equilibrium anti-parallel magnetic field $\mathbf{B}= B_{0}\tanh (z/\epsilon ) \hat{x}$. The density $n_{0}$ is uniform and thus the current is due to the electron flow $u_{ey0}$. The results are shown in the normalized units: length by electron inertial length $d_{e}=c/\omega _{pe}=c/(4\pi n_{0} e^{2}/m_{e})^{1/2}$, magnetic field by $B_{0}$ and time by $\omega _{ce}^{-1}=(e B_{0}/m_{e}c)^{-1}$. Reconnection rate, measured by out-of-plane electric field $E_{y}$ at the X-point and shown in Fig. 5a, reaches its peak value when the growth of the rms values of $B_{z}$ begins to slow down, consistent with the Faraday law which gives $\partial E_{y}/\partial x=-1/c\partial B_{z}/\partial t$. The peak reconnection rate $E_{y}^{peak}$ scales with $\epsilon $ as $E_{y}^{peak}=0.05/\epsilon ^{1.15}$ and drops from $E_{y}^{peak}=0.05\, v_{Ae} B_{0}/c$ to $E_{y}^{peak}=0.01\, v_{Ae} B_{0}/c$ as the $\epsilon $ increases from $\epsilon =d_{e}$ to $\epsilon =4\,d_{e}$. This range of reconnection rate in units based on ion Alfvén speed is $E_{y}^{peak}=0.43-2.15\, v_{Ai} B_{0}/c$ (using ion $m_{i}/m_{e}=1836$) which is much larger than the value of the reconnection rate ($0.1\,v_{Ai} B_{0}/c$) for the standard ion-coupled reconnection. Reconnection rates for the electron only reconnection have been reported in the similar range by MMS observations (Burch et al. 2020) and PIC simulations (Sharma Pyakurel et al. 2019). A recent analytical model (Liu et al. 2025b) that incorporates the dispersive properties of Alfvén waves (Sect. 3.4) was proposed to explain these higher electron-only reconnection rates.

Note that the EMHD simulation results in Fig. 5 are independent of the strength of the guide magnetic field because a uniform guide field does not appear in 2-D EMHD equations (Jain and Büchner 2015). However, in 3-D, guide field can introduce current aligned instabilities in addition to the tearing instability as has been predicted by 3-D EMHD eigen value analysis (Jain and Büchner 2015) and simulations (Jain et al. 2017a). In 3-D, current aligned electron Kelvin-Helmholtz instabilities can grow in electron scale current sheets even in the absence of guide magnetic field (Jain and Büchner 2014a,b; Greess et al. 2021). These EMHD studies are relevant for the MMS observations of the electron shear flow generated electron Kelvin-Helmholtz vortices within the diffusion region of collisionless magnetic reconnection (Zhong et al. 2022, 2018; Hwang et al. 2019).

### The Future of Hall-MHD

Interestingly, many computational plasma physicists in the reconnection community are moving away from the Hall-MHD model and its fluid extensions to study the small-scale properties of reconnection. The Hall-MHD model was crucial for understanding the minimal physics that gives rise to a 0.1 reconnection rate. As questions have moved to other aspects of reconnection including heating and particle acceleration, many researchers opt for kinetic models to more realistically capture the small scale physics than can be done with Hall-MHD. Treatments of particle acceleration and heating at large scales (Arnold et al. 2021) do not require the Hall electric field.

As example of an avenue of modern reconnection research and modeling where the Hall-MHD model remains highly beneficial is in the MHD-EPIC approach to global magnetospheric modeling (Daldorff et al. 2014), as is discussed more fully in Sect. 6. In this approach, the fluid model is used in regions of the magnetosphere where no small important scale physics takes place, which allows for faster run times. In regions where small scale physics does take place, the code couples to a particle-in-cell code that captures this physics. The numerical results between the two models are passed back and forth to each other across their boundaries. The Hall-MHD model is well suited to be used in the transition region between the PIC and MHD models to facilitate a more accurate transition between the two models. The Hall-MHD model has been used to study global magnetospheric systems for Earth and other planets and moons to great effect (Paty and Winglee 2004; Dorelli et al. 2015; Dong et al. 2019; Li et al. 2023b), and it is anticipated that further advances will continue to be made with the Hall-MHD approach for systems too large to employ global kinetic codes.

## Hybrid Simulations

The Earth’s magnetosphere is a complex plasma system characterized by a multitude of multiscale processes governing the interaction of the solar wind with the Earth’s dipole magnetic field. Modeling small-scale turbulent processes in the foreshock and magnetosheath requires inclusion of ion kinetic effects and Hall physics (Karimabadi et al. 2014; Omelchenko et al. 2021a). Furthermore, kinetic treatment of hot and cold ion populations is greatly needed for improved modeling of ionospheric outflows and their impact on the magnetopause and magnetotail. To study magnetic reconnection, one also needs to incorporate finite electron-mass effects (e.g. Biskamp 2000; Birn and Priest 2007; Gonzalez and Parker 2016), or mimic these effects with *ad hoc* (resistivity) models.

The necessity to account for smaller and faster scales in kinetic simulations in a manner that would guarantee their numerical accuracy and computational efficiency creates challenges in global modeling of the Earth’s magnetosphere. Since describing plasma kinetics with pure “first-principles” models is still not feasible, various approximations have been developed as candidates for future “beyond MHD” operational modeling, with multiple levels of physical fidelity included. However, to what degree kinetic processes may influence the “fluid-like” behavior of the magnetosphere on global scales still remains an open question. As we argue below, many of these challenges can be addressed by self-consistent hybrid modeling, where Maxwell’s equations are solved in the quasi-neutral Darwin limit, ion species are treated kinetically, and the plasma electrons are approximated as an inertialess fluid. These hybrid models can be broken into two categories, according to the computational techniques used to represent kinetic ions: Particle-in-Cell (PIC) models (Winske et al. 2003; Lipatov 2002) and Vlasov models (von Alfthan et al. 2014). In what follows we discuss only the hybrid-PIC approach because it has already been applied successfully to perform three-dimensional (3D) simulations of global plasma systems that range from the Earth’s magnetosphere (e.g. Lin and Wang 2005; Omelchenko et al. 2021a), planets (Herčík et al. 2013; Jarvinen et al. 2020), and small space bodies (Fatemi et al. 2017; Kallio et al. 2019) to compact laboratory plasmas (Omelchenko and Sudan 1997; Lin et al. 2008; Thoma et al. 2013; Omelchenko 2015; Omelchenko and Karimabadi 2022). The hybrid-Vlasov approach (von Alfthan et al. 2014) is relatively new and considerably more computationally expensive, with production runs being still restricted to quasi-3D setups (Pfau-Kempf et al. 2020).

An important issue to grasp reconnection correctly is the consideration of the finite electron inertia, as it has been shown by EMHD simulations. This could reveal not only the properties of electron-only reconnection, proposed by (Jain and Sharma 2009) and recently discovered by MMS in the magnetsheath (Phan et al. 2018), but also the transition from electron- to ion reconnection. These effects can be treated by hybrid-approach with kinetic ions and an inertial electron fluid (Sect. 4.4).

### Model Equations: Massless Electrons

The standard hybrid model (Winske et al. 2003) assumes plasma quasi-neutrality, neglects the displacement current in Maxwell’s equations, and treats ions as full-orbit macro-particles (in the PIC approach) moving in self-consistent electric and magnetic fields. The plasma electrons are approximated as an inertialess fluid with scalar pressure described by either an adiabatic law or evolution equation. Together with a self-consistent PIC method for the ion components, this leads to a set of hybrid equations that include Ampere’s law in the magnetostatic limit, Faraday’s law, and an algebraic expression for electric field (generalized Ohm’s law) with the Hall, electron pressure gradient, and resistive terms (e.g. Omelchenko and Karimabadi 2012):

43 $$ \frac{d\mathbf{x}_{i}}{dt} =\mathbf{v}_{i}, $$

44 $$ m_{i}\frac{d\mathbf{v}_{i}}{dt} = q_{i}\,(\mathbf{E} + \frac{\mathbf{v}_{i}\times \mathbf{B}}{c}), $$

45 $$ \nabla \times {\mathbf{B}}=\frac{4\pi}{c}{\mathbf{J}}, ~{\mathbf{J}}={\mathbf{J}}_{e}+{ \mathbf{J}}_{i}, $$

46 $$ \frac{\partial {\mathbf{B}}}{\partial t}=-c\nabla \times{\mathbf{E}}, $$

47 $$ {\mathbf{E}} = \frac{{\mathbf{J}}_{e}\times {\mathbf{B}}_{t}}{en_{e}c} - \frac{\nabla p_{e}}{en_{e}}+{\eta {\mathbf{J}}}, ~{\mathbf{B}}_{t}={\mathbf{B}}+{ \mathbf{B}}_{ext}, $$

48 $$ en_{e}=\rho _{i}, $$

49 $$ p_{e}=n_{e}T_{e}\sim n_{e}^{\gamma}. $$

Eqs. ((43) and (44)) are the equations of motion for each ion particle. $q_{i}$ is the charge on each ion, and in these equations the electric and magnetic fields are interpolated from the grid onto the each particle’s position. In Eqs. (47)-(49) $n_{e}$, ${\mathbf{J}}_{e}$ are the electron number and current density, respectively; $p_{e}$ is the electron pressure, here assumed to governed by Eq. (49)) with an adiabatic constant of $\gamma $; $T_{e}$ is the electron temperature; $\rho _{i}$, ${\mathbf{J}}_{i}$ are the total ion charge density and current density (found by summing up individual particles around each grid point); ${\mathbf{E}}$ is the electric field; ${\mathbf{B}}$, ${\mathbf{B}}_{ext}$ are the “self-generated” (${\mathbf{B}}\rvert _{t=0}=0$) and “external” (steady state) magnetic fields, respectively.

The applied plasma resistivity, $\eta $ is either constant or chosen to be a function of plasma parameters (e.g. Lin et al. 2007; Omelchenko et al. 2021a). The resistive term in the generalized Ohm’s law (Eq. (47)) may (i) describe finite conductivity of plasma or space bodies (e.g., the Moon (Fatemi et al. 2017; Omelchenko et al. 2021b)), (ii) imitate finite electron inertia effects in magnetic reconnection events, and (iii) enable fast magnetic field diffusion at low-density (“vacuum”) cells, $n_{e} \leq n_{min}$, where $n_{min}$ is a small density cutoff value (Omelchenko et al. 2021b). Failure to properly treat low-density regions in hybrid simulations may lead to non-physical results (Omelchenko et al. 2021b; Poppe 2019).

### Key Physics Beyond MHD in Hybrid Models

The “mesoscale” hybrid model occupies the middle ground between the “large-scale” fluid and “micro-scale” first-principles modeling paradigms. For global magnetospheric simulations, the hybrid model enables a number of “beyond MHD” capabilities, as explained below.

***Modeling turbulent processes in the foreshock and magnetosheath.*** Unless large resistive damping (or smoothing) is applied, the hybrid model accurately captures the Hall physics for mesh cell sizes, $\Delta \leq d_{i}$, where $d_{i}=c/\omega _{pi}$ and $\omega _{pi}$ are the local ion inertial length and plasma frequency, respectively. The Hall effects phase out on coarser meshes, $\Delta \gg d_{i}$, where the Alfvén term becomes greater than the Hall term in Eq. (47) and the whistler mode frequency, $\propto \Delta ^{-2}$ becomes lower than the Alfvén mode frequency, $\propto \Delta ^{-1}$. In fact, in this case the Hall term can completely be removed from the electric field in Faraday’s law (Eq. (46)) and kept only in the equations of ion motion (Karimabadi et al. 2004). The ability of a hybrid code with the Hall term to run stably on coarser meshes ($\Delta \gtrsim d_{i}$) may depend on the numerical implementation of Faraday’s law (Omelchenko and Karimabadi 2012).

Global hybrid codes have been used to address the ultra-low frequency (ULF) physics of the curved bow shock on the ion inertial/Larmor radius scales as the physics of the bow shock is predominantly determined by kinetic physics associated with charged particles from the solar wind. Of particular interest are the foreshock waves and diffuse ion distributions (Wang et al. 2009) and transient perturbations originating from the wave-particle processes in the quasi-parallel shock or due to the shock interaction with incoming solar wind discontinuities, including hot flow anomalies (Lin 2002; Lin et al. 2022b), foreshock bubbles (Omidi et al. 2010; Wang et al. 2020a), foreshock cavities (Lin and Wang 2005; Blanco-Cano et al. 2011), and high-speed jets (Omelchenko et al. 2021a; Palmroth et al. 2018b). The 3D hybrid simulations with ANGIE3D (AuburN Global hybrId CodE in 3-D) link the foreshock perturbations to the surface perturbations and kinetic-scale shear Alfvén waves (KAWs) at the magnetopause through mode conversion from the incoming compressional waves (Lin and Wang 2005; Shi et al. 2013), as well as the subsequent excitation of toroidal-mode field line resonances in the magnetosphere (Shi et al. 2021). It has also been shown that 3D models are essential for addressing the nonlinear physics of mode coupling and ion diffusion at the magnetopause (Lin et al. 2012).

The whistler mode plays a significant role in regulating turbulence in the magnetosheath and mediating magnetic reconnection in the Earth’s magnetosphere (e.g. Dorelli and Birn 2003; Drake et al. 2008). Hybrid simulations generally have to resolve the quadratic dispersion of this mode, $\omega \propto k^{2}$. This requirement may create computational bottlenecks in simulations of strongly inhomogeneous magnetospheric and laboratory plasmas, where whistler timescales typically span several orders of magnitude (Omelchenko and Karimabadi 2012, 2022). If not accurately integrated in time (or resistively damped), the spurious short-wavelength oscillations may grow explosively unstable from noise and terminate simulation (Lin et al. 2008). It should also be noted that although particle noise in hybrid-PIC simulations typically degrades their physical resolution, the Lagrangian (particle) approach enables transport of ion species with less numerical diffusion compared to the Eulerian approach to solving the Vlasov equation on velocity meshes (von Alfthan et al. 2014).

***Collisionless reconnection at the magnetopause and in the tail plasma sheet.*** The physics of magnetic reconnection in the magnetosphere can be investigated by carrying out global hybrid simulations with an ad-hoc current-dependent resistivity. For the dayside magnetopause, the modeling topics include the structures of ion diffusion region and outflow regions (Tan et al. 2011), global evolution of flux transfer events (FTEs) and magnetic flux ropes (Omidi and Sibeck 2007; Guo et al. 2020, 2021d), propagation of kinetic Alfvén waves and Poynting flux from reconnection (Wang et al. 2019), ion cusp precipitation and energy spectrum (Omidi and Sibeck 2007; Tan et al. 2012), the triggering of reconnection by solar wind discontinuities (Omidi et al. 2009; Pang et al. 2010; Guo et al. 2021c), and magnetosheath turbulence (Chen et al. 2021; Ng et al. 2021). Likewise, high-latitude reconnection tailward of the cusp under northward IMF has also been simulated (Lin and Wang 2006; Guo et al. 2021e). The 3D global physics of storm-time magnetotail reconnection, fast flow and entropy bubbles, the Hall-effects control of dawn-dusk asymmetry (Lin et al. 2014; Lu et al. 2016; Lin et al. 2017), and the associated global Alfvénic coupling between the magnetotail and the ionosphere under southward IMF have been simulated using the ANGIE3D code (Cheng et al. 2020). An Attempt has also been made to investigate the subsequent connection of fast flows to the ring current and radiation belt by combining ANGIE3D with the Comprehensive Inner Magnetosphere-Ionosphere (CIMI) model (Lin et al. 2021).

***Inclusion of multi*-*species plasma ion populations of solar wind origin and improved representation of ionospheric outflow.*** In general, global hybrid models may include multiple ion species for representing solar wind and ionospheric outflow plasmas. Ionospheric outflow ions should be treated kinetically and self-consistently in order to properly account for their impact on the Earth’s magnetosphere. Multi-fluid MHD models do not account for ion resonance acceleration and cyclotron effects, especially for heavy ions (Toledo-Redondo et al. 2021). Self-consistent 3D hybrid simulations of the impact of oxygen outflow on the magnetotail configuration and stability have recently been performed with the HYPERS code (Mouikis et al. 2021; Omelchenko et al. 2023).

***Modeling local reconnection and electron scale physics.*** In MHD simulations, magnetic reconnection is often a result of mesh-dependent diffusion that is difficult to control numerically. The hybrid-PIC model is inherently more robust in this regard because ions are modeled as Lagrangian particles. As a result, reconnection onset and dynamics are controlled by the Hall physics and parameter-dependent resistivity. The hybrid-PIC model is also known to accurately reproduce reconnection rate when the ion inertial and cyclotron scales are properly resolved (Stanier et al. 2015). Further modifications of the hybrid model, which for instance may incorporate finite electron mass effects (e.g. Omelchenko et al. 2021c), could increase physical fidelity of reconnection modeling in the future.

***Modeling non*-*MHD waves in a global context.*** The standard hybrid model supports ion cyclotron, whistler, and kinetic-Alfvén wave modes, which play an important role in regulating plasma turbulence in the Earth’s magnetosphere and impact its global behavior. Modern observations report streams of non-Maxwellian ions that excite plasma turbulence through numerous kinetic instabilities that cannot be modeled within MHD. Global hybrid modeling naturally incorporates the ion kinetic effects into global models of the Earth’s magnetosphere, which helps advance our understanding of the effects of turbulent plasma dynamics on global physical processes.

***Other applications.*** 3D hybrid codes in space physics have been used to simulate shock-driven ion acceleration (Caprioli 2014; Guo and Giacalone 2013), solar wind turbulence (Franci et al. 2018; Roytershteyn et al. 2015), the Moon’s wake (Fatemi et al. 2017; Kallio et al. 2019; Omelchenko et al. 2021b), planetary magnetospheres (Jarvinen et al. 2020), and small space bodies (Alho et al. 2019; Delamere 2009). Comparing results from full-scale 3D hybrid simulations of small space bodies with satellite observations provides yet another important route for validation and further extension of the hybrid approach to plasma modeling. Importantly, the recent advances in ionosphere-magnetosphere coupling (Lin et al. 2022b), code optimization (Dong et al. 2021) and multiscale computing (Omelchenko et al. 2021a) have greatly improved the prospects for hybrid simulations to become a key factor to consider in the overall theory of global solar wind-magnetosphere-ionosphere interactions.

### Numerics

Equations (47)-(49), together with the self-consistent equations of motion for the ion macro-particles, may present computational challenges when used for modeling complex 3D plasma systems, such as the Earth’s magnetosphere. Below we discuss some recent computational advances aimed at overcoming these problems.

#### Spatial Scales

Hybrid-PIC simulations typically intend to resolve the spatial scales of the order of the ion inertial length, $d_{i}$ and ion cyclotron radius, $r_{ci}$. The physical validity regime of the hybrid model ranges from large MHD scales down to $k r_{ci} \sim 1$ and $\omega t \sim 1$. The actual physical resolution of a hybrid simulation is largely determined by (i) how well these characteristic lengths are resolved on a mesh, (ii) how many particles are used. For the typical solar wind proton inertial length, $d_{i} \sim 100~km$, the Earth’s radius, $R_{E}\sim 60d_{i}$. To encompass the whole magnetosphere, the computational mesh in a global simulation should cover the magnetopause with a typical standoff distance, $R_{MP}\sim 10R_{E} \sim 600d_{i}$ and the magnetotail stretching from the Earth to far distances, $R\sim 100R_{E} \sim 6000d_{i}$. The need to accurately account for the “far-field” inflow and outflow boundary conditions in the presence of a magnetic dipole may additionally require multiplying these dimensions by a factor of 2-3. Approximating such large 3D domains with uniform meshes with cell sizes of the order of $\sim 1d_{i}$ is prohibitively expensive for the hybrid model because of the need to advance ions and fields at all cells on kinetic scales.

To overcome these restrictions, several options are available. First, one may increase the cell size beyond $1d_{i}$ at the expense of lower accuracy in resolving the Hall physics (Omelchenko and Karimabadi 2012). Second, one may artificially increase the ratio between the solar wind ion inertial length $d_{i}$ ($d_{i}\sim r_{ci}$ for the outer magnetosphere regions with ion $\beta \sim 1$) and the magnetopause distance $R_{MP}$, in order to better accommodate the available computation resources while still choosing a sufficiently large value of $R_{MP}/d_{i}$ for assuring the separation between the global and local-kinetic scales (Omidi et al. 2004). Both approaches efficiently “downscale” the Earth’s magnetosphere. For instance, ANGIE3D (Lin et al. 2014; Lin and Wang 2005) does it by artificially reducing the solar wind plasma density, i.e. by inflating the characteristic inertial ion length and proportionally increasing the Alfvén speed. Alternatively, H3D (Karimabadi et al. 2014), hybrid-VPIC (Dong et al. 2021), and HYPERS (Omelchenko and Karimabadi 2012) employ the physical ion inertial length but scale the realistic magnetopause standoff distance down by a factor of 4-6 by using a weaker magnetic dipole.

Regardless of a chosen magnetosphere scaling method, present-day 3D hybrid codes typically use $R_{MP}/d_{i}\geq 100$. One of the largest 3D hybrid simulations to date was performed with HYPERS for $R_{MP}/d_{i} \simeq 160$ on a uniform mesh with approximately $1000 \times 2000 \times 2000$ cells (Omelchenko et al. 2021b). To speed up global 3D simulations, hybrid codes may employ nonuniform meshes, among which ‘stretched” (logically mapped) Cartesian meshes are the simplest. Nonuniform meshes typically maintain high resolution in a central domain of interest, while expanding cells towards domain boundaries (Omelchenko et al. 2021a; Lin and Wang 2005) to guarantee that the dipole field vanishes at the inflow/outflow (GSM X) and lateral (GSM Y and Z) boundaries so that robust local boundary conditions can be implemented. Sometimes, for simplicity, the lateral domain boundaries may be assumed to be periodic (e.g. Turc et al. 2015; Müller et al. 2011). This simplification, however, makes a global simulation valid for shorter simulation periods, until reflected particles or electromagnetic perturbations reach the periodic boundaries. To improve the counting statistics for macro-particles, splitting techniques may be used to enhance energetic particle distributions in the dayside magnetosphere (Omelchenko et al. 2021a) and magnetotail (Lin et al. 2007; Wang et al. 2009).

To further reduce the number of computational cells in global simulations, one may employ curvilinear (e.g., spherical) meshes (e.g. Dyadechkin et al. 2013; Guo et al. 2021b). For example, to capture the short-wavelength physics along the shock normal and the magnetopause, early hybrid simulations, focusing on the dayside regions, employed cylindrical (2D) (Swift 1996; Lin et al. 1996; Lin 2002) and spherical (3D) (Lin and Wang 2005) coordinate systems. The spherical coordinate lines, however, have a singularity on the polar axis, which was handled by rotating the polar coordinates to the equator and omitting a conic region around them, while keeping the physical polar regions inside the domain (Lin and Wang 2005). A 2D hybrid simulation of the magnetotail also used curvilinear coordinates to accommodate the tail geometry (Swift and Lin 2001; Lin 2002). Similar to the need of assuring proper numerical resolution for resolving the kinetic scales along the curved or oblique boundary surfaces on the Cartesian meshes, special care is also necessary for the curvilinear meshes, especially when their coordinate lines are not orthogonal (Swift and Lin 2001). Compared to Cartesian meshes, curvilinear meshes may introduce additional discretization errors due to their (i) typically lower orders of numerical approximation, (ii) anisotropic particle-mesh weighting. These errors lead to various numerical artefacts and non-conservation of particle momentum (“self-forces”). As a result, it is necessary to benchmark results from simulations obtained with curvilinear meshes with similar simulations performed with Cartesian meshes (Dyadechkin et al. 2013).

Some global hybrid codes employ adaptive mesh refinement (AMR) (Leclercq et al. 2016; Müller et al. 2011). Hybrid AMR simulations, however, may suffer from spurious particle “self-forces” and wave reflections that occur at the mesh refinement interfaces. To mitigate these artefacts, AMR algorithms are typically complemented with smoothing procedures, which, however, should be performed with caution in order to avoid affecting underlying physics in the regions of interest.

#### Temporal Scales

In addition to the “slow” Alfvénic (MHD) timescales, hybrid codes need to follow the “fast” ion kinetic, ion cyclotron and whistler timescales. This requirement typically makes global hybrid simulations numerically “stiff” in the near-Earth space, where timesteps, required for numerical accuracy and stability, may become prohibitively small (Omelchenko and Karimabadi 2012). To partially mitigate these effects, the kinetic ions may be replaced in this region by a dense fluid (Swift 1996; Lin et al. 2021). Hybrid-PIC simulations inherently generate spurious oscillations with large wave numbers, $k\sim 1/\Delta $ and high frequencies, $\omega \sim k^{2}\sim 1/\Delta ^{2}$. If not properly integrated or resistively damped, these noisy oscillations may explosively grow and abort simulation (Lin et al. 2008). Deleterious instabilities may be avoided by applying “noise filtering” (smoothing) or/and various “flux-limiting” techniques for electric and magnetic fields. These modifications, however, need to be implemented with caution, as the may produce artificial solutions not supported by the hybrid model.

For accuracy, typical full-orbit particle solvers (“pushers”) require that time steps, $\Delta t_{p}$ should be small enough that $\Omega \Delta t_{p} \ll 1$, where $\Omega $ is the local ion gyro-frequency. Using the same time step for all particles may create another numerical bottleneck in global hybrid simulations of the Earth’s magnetosphere. For instance, particle time steps of the order of $\Omega _{0}\Delta t_{p} \sim 0.05$ (where $\Omega _{0}$ is the ion gyro-frequency computed with respect to the IMF strength, $B_{IMF}$) fairly well describe ion gyro-motion in the solar wind (e.g. Turc et al. 2015; Guo et al. 2021b). At the same time, gyro-orbits and drifts of ions with $\Omega \gtrsim 10~\Omega _{0}$ (e.g. found in the cusp or some parts of the magnetosheath), will not be reproduced with accuracy. As a remedy, in some simulations, sub gyro-orbit time steps may be employed in these (large magnetic field) regions of the magnetosphere (Lin et al. 2014).

To summarize, predicting optimum global time steps for the particles and fields in global hybrid simulations is difficult in practice. This challenge has been addressed by replacing time stepping with an asynchronous approach to time integration, which combines discrete-event simulation (DES) with elements of artificial intelligence: Event-driven Multi-Agent Planning System (EMAPS) (Omelchenko and Karimabadi 2006b, 2022). EMAPS enables time advance of individual particles and local fields on meshes of arbitrary topology by integrating them on their self-adaptive timescales, similar to Conway’s Game of Life, where simulation elements evolve asynchronously based on a set of local interaction rules, rather than being updated synchronously at global time steps (Gardner 1970). Thus, EMAPS effectively performs the role of an intelligent “simulation time operating system”. This approach was first applied to model 1D collisionless plasma shocks (Omelchenko and Karimabadi 2006a) and fluids (Omelchenko and Karimabadi 2006b, 2007). Implemented in HYPERS (Omelchenko and Karimabadi 2012), EMAPS has enabled efficient and accurate global 3D hybrid simulations of the Earth’s magnetosphere (Omelchenko et al. 2021a,b, 2023).

Global 3D hybrid simulations of the Earth’s magnetosphere are typically performed for simulation periods, $\Omega _{0}t\sim 100-500$, where $\Omega _{0}$ is the IMF based proton cyclotron frequency. Assuming $B_{IMF}=5~nT$, these simulations formally span relatively short (compared to MHD) magnetospheric times, $t < 20\text{ min}$. For convenience, in order to present physical results in “magnetospheric hours”, some modelers multiply this simulation time by a model scaling factor (Lin et al. 2022b). Although this scaling is useful for comparing “macro-scale” simulation phenomena with observations, it is not appropriate for describing ion kinetic effects, e.g. those that drive the “magnetokinetic” formation of high-speed jets (Omelchenko et al. 2021a).

#### Plasmasphere and Ionosphere

Currently, hybrid codes cannot model global magnetospheric convection lasting many hours or days, e.g. a steady-state process of magnetotail loading and unloading. Therefore, global hybrid models typically assume a simple perfectly conducting or resistive ionosphere, where dipole magnetic field lines are “tied up” to the inner boundary (zero electric field) or allowed to diffuse due to its finite resistivity, respectively. model (e.g. Lin et al. 2021),

To avoid computing fast kinetic timescales, a cold, incompressible, dense ion fluid may be assumed to co-exist together with low-density particle ions in the inner magnetosphere within the distance of plasmasphere, where the plasma density is high (Swift 1996; Lin and Wang 2005; Lin et al. 2014). In ANGIE3D, this region is bounded by the near-Earth (inner) boundary, which is located at a radial distance at $r \simeq 3.5 \ R_{E}$ in the inner magnetosphere. The field-aligned currents, calculated near this inner boundary and mapped along the geomagnetic dipole field lines down to the ionospheric altitude (1000 km), are used as input to the ionospheric potential equation solved on a sphere (Lin et al. 2014, 2021):

50 $$ \nabla \cdot (-{\boldsymbol{\Sigma}} \cdot \nabla \Phi ) = J_{\parallel }sin I, $$

where $\Sigma $ is the conductance tensor, $\Phi $ is the electric potential, $J_{\parallel}$ is the mapped field-aligned current density, and $I$ is the inclination of the dipole field at the ionosphere. The static analytical model of Hall and Pederson conductance that accounts for EUV and diffuse auroral contributions can be used for the conductance tensor. Similarly to the global MHD models, the ionospheric electric field is mapped along the dipole lines back to the inner magnetospheric boundary, to serve as a boundary condition for the cold ion fluid (Lin et al. 2021).

### Hybrid Simulations with Inertial Electrons: Hybrid-PIC Code CHIEF

The MMS mission investigates physical processes like magnetic reconnection, shock waves and turbulence, which span from ion to electron scales. Simulation studies of these processes should ideally cover full kinetic physics from ion to electron scales for which the necessary present and near-future computational resources are prohibitively expensive. Therefore simulation models, which cover different scale ranges and physical phenomena, are used.

Hybrid-kinetic plasma simulation model, introduced in the previous Sect. 4.1, treats ions as kinetic species and electrons as a massless fluid. This restricts their applicability to physical processes in which not only electron kinetic effects are not important but also to the scales exceeding by far the electron scales. Hybrid-kinetic codes with inertia-less electrons, discussed in Sect. 4.1, can, therefore, be used to simulate global phenomena and in some cases for specifically limited physics studies of magnetic reconnection, plasma turbulence and shock waves.

The validity of hybrid-kinetic model can, however, be extended down to electron length scales, viz., to electron inertial length by considering electrons as an inertial fluid (Jain et al. 2023). Since the electron kinetic physics is still ignored such plasma model might computationally be more feasible than the fully kinetic model and describe larger scale phenomena and plasma process like magnetic reconnection, plasma turbulence and shock formation in collisionless plasmas, in which electron scale structures develop.

#### Model Equations: Inertial Electrons

Hybrid-kinetic model treats ions as kinetic species and electrons as an inertial fluid. In hybrid-kinetic simulation codes, ion dynamics can be described by solving either the ion’s Vlasov equation using Eulerian methods or the equations of motion for ion macro-particles using semi-Lagrangian Particle-in-Cell (PIC) method. Solving Vlasov equation is computationally more expensive. Here we discuss the hybrid-PIC codes which treat ions as Lagrangian macro-particles modelled via the PIC method. Following are the governing equations of hybrid-PIC model.

51 $$\begin{aligned} \frac{d\mathbf{x}_{i}}{dt} & =\mathbf{v}_{i}, \end{aligned}$$

52 $$\begin{aligned} m_{i}\frac{d\mathbf{v}_{i}}{dt} & =e(\mathbf{E} + \frac{\mathbf{v}_{i}\times \mathbf{B}}{c}), \end{aligned}$$

53 $$\begin{aligned} \nabla \times \mathbf{E} & = -\frac{1}{c} \frac{\partial \mathbf{B}}{\partial t}, \end{aligned}$$

54 $$\begin{aligned} \nabla \times \mathbf{B} & = \frac{4\pi}{c} \mathbf{J}, \end{aligned}$$

55 $$\begin{aligned} \mathbf{J} & = e (n_{i}\mathbf{u}_{i}-n_{e}\mathbf{u}_{e}), \end{aligned}$$

56 $$\begin{aligned} n_{i}&=n_{e} \end{aligned}$$

57 $$\begin{aligned} \mathbf{E} & = -\frac{\mathbf{u}_{e}\times \mathbf{B}}{c} - \frac{1}{en}\nabla p_{e} - \frac{m_{e}}{e}\left ( \frac{\partial \mathbf{u}_{e}}{\partial t}+(\mathbf{u}_{e}\cdot \mathbf{\nabla})\mathbf{u}_{e}\right ) + \eta \mathbf{J}, \end{aligned}$$

58 $$\begin{aligned} p_{e}&=C n_{e}^{\gamma} \end{aligned}$$

The electric and magnetic fields ($\mathbf{E}$ and $\mathbf{B}$ respectively) in Maxwell’s equations, Eqs. (53) and (54), are coupled to the plasma dynamics via the total current density $\mathbf{J}=e (n_{i}\mathbf{u}_{i}-n_{e}\mathbf{u}_{e})$ resulting from the bulk motion of ions (number density $n_{i}$, bulk velocity $\mathbf{u}_{i}$) and electrons (number density $n_{e}$, bulk velocity $\mathbf{u}_{e}$). Ion’s number density $n_{i}$ and the bulk velocity $\mathbf{u}_{i}$ is obtained from their positions $\mathbf{x}_{i}$ and velocities $\mathbf{v}_{i}$ governed by Eqs. (51) and (52). Electron dynamics is governed by quasi-neutrality condition, Eq. (56), momentum equation of the inertial electron fluid, Eq. (57), and equation of state relating electron scalar pressure $p_{e}$ with electron number density $n_{e}$, Eq. (58). Here, $e$ is the fundamental charge, $m_{i}$ ion mass, $m_{e}$ electron mass, $\eta $ collisional resistivity, $\gamma $ the adiabatic constant and $C$ is a constant (to be determined from initial conditions). Equations (51)-(58) are the fundamental equations of the hybrid-kinetic model with inertial electron fluid (Jain et al. 2023). These equations differ from the equations of hybrid-kinetic model with inertia-less electron fluid only by the electron inertial terms proportional to $m_{e}$ on the RHS of Eq. (57). Addition of electron inertial terms in Eq. (57) makes the numerical solution of these equations much more involved in comparison to the case of inertia-less electron fluid. The algebraic calculation of electric field from Eq. (57) is not as straightforward as in the case of the inertia-less electron fluid. One needs to now calculate time derivative of $\mathbf{u}_{e}$ or find some other way to obtain electric field. The calculation of magnetic field also now requires numerical solution of additional elliptic partial differential equations arising because of the finite electron inertia.

In majority of the hybrid-kinetic codes with inertial electrons, evolution of magnetic field is followed by solving an evolution equation for the generalized vorticity $\mathbf{W}=\nabla \times \mathbf{u}_{e}-e\mathbf{B}/m_{e} c$ obtained by taking curl of Eq. (57) and using Eq. (53). This equation is,

59 $$\begin{aligned} \frac{\partial \mathbf{W}}{\partial t} & = \mathbf{\nabla}\times \left [\mathbf{u}_{e}\times \mathbf{W}\right ]-\mathbf{\nabla}\times \left (\frac{\mathbf{\nabla} p_{e}}{m_{e}n}\right )- \mathbf{\nabla} \times \left (\frac{e \eta}{m_{e}}\mathbf{J}\right ). \end{aligned}$$

The magnetic field is then calculated by solving an elliptic partial differential equation (PDE) which is obtained by substituting for $\mathbf{u}_{e}$ from Eq. (54) and (55), $\mathbf{u}_{e}=\mathbf{u}_{i}-c\nabla \times \mathbf{B}/(4\pi e n)$, in the expression for $\mathbf{W}=\nabla \times \mathbf{u}_{e}-e\mathbf{B}/m_{e}c$.

60 $$\begin{aligned} \frac{c}{4 \pi e}\mathbf{\nabla}\times \left ( \frac{\mathbf{\nabla}\times \mathbf{B}}{n}\right )+ \frac{e\mathbf{B}}{m_{e}c} & = \mathbf{\nabla}\times \mathbf{u}_{i}- \mathbf{W}. \end{aligned}$$

Some of the hybrid-kinetic codes make approximations of electron inertial terms in Eq. (57) and (60) to simplify their numerical solutions (Lipatov 2002; Shay et al. 1998; Kuznetsova et al. 1998). Spatial density variations are neglected in Eq. (60) (Shay et al. 1998; Kuznetsova et al. 1998). The electric field was then calculated from the generalized Ohm’s law by neglecting the electron inertial term with time derivatives of the electron fluid velocity (Kuznetsova et al. 1998). These approximations are valid when length scale of variations is much larger than the electron inertial length. For a detailed discussion of these approximations, see Muñoz et al. (2018). These hybrid-kinetic codes which partially included electron inertial effects have mainly been used to study collisionless magnetic reconnection (Shay et al. 1999; Kuznetsova 2000; Kuznetsova et al. 2001). In particular Shay et al. (1998) used an evolution equation for a scalar electron pressure while Kuznetsova et al. (1998) included the full electron pressure tensor to take into account the non-gyrotropic effects.

More recently a hybrid-kinetic code CHIEF (Code Hybrid With Inertial Electron Fluid) was developed (Muñoz et al. 2018). This code solves Eqs. (57) and (60) without making any of the electron inertia related approximations used by other codes. The details of the numerical algorithm to solve Eqs. (51)-(58) are discussed by Muñoz et al. (2018). CHIEF was used to simulate kinetic plasma turbulence and it was found that the electron inertia related approximations are not valid in electron scale current sheets formed in the turbulence (Jain et al. 2022; Muñoz et al. 2023).

Some hybrid-kinetic codes with electron inertia calculate electric field from an elliptic PDE instead of Eq. (57) (Amano et al. 2014; Valentini et al. 2007). The elliptic PDE for the electric field is obtained by taking curl of Faraday’s law, Eq. (53), and utilizing Eqs. (54) and (57). Electron inertia effects were considered in the elliptic equation for the electric field while, still, the electron inertia term was ignored that contains the divergence of the electric field. Two dimensional simulations of kinetic plasma turbulence have shown that this approximation is not valid from ion to electron scales (Jain et al. 2022).

#### Hybrid-PIC Code CHIEF for Magnetic Reconnection Studies

Hybrid-PIC code CHIEF can be used to study magnetic reconnection with a large guide magnetic field in which case electron non-gyrotropy is weak and reconnection electric field is expected to be balanced by the electron inertial terms in generalized Ohm’s law. Indeed, PIC simulations of magnetic reconnection have shown that electron inertial terms are significant to balance the reconnection electric field when guide field is large (Hesse and Winske 1998; Hesse et al. 2016; Liu et al. 2013; Pritchett 2005).

A particular reconnection study for which CHIEF code can be employed is electron-only reconnection in which ions do not couple to electrons (Sharma Pyakurel et al. 2019), examples of which are discussed in Sect. 3.7.2. Electron-only reconnection has recently been discovered in space observations (Phan et al. 2018). In many of electron only reconnection events observed by MMS, guide magnetic field is significantly larger than the asymptotic value of the reconnecting component magnetic field (Phan et al. 2018; Zhou et al. 2021; Man et al. 2020; Stawarz et al. 2022). In the statistical survey of electron only reconnection events in Earth’s magnetosheath, guide field in majority of the events is 1 to 10 times larger than the reconnecting component of the magnetic field (Stawarz et al. 2022). MMS observations of electron scale reconnection in Earth’s magnetosheath show that the electron non-gyrotropy, which balances the reconnection electric field in weak or zero guide field case, reduces with the increasing strength of the guide magnetic field (Wilder et al. 2018). No evidence of agyrotropy was found in another MMS observations of large guide field reconnection (Eriksson et al. 2016). For the electron only events observed with large guide magnetic field and/or absence of non-gyrotropy, electrons can be modeled as an inertial electron fluid. Hybrid-PIC code CHIEF treats electrons as inertial fluid without any approximations and can be used to study electron-only reconnection.

#### Outlook

Hybrid-kinetic simulations with electron inertia provide a computationally less expensive (in comparison to fully kinetic simulations) tool to study magnetic reconnection with guide field in which bulk electron inertia is the dominant mechanism breaking the frozen-in condition of magnetic field. There have been some hybrid-kinetic simulations studies with electron inertia of guide field magnetic reconnection (Kuznetsova et al. 1998; Kuznetsova 2000; Shay et al. 1998; Califano et al. 2020; Muñoz et al. 2023). More studies are, however, required to address still many open questions about the guide field magnetic reconnection. The EMHD limit (stationary ions) of the hybrid-kinetic model with electron inertia is particularly useful to study the nature of reconnection in electron scale current sheets and is relevant for the recently discovered electron-only reconnection (Phan et al. 2018). At the same time, simulations of kinetic plasma turbulence from ion to electron scales using hybrid-kinetic model with electron inertia will shed light on the conditions under which electron scale current sheets form and reconnect with or without ion coupling in the turbulence.

## Fully Kinetic Particle-in-Cell Simulations

### Introduction

**Fig. 6:**
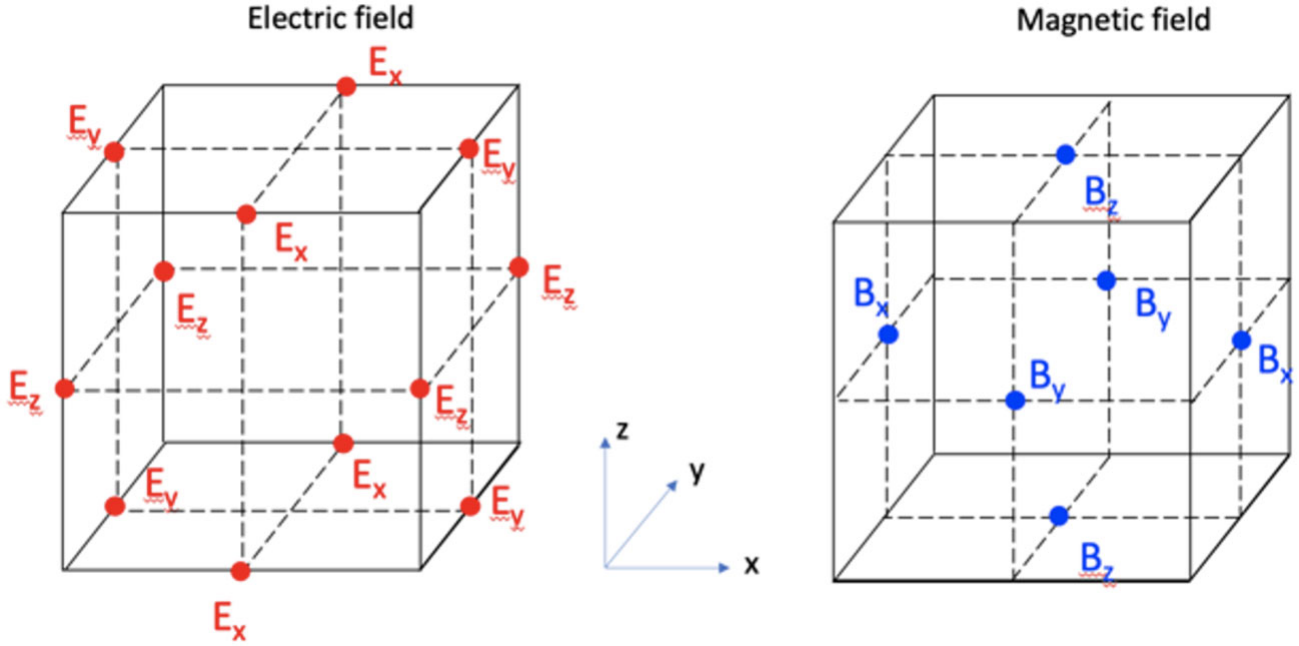
Yee lattice and electric and magnetic fields in a cell in a 3D case, where the length of each side is $\Delta x$. Electric fields $\boldsymbol{E}$ are defined at the midpoint of each side of the cube, while magnetic fields $\boldsymbol{B}$ are defined at the center of each face of the cube. In a 2D case, all the quantities are defined in the $x$-$y$ plane, projecting each position onto the cell in the $x$-$y$ plane

The particle-in-cell (PIC) method is a simulation method in which the plasma is treated as a collection of particles (electrons and ions), where each species is typically composed of up to $10^{12}$ particles in 3D cases. In PIC simulations, the motions of individual particles and the evolution of electric and magnetic fields are solved self-consistently. The electric and magnetic fields are defined on discrete grid points. In this subsection, we will explain an explicit PIC simulation, where all the quantities are updated based on the quantities obtained in the previous time step. Let us assume that the total particle number in a simulation is $N_{p}$ (in other words, $N_{p}/2$ for ions, and $N_{p}/2$ for electrons). The equation of motion for the $j$-th particle’s position $\boldsymbol{x}_{j}(t)$ and momentum $\boldsymbol{p}_{j}(t)$, where $j$ represents an integer between 1 to $N_{p}$, is discretized in time, while Maxwell’s equations for electromagnetic fields $\boldsymbol{E}(\boldsymbol{x},t)$ and $\boldsymbol{B}(\boldsymbol{x},t)$ are discretized in both space and time, using a grid spacing $\Delta x$ (assuming that the grids are uniform in all the coordinates, i.e. $\Delta x=\Delta y= \Delta z$) and a time step $\Delta t$, respectively. They are given as

61 $$ \frac{\boldsymbol{x}_{j}(t_{n})-\boldsymbol{x}_{j}(t_{n-1})}{\Delta t}= \frac{\boldsymbol{p}_{j}(t_{n-1/2})}{m_{j}\gamma _{j}(t_{n-1/2})}, $$

62 $$ \frac{\boldsymbol{p}_{j}(t_{n+1/2})-\boldsymbol{p}_{j}(t_{n-1/2})}{\Delta t}=q_{j} \left [\boldsymbol{E}(\boldsymbol{x}_{j}(t_{n}),t_{n})+ \frac{\boldsymbol{p}_{j}(t_{n})}{cm_{j}\gamma _{j}(t_{n})} \times \boldsymbol{B}(\boldsymbol{x}_{j}(t_{n}),t_{n}) \right ], $$

63 $$ \frac{\boldsymbol{E}(\boldsymbol{x},t_{n+1})-\boldsymbol{E}(\boldsymbol{x},t_{n})}{\Delta t}=-4 \pi \boldsymbol{J}(\boldsymbol{x},t_{n+1/2})+c\nabla _{f} \times \boldsymbol{B}(\boldsymbol{x},t_{n+1/2}), $$

64 $$ \frac{\boldsymbol{B}(\boldsymbol{x},t_{n+3/2})-\boldsymbol{B}(\boldsymbol{x},t_{n+1/2})}{\Delta t}=-c \nabla _{f}\times \boldsymbol{E}(\boldsymbol{x},t_{n+1}), $$

where $m_{j}$ is a mass, $\gamma _{j}$ is the Lorentz factor, $q_{j}$ is a charge, $c$ is the speed of light, and the operator $\nabla _{f} \times $ represents the finite difference version of the curl operation. The time is discretized to be $t_{a}=a \Delta t$, where $a$ represents an integer $n$ or a half integer $n+1/2$. Field quantities such as $\boldsymbol{B(x},t_{n})$ are defined at a grid position $\boldsymbol{x}$, while quantities such as $\boldsymbol{B(x}_{j}(t_{n}), t_{n})$ are defined at the position of a particle $\boldsymbol{x}(t_{n})$. Note that $\boldsymbol{B}(\boldsymbol{x}_{j}(t_{n}), t_{n})$ in the right-hand side of Eq. (62) represents the mean of $\boldsymbol{B}(\boldsymbol{x}_{j}(t_{n}), t_{n+1/2})$ and $\boldsymbol{B}(\boldsymbol{x}_{j}(t_{n}), t_{n-1/2})$. As seen in Eqs. (61) and (62), the position $\boldsymbol{x}_{j}$ is computed at integer time, $t=t_{n}$, while the momentum $\boldsymbol{p}_{j}$ is computed at half-integer time, $t=t_{n+1/2}$. This time staggering gives the second-order accuracy, i.e. the error is $O(\Delta t^{2})$. In the same way, for the spatial discretization for $\boldsymbol{E}(\boldsymbol{x},t)$ and $\boldsymbol{B}(\boldsymbol{x},t)$, the Yee lattice (Yee 1966) is used, in which each component of electric and magnetic fields is defined as in Fig. 6. Also, each component of the current density $\boldsymbol{J}(\boldsymbol{x},t)$ is defined at the same position as $\boldsymbol{E}(\boldsymbol{x},t)$. These fields, which are spatially and temporarily staggered, are advanced using Eqs. (63) and (64), keeping the second-order accuracy in space and time. Equation (64) guarantees that the Gauss’s law for the magnetic field, $\nabla _{f}\cdot \boldsymbol{B}=0$, where $\nabla _{f} \cdot $ represents the finite difference version of the divergence, is satisfied when it is satisfied at $t=0$.

To solve Eq. (62), the Boris method (Boris 1970) is commonly used. This method has three steps: (1) The momentum is updated from $\boldsymbol{p}_{j}(t_{n-1/2})$ to $\boldsymbol{p}_{j}(t_{n})^{*}$, using only the electric field $\boldsymbol{E}(\boldsymbol{x}_{j}(t_{n}),t_{n})$ for a half time step $\Delta t/2$. (2) The momentum vector $\boldsymbol{p}_{j}(t_{n})^{*}$ is rotated to be $\boldsymbol{p}_{j}(t_{n})^{**}$ using only the magnetic field $\boldsymbol{B}(\boldsymbol{x}_{j}(t_{n}),t_{n})$ for a full time step $\Delta t$. (3) The rotated momentum is further updated from $\boldsymbol{p}_{j}(t_{n})^{**}$ to $\boldsymbol{p}_{j}(t_{n+1/2})$, using the electric field $\boldsymbol{E}(\boldsymbol{x}_{j}(t_{n}), t_{n})$ for another half time step $\Delta t/2$.

In the PIC method, each particle is not a point particle, but it has a finite size to reduce noise. The shape of a particle depends on simulation codes, but the most-commonly used shape function is a triangular function, $S_{x}(x-x_{j})=(1-\mid x-x_{j}\mid / \Delta x)$ when $|x-x_{j}|/\Delta x<1$ and zero otherwise, where only the $x$ component is considered. In 2D and 3D simulations, the $y$ and $z$ components of the shape functions are multiplied, as $S(\boldsymbol{x}-\boldsymbol{x}_{j})=S_{x}(x-x_{j})S_{y}(y-y_{j})$ for 2D and $S(\boldsymbol{x}-\boldsymbol{x}_{j})=S_{x}(x-x_{j})S_{y}(y-y_{j})S_{z}(z-z_{j})$ for 3D. Using these shape functions, the charge density $\rho (\boldsymbol{x},t)$ is computed as $\rho (\boldsymbol{x},t)=\sum _{j}q_{j}S(\boldsymbol{x}- \boldsymbol{x}_{j})$. This way of charge assignment is reversed to compute the electric field exerted from each grid point to a particle’s position. To avoid the self-force (the force due to the electric field generated by the particle itself), we must first average the electric fields defined on half-integer grids to obtain the mean electric field on each integer grid, before assigning the electric fields to the particle. The magnetic fields are assigned from grids to the particle position in the same way.

The current density can also be calculated using $\boldsymbol{J}(\boldsymbol{x},t)=\sum _{j}q_{j} \boldsymbol{v}_{j}S(\boldsymbol{x}-\boldsymbol{x}_{j})$, where $\boldsymbol{v}_{j}$ is the velocity, but the calculated $\boldsymbol{J}(\boldsymbol{x},t)$ does not satisfy the continuum equation, $[\rho (\boldsymbol{x},t_{n+1})-\rho (\boldsymbol{x},t_{n})]/ \Delta t+\nabla _{f} \cdot \boldsymbol{J}(\boldsymbol{x},t_{n+1/2})=0$; therefore, the electric field calculated using Eq. (63) with this $\boldsymbol{J}(\boldsymbol{x},t)$ does not satisfy the Gauss’s law, $\nabla _{f} \cdot \boldsymbol{E}(\boldsymbol{x},t_{n+1})=4 \pi \rho (\boldsymbol{x},t_{n+1})$. This means that we must either correct the electric field $\boldsymbol{E}(\boldsymbol{x},t_{n+1})$ to satisfy the Gauss’s law, or use another method to compute $\boldsymbol{J}(\boldsymbol{x},t)$. A technique for the former is explained in Birdsall and Langdon (1991). For the latter, for example, Villasenor and Bunemann (1992) developed a rigorous charge conservation method for 2D and 3D PIC simulations, which guarantees that both the Gauss’s law and the continuum equation are satisfied at the same time, when they are satisfied at $t=0$.

The time step $\Delta t$ must satisfy the Courant–Friedrichs–Lewy condition, $\Delta t<\Delta x/ (cN^{1/2}_{d})$, where $N_{d}$ represents the dimensionality ($N_{d}=1, 2$, or 3). Also, the grid spacing $\Delta x$ should be close to the Debye length $\lambda _{D}$, otherwise strong numerical heating occurs. Even when those conditions are satisfied, if particles are relativistic, a numerical Cherenkov instability can occur and the noise field becomes extremely large. When this occurs, a noise reduction method such as by Godfrey (1980) can be used.

To study magnetic reconnection, it is important to separate the spatiotemporal scale of protons and that of electrons, which is controlled by the ion to electron mass ratio, $m_{i}/m_{e}$. In general, to reduce the computing time, full PIC simulations use an artificial mass ratio, such as $m_{i}/m_{e}=25$ and 100, which is smaller than the real mass ratio $m_{i}/m_{e}=1836$. There are two effects if we use a smaller mass ratio. One is that the thickness of the electron-scale current layer near the reconnection X-line, which is of the order of the electron skin depth $d_{e}=c/\omega _{pe}$, becomes thicker than that for a realistic case. The other is that the time scale of the electron physics becomes longer than the realistic case. For example, if we choose the mass ratio 25, the spatial scale separation between the ion scale ($d_{i}$) and the electron scale ($d_{e}$) is 5 times, and the temporal scale separation between the ion scale ($\Omega _{i}^{-1}$) and the electron scale ($\Omega _{e}^{-1}$) is 25 times. Even though these are much smaller than those in the realistic case, the choice of the mass ratio 25 is acceptable in order to see the scale separation physics that occurs in a study of magnetic reconnection.

Various boundary conditions can be implemented including periodic, conducting wall, and open boundaries (Daughton and Scudder 2006; Ohtani and Horiuchi 2009). For open boundaries, particles that reach the boundaries are removed, and new particles are injected into the simulation box at each time. How to inject particles depends on simulation codes. For example, in Daughton and Scudder (2006), the flux of injecting particles is calculated assuming that the spatial derivative of the distribution function at the boundary is zero in the normal direction. Also, electromagnetic fluctuations can pass through the boundaries.

Some research groups use PIC codes with the adaptive mech refinement (AMR) technique (Fujimoto 2011; Innocenti et al. 2013). In AMR PIC simulations, the simulation region is subdivided based on the required spatial resolution: the regions where the small-scale physics becomes important are solved using fine grids, while the other regions outside the fine regions are solved using coarse grids. If more smaller-scale resolution is required, further finer levels of grids are produced. This technique can reduce the required particle number for fully kinetic simulations.

### Magnetotail Reconnection

In this section, we introduce simulation results of magnetic reconnection obtained by the standard fully kinetic PIC simulations with uniform grids. In the Earth’s magnetotail, the strength of magnetic field across the current sheet is symmetric, and the guide field ($B_{y}$ field in the GSM coordinates) is small in many reconnection events. Many authors have been studying symmetric magnetic reconnection with zero guide-field, using the Earth’s magnetotail parameters (Hoshino 1987; Pritchett et al. 1991; Horiuchi and Sato 1994; Dreher et al. 1996; Zhu and Winglee 1996; Hesse et al. 1996). In these simulations, the initial plasma is set up based on a Harris equilibrium (Harris 1962a): the magnetic field $B_{x}=B_{0}\text{tanh}(z/w)$, and the density $n=n_{0}\text{sech}^{2}(z/w)+n_{b}$, where $B_{0}$ is the asymptotic magnetic field, $w$ is the sheet thickness, $n_{0}$ is the peak density of the current sheet, and $n_{b}$ is the background density. Also the conditions for the Harris equilibrium, $B_{0}^{2}/(8\pi )=n_{0}(T_{i}+T_{e})$ and $|V_{di}-V_{de}|=[2c/(weB_{0})](T_{i}+T_{e})$, and $V_{di}/V_{de} =- T_{i} /T_{e}$, are satisfied, where $T_{i}$ and $T_{e}$ are the ion and electron temperatures, respectively, $e$ is the elementary charge, and $V_{di}$ and $V_{de}$ are the $y$-directional drift velocity in the current sheet component of ions and electrons, respectively.

The following describes an example of a 2D PIC simulation of magnetotail reconnection, Hesse et al. (2018a). The system size is $L_{x}\times L_{z}=102.4d_{i} \times 51.2d_{i}$, where $d_{i}$ is the ion skin depth, $c/\omega _{pi}$ with $\omega _{pi}$ being the ion plasma frequency based on $n_{0}$ ($\omega _{pi}=(4\pi n_{0}e^{2}/m_{i})^{1/2}$), and 3200 × 3200 grids are used. The mass ratio is $m_{i}/m_{e}=100$, the sheet thickness is $w=0.5d_{i}$, the temperature ratio is $T_{i}/T_{e} =5$, the density ratio $n_{b}/n_{0}=0.2$, and the ratio of the plasma frequency (based on $n_{0}$) to the electron cyclotron frequency (based on $B_{0}$) is $\omega _{pe}/\Omega _{e}=(4\pi n_{0}e^{2}/m_{e})^{1/2}/[eB_{0}/(m_{e}c)]=2.0$, which gives the ratio of the light speed to the Alfvén speed (based on $B_{0}$ and $n_{0}$, $v_{A0}=B_{0}/(4\pi m_{i}n_{0})^{1/2}$) to be $c/v_{A0}=20.0$. The $x$ boundaries are periodic, and the $z$ boundaries are conducting walls. To initiate magnetic reconnection, a perturbation is added to the magnetic field as $\delta B_{x}=(a_{0}\pi /L_{z})\cos (2\pi x/L_{x})\sin (\pi z/L_{z})$ and $\delta B_{z}=-(a_{0}2\pi /L_{x})\sin (2\pi x/L_{x})\cos (\pi z/L_{z})$, which gives a reconnection X-line at the origin $x=0$ and $z=0$. Here $a_{0}$ is the amplitude of the perturbation. The total number of particles used in the simulation is $7 \times 10^{10}$.

**Fig. 7:**
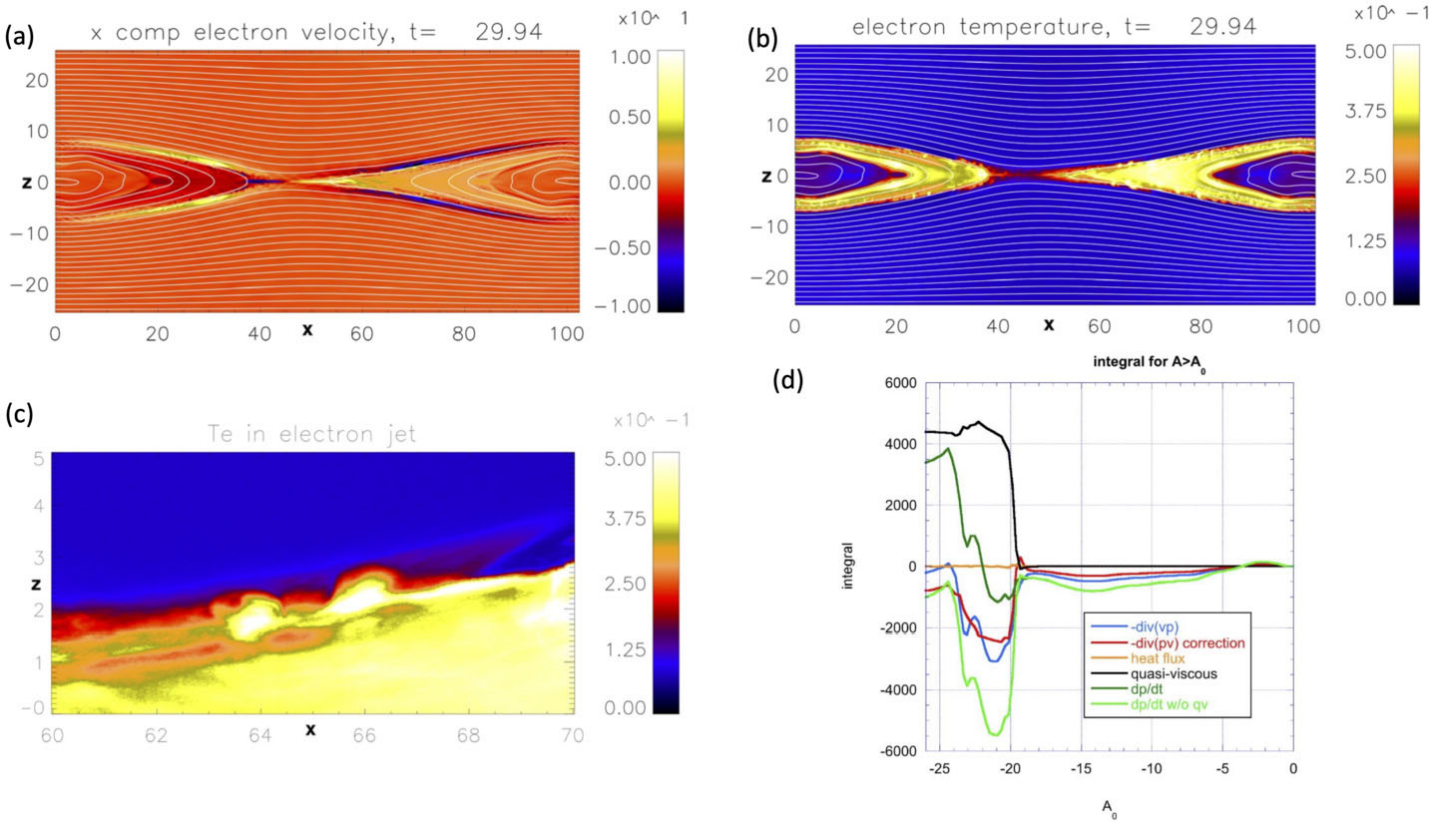
2D PIC simulation result for magnetotail reconnection. (a) Electron fluid velocity $V_{ex}$, (b) electron temperature $T_{e}$, (c) zoom-in view of $T_{e}$, and (d) the heating term in Eq. (65). Adapted from Hesse et al. (2018a, 2019)

After the simulation starts, the reconnection electric field $E_{y}$ is generated in the diffusion region near the X-line, where both the ion and electron motions are decoupled from the magnetic field line motion, which allows a pair of magnetic field lines across the current sheet, one is in the positive $z$ region ($B_{x}>0$) and the other is in the negative $z$ direction ($B_{x}<0$), are reconnected and energy conversion occurs from the magnetic energy to the kinetic and thermal energies of ions and electrons. The reconnection rate is measured as $E_{y}/(B_{0}v_{A0}/c)$, and in this simulation it is around 0.2 (Hesse et al. 2018b). Both ion and electron outflows are produced from the X-line toward the positive and negative $x$ directions. Figure 7(a) shows the electron fluid velocity $V_{ex}$, where the bipolar positive and negative $V_{ex}$ peaks appear along the $z=0$ in $40< x/d_{i}<60$, and each peak value reaches near the electron Alfvén speed, $v_{Ae}=B_{0}/(4\pi m_{e}n_{0})^{1/2}=(m_{i}/m_{e})^{1/2}v_{A0}$. Outside the region of $40< x/d_{i}<60$, there are strong inflows, which also reach near $v_{Ae}$, toward the X-line along the separatrices. Because of these strong counter streaming electron flows (outflows and inflows), an electrostatic instability occurs that produces waves propagating along the separatrices toward the X-line, and electrons are heated due to wave-particle interactions. Figure 7(b) and (c) show the electron temperature in the entire box and a zoom-in view that includes a separatrix. Electrons are heated inside the separatrices (panel (b)), and the zoom-in view (panel (c)) shows that there are two solitary structures due to the nonlinear evolution of the wave (at $x=64d_{i}$ and $66d_{i}$ along the separatrix) where electron temperature significantly enhances.

The locations of the instability, along the separatrices, correspond to the boundary of the high electron temperature, which suggests the importance of the electrostatic instability to heat electrons. To understand the effect of the instability on the heating, Hesse et al. (2018a) analyzed the pressure equation:

65 $$ \frac{\partial p}{\partial t}=-\nabla \cdot (\boldsymbol{V}p)- \frac{2}{3}\sum _{l}P_{ll}\frac{\partial}{\partial x_{l}}V_{l}- \frac{1}{3}\sum _{l,i}\frac{\partial}{\partial x_{i}}Q_{lii}- \frac{2}{3}\sum _{l,i (l\neq i)}P_{li}\frac{\partial}{\partial x_{i}}V_{l}, $$

where all the quantities are for electrons (the subscript $e$ is omitted): $p$ is the scalar pressure, $\boldsymbol{V}$ is the fluid velocity, and $P_{ij}$ and $Q_{ijk}$ are the pressure tensor and the heat tensor, respectively. The first two terms represent the compression effect, the third term is due to the heat flux, and the fourth term represents the quasi-viscous effect due to the off-diagonal components of the pressure tensor. Figure 7(d) shows the contribution of each term in Eq. (65), integrated over the region of $A>A_{0}$, where $A$ is the $y$ component of the mangnetic flux function (i.e., $B_{x}=\partial A/\partial z$ and $B_{z}=-\partial A/\partial x$). Note that $A=0$ at the outermost $z$ boundaries, and $A$ is decreasing as we approach $z=0$. Figure 7(d) indicates that the quasi-viscous term (the fourth term) is the dominat term to provide the pressure increase, leading heating in the reconnection region.

### Magnetopause Reconnection

PIC simulations of magnetopause reconnection can include certain challenges due to asymmetries in the densities, temperatures and magnetic field strengths of the abutting plasmas (Sonnerup et al. 1986; Cassak and Shay 2007). Specifically, magnetospheric plasma is usually relatively sparse, hot, and threaded by a strong magnetic field, while magnetosheath plasma (which arises from shocked solar wind) is denser, cooler, and includes a somewhat weaker field.

From a simulation perspective, the density asymmetry – which can exceed an order of magnitude – can be particularly problematic. PIC simulations are inherently noisy. The random fluctuations tend to follow Poissonian statistics with an amplitude scaling as $1/\sqrt{N_{pc}}$, with $N_{pc}$ the number of (macro) particles per computational cell. If variations in the number of macroparticles directly translate to variations in density, a $16:1$ ratio between the magneosheath and magnetospheric plasma densities will produce noise levels an unacceptable four times larger in the latter than the former. One obvious approach – throwing more particles at the problem – can quickly become computationally burdensome. An alternative is the use of particle weighting, in which each particle is assigned a weight that determines its significance in the calculation of particle moments (e.g., charge and current density). Doing so allows for an initially uniform distribution of particles with a roughly constant noise level. More sophisticated algorithms allow for the splitting and joining of particles as the simulation progresses to account for the development of density variations and to address computational load imbalances.

A 2D simulation of the magnetopause with p3d, a PIC code employing weighted particles (Zeiler et al. 2002), was presented in Swisdak et al. (2018). In its normalization, a reference magnetic field strength $B_{0}$ and density $n_{0}$ define the velocity unit $v_{A0}=B_{0}/(4\pi m_{i}n_{0})^{1/2}$. Times are normalized to the inverse ion cyclotron frequency $\Omega _{i0}^{-1}=m_{i}c/(eB_{0})$, lengths to the ion inertial length $d_{i0} =c/\omega _{pi0}$ (where $\omega _{pi0} = (4\pi n_{0} e^{2}/m_{i})^{1/2}$ is the ion plasma frequency), electric fields to $v_{A0}B_{0}/c$, and temperatures to $m_{i}v_{A0}^{2}$.

The initial conditions closely mimic those observed during the diffusion region encounter described in Burch et al. (2016). In the system considered here, $B_{0}$ and $n_{0}$ correspond to their asymptotic magnetosheath values: $B_{0} = 23\text{ nT}$ and $n_{0} = 11.3\text{ cm}^{-3}$. The simulation uses an $LMN$ coordinate system in which the reconnecting field parallels the $L$ axis (roughly north-south), the $M$ axis runs roughly east-west, with dawnward positive, and the $N$ axis points radially away from Earth and completes the right-handed triad. The computational domain has dimensions $(L_{L},L_{N}) = (40.96,20.48)$ with periodic boundary conditions used in all directions. While particles can move in the $M$ direction, variations in physical quantities are not permitted: $\partial /\partial M = 0$.

The reconnecting component of the field $B_{L}$ and the ion and electron temperatures, $T_{i}$ and $T_{e}$, vary as functions of $N$ with hyperbolic tangent profiles of width 1. The asymptotic values of $n$, $B_{L}$, $T_{i}$, and $T_{e}$ in code units are 1.0, 1.0, 1.37, and 0.12 in the magnetosheath and 0.06, 1.70, 7.73, and 1.28 in the magnetosphere. Pressure balance determines the initial density profile. The guide field $B_{M}=0.099$ is much smaller than $B_{L}$ (i.e., the reconnection is nearly anti-parallel) and initially uniform. While not an exact kinetic equilibrium, the unperturbed configuration is in force balance and would not undergo significant evolution during the timescales of interest. Instead, a small initial perturbation is introduced to trigger reconnection onset.

The ion-to-electron mass ratio is chosen to be 100, which is sufficient to separate the electron and ion scales (the electron inertial length $d_{e0} = 0.1d_{i0}$). The normalized speed of light is $c=15$ so that $\omega _{pe}/\Omega _{e}=1.5$ in the asymptotic magnetosheath and $\approx 0.2$ in the asymptotic magnetosphere; the observed ratios are larger, $\approx 46$ and 7, and as a consequence the simulation’s Debye length is larger than in the real system. However, since the development of reconnection does not appreciably depend on physical effects at the Debye scale the expected impact is minimal. The spatial grid has resolution $\Delta = 0.01$ in normalized units while the Debye length in the simulation’s magnetosheath, $\approx 0.03$, is the smallest physical scale. To ameliorate numerical noise, particularly in the low-density magnetosphere, each grid cell initially contains 3000 weighted macroparticles.

**Fig. 8:**
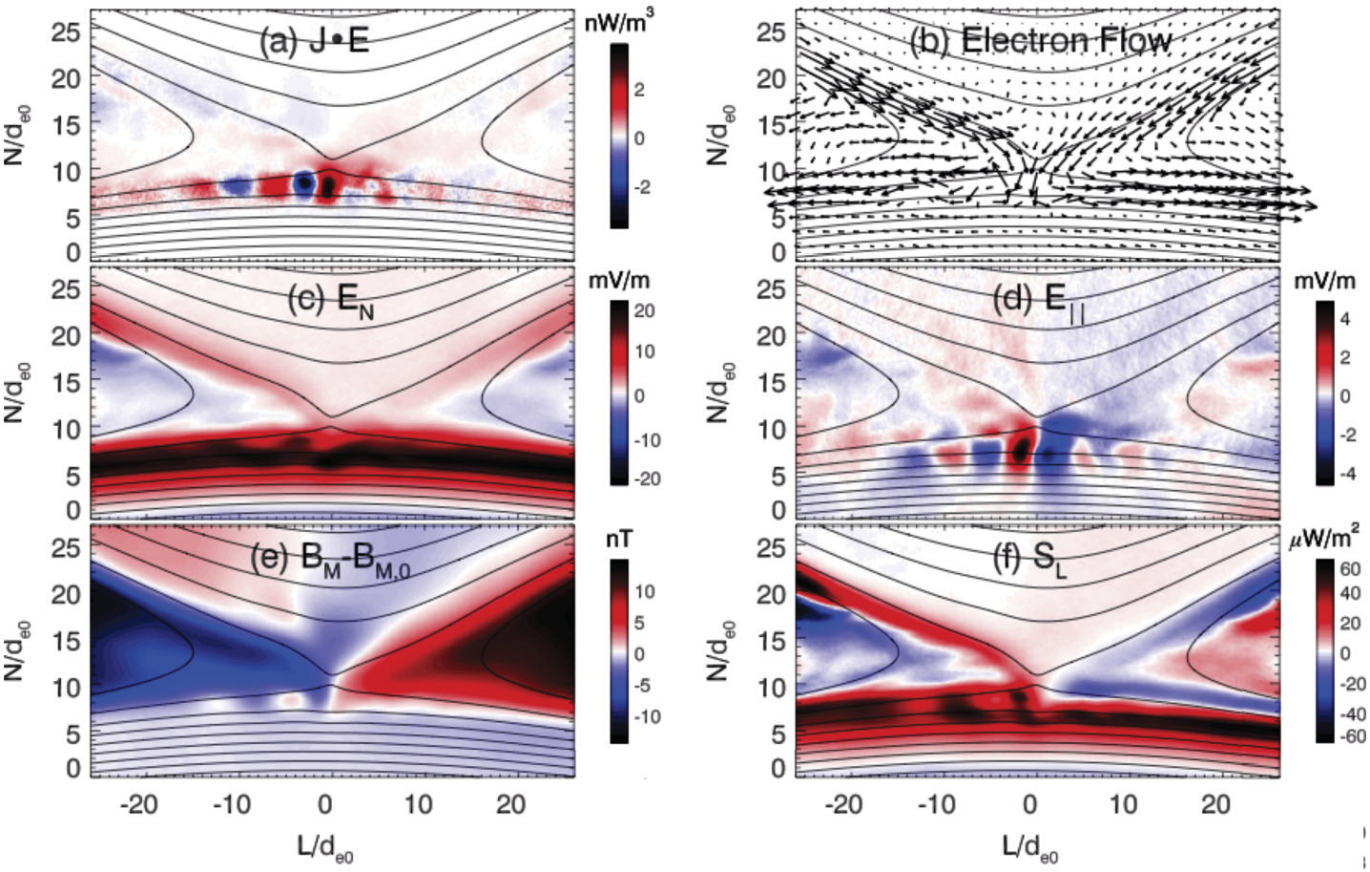
Simulation results overplotted with magnetic field lines. (a) The $\mathbf{J}\boldsymbol{\cdot}\mathbf{E}$ term from Poynting’s theorem; (b) In-plane electron flow field; (c) $E_{N}$, the normal component of the electric field; (d) $E_{\parallel}$, the component of the electric field parallel to the magnetic field; (e) $B_{M}-B_{M,0}$, the change in the out-of-plane component of the magnetic field from its (spatially constant) initial value; (f) $S_{L}$, the horizontal component of the Poynting flux. Adapted from Swisdak et al. (2018)

Figure 8 shows results. The asymmetry in the field strength is apparent in the distribution of the field lines, with the separatrices extending much farther (in the $N$ direction) into the plasma of the magnetosheath (top) than the magnetosphere (bottom). The Hall electric and magnetic fields (panels c and e) differ significantly from the case of symmetric reconnection, with the former concentrated almost exclusively on the magnetospheric side while the latter is almost completely dipolar rather than quadrupolar. Due to the use of weighted particles, the numerical noise is similar on both sides.

### The 3D Nature of Magnetic Reconnection

The third dimension pointing out of the 2D reconnection plane introduces numerous plasma instabilities (e.g. Daughton et al. 2011; Graham et al. 2025). In addition to these secondary instabilities, inherent 3D nature of reconnection X-line is also omitted in 2D pictures, which includes the effect of limited X-line extent (Shay et al. 2003; Liu et al. 2019; Huang et al. 2020; Pyakurel et al. 2021; Huang et al. 2024), its tendency of spreading (Huba 2003; Shay et al. 2003; Lapenta et al. 2006; Nakamura et al. 2012; Shepherd and Cassak 2012; Li et al. 2020; Arencibia et al. 2023; Li et al. 2023c; Lin et al. 2025), and its orientation preference (Sonnerup 1974; Swisdak and Drake 2007; Hesse et al. 2013; Aunai et al. 2016; Liu et al. 2013, 2018).

We briefly highlight the property of reconnection X-lines with a short extent here. To sustain a current sheet, electrons and ions drift in opposite directions. This fact introduces the asymmetry along the X-line (current) direction. To reveal this effect, Liu et al. (2019) and Huang et al. (2020) studied magnetic reconnection with the X-line spatially confined in the current direction. They included thick current layers to prevent the reconnection from spreading out of the two ends of a thin current sheet that has a thickness on an ion inertial ($d_{i}$) scale. The $x$ component of the magnetic field is given as $B_{x}=B_{0}\text{tanh}[z/L(y)]$, where the half-thickness $L(y)=L_{min}+(L_{max}-L_{min})[1-f(y)]$ and $f(y)=[\text{tanh}((y+w_{0})/S)-\text{tanh}((y-w_{0})/S)]/[2\text{tanh}(w_{0}/S)]$, $L_{min}=0.5d_{i}$, $L_{max}=4d_{i}$, and $S=5d_{i}$. The parameter $w_{0}$, which controls the $y$-extent of the thin current region ($L_{y-thin}$), is varied from $w_{0}=2d_{i}$ to $20d_{i}$, corresponding to the $y$-extent of the thin current region from $L_{y-thin}\sim 4d_{i}$ ($w_{0}=2d_{i}$) to $L_{y-thin}\sim 30d_{i}$ ($w_{0}=20d_{i}$). The density is $n=n_{0}\text{sech}^{2}[z/L(y)]+n_{b}$, and $n_{b}=0.3n_{0}$. The size of the system is $L_{x}\times L_{y}\times L_{z}=32d_{i}\times 64d_{i}\times 16d_{i}$. The periodic boundary condition is used for $x$ and $y$, and the conducting walls are placed in the $z$ boundaries. Over $2.6\times 10^{10}$ particles for each species are used.

**Fig. 9:**
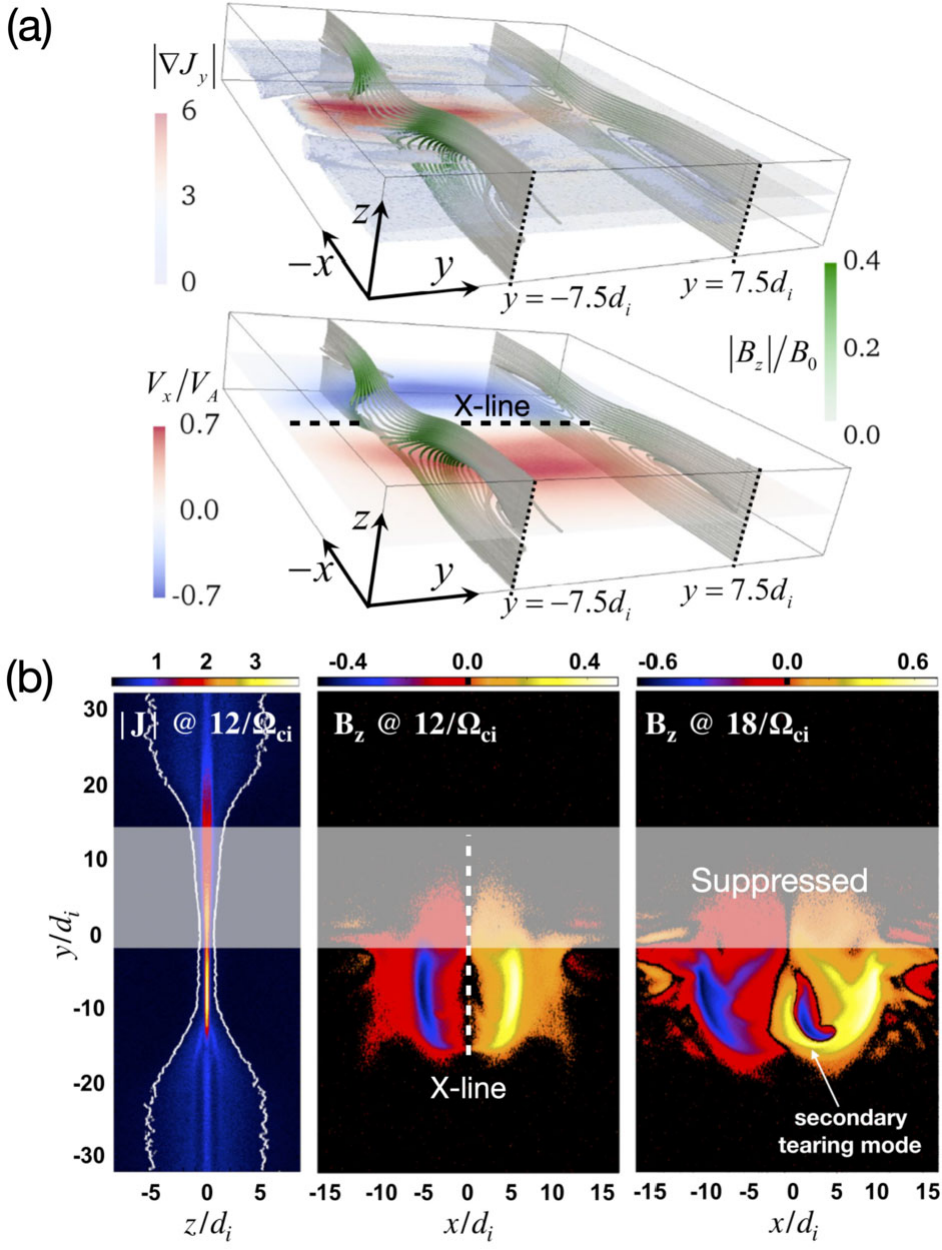
3D PIC simulation results. (a) 3D view of reconnection with a limited X-line extent, where the thin current sheet region extends $30d_{i}$ in $y$ (Huang et al. 2020). The mass ratio in the simulation is 25. (b) The current density on the $x=0$ plane (left) and magnetic field $B_{z}$ on the $z=0$ plane (middle and right) (Liu et al. 2019). The mass ratio is 75. The gray shaded area represents the “suppressed reconnecting region”. Adapted from Huang et al. (2020) and Liu et al. (2019)

The resulting reconnection is shown in Fig. 9, which is for $L_{y-thin}\sim 30d_{i}$. Liu et al. (2019) found that the reconnection rate and the outflow speed drop significantly when the extent of the thin current sheet, $L_{y-thin}$, is less than $\mathcal{O}(10d_{i})$. When the thin current sheet extent is long enough, it consists of two distinct regions: a suppressed reconnecting region (on the ion-drifting side, marked in Fig. 9(b)) exists adjacent to the active region where reconnection proceeds normally as in a 2D case with a typical fast rate value $\approx 0.1$. The extent of this suppression region is $\mathcal{O}(10d_{i})$, and it suppresses reconnection when $L_{y-thin}$ is comparable or shorter. The time scale of current sheet thinning toward fast reconnection can be translated into the spatial scale of this suppression region, because the electron drifts inside the ion diffusion region transport the reconnected magnetic flux (that is critical in driving outflows and furthers the current sheet thinning) away from this region. This is a consequence of the Hall effect in 3D.

Huang et al. (2020) incorporated the length scale of this suppression region $\mathcal{O}(10d_{i})$ to quantitatively model the reduction of the reconnection rate and the maximum outflow speed observed in the short X-line limit. The average reconnection rate drops because of the limited active region (where the current sheet thins down to the electron inertial scale) within the X-line. The outflow speed reduction correlates with the decrease of the $J\times B$ force, which can be modeled by the phase shift between the $J$ and $B$ profiles, also as a consequence of the flux transport out of the reconnection plane.

While the existence of this suppression region may explain the shortest possible azimuthal extent of dipolarizing flux bundles at Earth (Liu et al. 2015), it may also explain the dawn-dusk asymmetry observed at the magnetotail of Mercury (Sun et al. 2016, 2022), which has a global dawn-dusk extent much shorter than that of Earth.

### Particle Acceleration

There have been quite remarkable advances in using PIC simulations to understand particle acceleration processes in magnetic reconnection, discussed in Oka et al. (2023), Drake et al. (2025), and Guo et al. (2023) of this collection. The simulation provided energetic particle flux, spectra and even detailed distributions that can be compared with in situ observations. We introduce several key diagnostics recently used for gaining insight in particle energization.

**Fig. 10:**
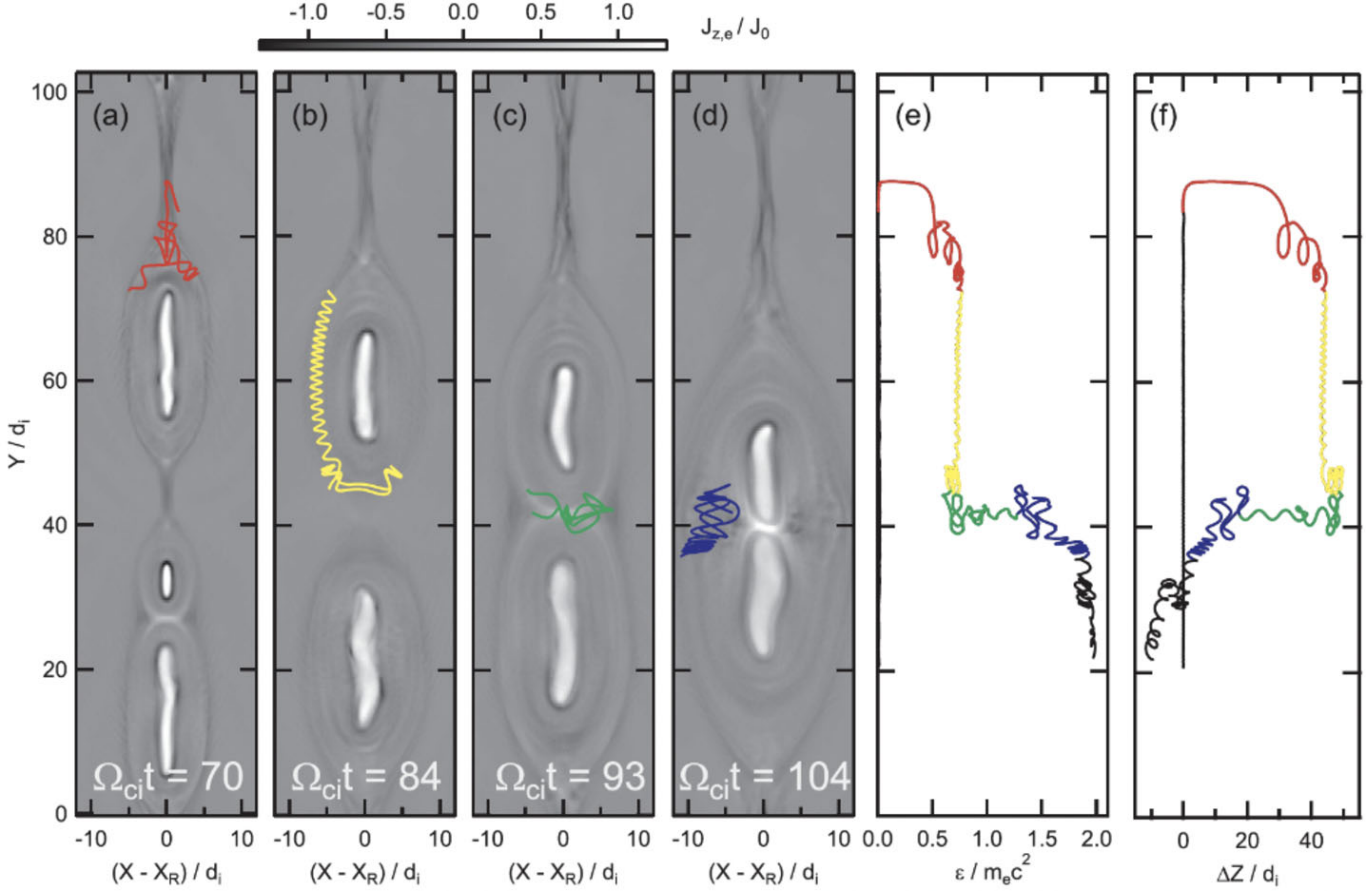
An example of particle trajectory analysis. Adapted from Oka et al. (2010)

First, it has been a common practice to output particle trajectories to study the acceleration process (e.g., Hoshino et al. 2001; Drake et al. 2006; Fu et al. 2006; Oka et al. 2010; Guo et al. 2015). These have led to the identification of different acceleration mechanisms, as discussed in Oka et al. (2023) and Guo et al. (2023) of this collection. Figure 10 shows a representative particle trajectory adapted from Oka et al. (2010). This particle is first accelerated by an X-line (a), then further energized due to electric field during anti-reconnection between two merging island (c). The acceleration persists after the particle is ejected out of the X-line region. In addition, one can output the electric and magnetic fields and other quantities associated with particles, to complement the understanding of acceleration mechanisms.

The limitation of just showing several particle trajectories, even with the best effort, is that the “representative” examples are usually cherry-picking results and it is difficult to evaluate the relative importance of each mechanism. There has been recent effort to evaluate the acceleration processes over a large number of trajectories, (Guo et al. 2019, 2021a; Kilian et al. 2020; French et al. 2022; Li et al. 2023a).

**Fig. 11:**
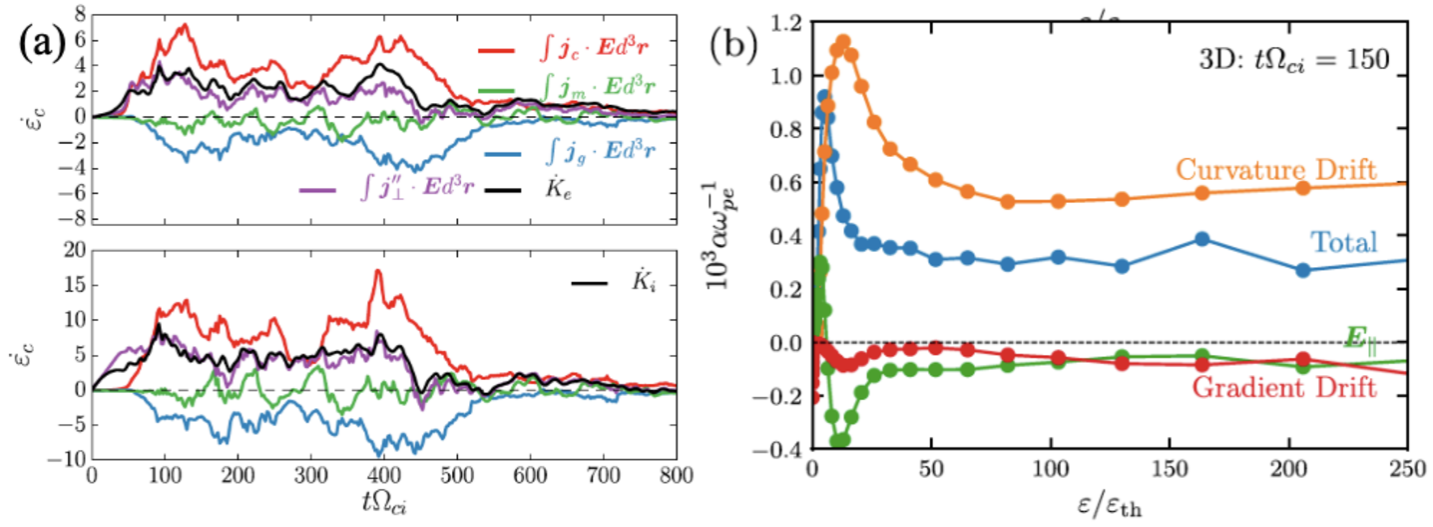
(a) An example of guiding-center drift analysis. Particle energization due to different drift currents for electrons (top) and ions (bottom). $j_{c}$ is due to particle curvature drift. $j_{g}$ is due to particle grad-$B$ drift. $j_{m}$ is due to magnetization. $j\prime \prime = j_{c} +j_{g} +j_{m}$. $\dot{K_{e}}$ and $\dot{K_{i}}$ are the energy change rates for electrons and ions, respectively. They are all normalized by $m_{e}c^{2}\omega _{pe}$. (b) Similar to (a) but shows energy dependent values (Li et al. 2019b) at a given time. Adapted from Li et al. (2017)

Another method, developed and widely used over the last decade, is to study the collective energy gain, such as guiding center and pressure-restrained terms (discussed in Oka et al. 2023, this collection) using ensemble averaged moments (Dahlin et al. 2014, 2015; Li et al. 2015, 2017, 2018, 2019b; Du et al. 2018). For example, the acceleration due to the curvature (Fermi) and gradient (betatron) drifts can be evaluated under guiding center approximation. Figure 11 shows an example under the guiding-center approximation, and shows the curvature drift term is the main acceleration term. Moreover, it is possible to collect the energy dependent acceleration rates by considering particles with different energy, so the energization can be studied in a energy-dependent fashion (Dahlin et al. 2017; Guo et al. 2014; Li et al. 2018, 2019b). Figure 11b shows such an example.

### Simulations of Magnetic Reconnection in Shock Waves

Magnetic reconnection can occur in current sheets generated in plasma turbulence, and PIC simulations have also been applied to turbulent environments (Wu et al. 2013; Matthaeus et al. 2016; Haggerty et al. 2017; Shay et al. 2018; Vega et al. 2020; Adhikari et al. 2021; Rueda et al. 2021), including turbulence in Kelvin Helmholtz vortices in the magnetopause flank region (Nakamura and Daughton 2014; Nakamura et al. 2017, 2022), and the transition region in shock waves (Matsumoto et al. 2015; Bohdan et al. 2017, 2020; Bessho et al. 2019, 2020, 2022, 2023; Ng et al. 2022).

Here, let us review 2D and 3D PIC simulation studies of magnetic reconnection in the shock turbulence. Bessho et al. (2019) used a 2D domain to study a quasi-parallel shock under the parameters in the Earth’s bow shock. The size of the simulation domain is $L_{x}\times L_{y}=375d_{i}\times 51.2d_{i}$, where the ion skin depth $d_{i}$ has 40 grids. The plasma is uniform at $t=0$, both ions and electrons are Maxwellian with their temperatures $T_{i}$ and $T_{e}$, respectively, and the magnetic field is given as $\boldsymbol{B}=[B_{0}\cos \theta , B_{0}\sin \theta , 0]$, where $\theta $ is the shock angle with respect to the $x$ axis. Periodic boundaries are used in the $y$ direction, and conducting walls are placed in the $x$ direction. To all the plasma particles, a negative drift speed, $-v_{d}$, in the $x$ direction is given, and a uniform positive $z$ component of electric field, as $E_{z}=v_{d}B_{0}\sin \theta /c$, is set in the domain. At the right boundary, $x=L_{x}$, new particles for both ions and electrons are injected, using the same temperatures as the initially loaded particles, with the negative drift speed $-v_{d}$. At the left boundary, $x=0$, all the particles are specularly reflected, and the incident particles and the reflected particles generate counter-streaming beams, which cause a beam instability. As a result, a non-linear wave grows near the left boundary, and a wave steepening occurs. Eventually, a shock wave forms, propagating toward the positive $x$ direction.

In the 2D simulation, the following parameters are used: the electron and ion beta $\beta _{e}=\beta _{i}=1$, the ratio of the plasma frequency to the electron cyclotron frequency $\omega _{pe}/ \Omega _{e}=4$, the shock angle $\theta =25^{\circ}$, and the mass ratio $m_{i}/m_{e}=200 $. With these parameters, the electron thermal speed becomes $v_{Te}=14.4v_{A}$. The drift speed is set to be $v_{d}=9v_{A}$. In the simulation (the downstream rest frame), the shock speed is $2.4v_{A}$, which corresponds to the Alfvén Mach number of the shock wave $M_{A}=11.4$. In other words, the shock speed in the laboratory frame is $11.4v_{A}$, which is less than $v_{Te}$, consistent with the Earth’s bow shock.

**Fig. 12:**
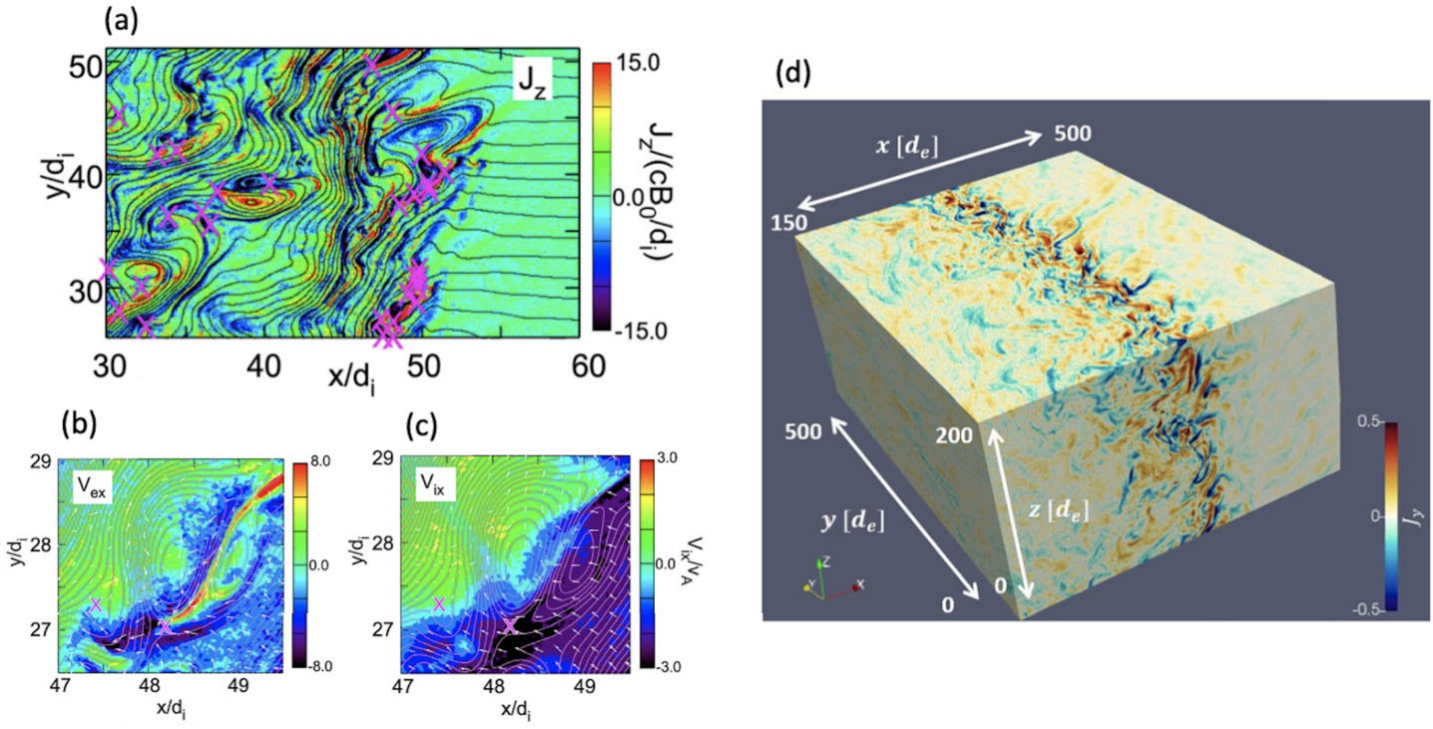
PIC simulations of reconnection in shocks. (a) 2D simulation domain and the current density $J_{z}$. (b) Electron fluid velocity $V_{ex}$. (c) Ion fluid velocity $V_{ix}$. (d) 3D simulation domain and the current density $J_{z}$. Adapted from Bessho et al. (2019) and Ng et al. (2022)

In the simulation, the shock transition region shows a non-resonant ion-ion beam instability due to the interactions between the ions reflected by the shock and the incident ions, and many current sheets are generated, some of which show signatures of magnetic reconnection. In Fig. 12(a), the color shows the current density $J_{z}$ in the 2D simulation domain, where the black curves are magnetic field lines projected onto the $x$-$y$ plane, and magenta X marks represent the positions of reconnection X-lines. One of the reconnecting current sheet is zoomed up in Fig. 12(b) and (c), where the electron fluid velocity $V_{ex}$ and the ion fluid velocity $V_{ix}$ are shown. There is one magnetic island above the current sheet, and there are bipolar electron jets generated from the X-line. In contrast, the ion velocity plot does not show ion jet structures, and the ions are passing through the reconnection region with a negative $V_{ix}$. Therefore, this region is a site of electron-only reconnection, where only electrons are participating in reconnection, while ions cannot respond to the strong gradient of magnetic fields in the thin current sheet, whose thickness is less than the ion skin depth $d_{i}$. Electron-only reconnection has been observed in the Earth’s magnetosheath (Phan et al. 2018; Gingell et al. 2021; Stawarz et al. 2022) and the transition region of the Earth’s bow shock (Wang et al. 2019; Gingell et al. 2019, 2020). Note that the shock transition region has a negative $B_{z}$ magnetic field, $B_{z} \sim -4B_{0}$ (not shown), and the reconnecting magnetic field is the same order. Therefore, in the 2D simulation, reconnection in the shock transition region is guide-field reconnection.

Ng et al. (2022) performed a 3D PIC simulation to study reconnection in the shock transition region. The simulation parameters are: $\beta _{e}=\beta _{i}=1.41$, $\omega _{pe}/\Omega _{e}=4$, $m_{i}/m_{e}=100$, $\theta =30^{\circ}$, $v_{d}=10v_{A}$, and the system size $L_{x}\times L_{y}\times L_{z}=200d_{i}\times 50d_{i} \times 20d_{i}$. The $z$ direction is set to be a periodic boundary. Figure 12(d) shows the current density $J_{z}$. In the 3D simulation, the current direction can be not only in the $z$ direction, but also in the $y$ direction; therefore, the reconnection plane does not have to be in the $x$-$y$ plane as in the 2D simulation, and some current sheets show reconnection with a weak guide field, even though the shock transition region has a large negative $B_{z}$.

## Embedded PIC: MHD-AEPIC

### Overview

Due to the large separation between the kinetic scales and the size of Earth’s magnetosphere, it is highly computationally expensive to apply a purely kinetic code for simulating global magnetospheric dynamics. Various hybrid methods have been proposed to incorporate kinetic physics into global simulations while keeping the computational costs feasible. Traditional hybrid codes model the electron species as a fluid and simulate the ions with either macro-particles or a grid-based Vlasov solver. These hybrid models reduce the separation between the kinetic scales and the global scale by removing the electron kinetic scales from the model so that it becomes feasible to apply them to Earth’s magnetosphere.

The Magnetohydrodynamic with Adaptively Embedded Particle-in-Cell (MHD-AEPIC) model represents another type of hybrid approach to incorporate kinetic effects into global models (Daldorff et al. 2014; Shou et al. 2021; Chen et al. 2023). This type of hybrid model couples a kinetic code with a global fluid model, and only applies the kinetic code to simulate part of the simulation domain, where kinetic physics is crucial while using the fluid model to simulate the rest of the domain. Compared to a purely kinetic model, this type of hybrid model reduces the computational cost by reducing the domain size for the kinetic code, and it is best suited for applications where the important kinetic physics is localized. The MHD-AEPIC model, and its precursor, the Magnetohydrodynamic with Embedded Particle-in-Cell (MHD-EPIC) model, are the first two-way coupled models that work for global applications. Since then, similar coupled models have been developed by different independent teams. For example, Makwana et al. (2017) also developed a model that couples a PIC code with an MHD code, and Rieke et al. (2015) tried to couple a Vlasov solver with a two-fluid code.

### Methodology

#### Development History

**Fig. 13:**
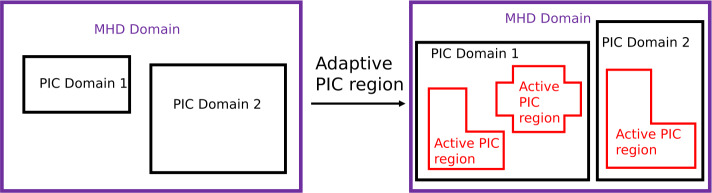
A schematic shows the improvement of the MHD-AEPIC (right) model from the MHD-EPIC (left) model. Adapted from Chen et al. (2023)

The original MHD-EPIC model was developed by Daldorff et al. (2014), in which the semi-implicit particle-in-cell code iPIC3D (Markidis et al. 2010) is coupled with the global fluid model BATS-R-US (Powell et al. 1999) through the Space Weather Modeling Framework (SWMF) (Tóth et al. 2005). The model has been successfully applied to study magnetic reconnections in the magnetospheres of Ganymede (Tóth et al. 2016; Zhou et al. 2019, 2020), Earth (Chen et al. 2017, 2020; Wang et al. 2022a,b), Mercury (Chen et al. 2019) and Mars (Ma et al. 2018). A PIC region has to be a box in the MHD-EPIC model. To cover the kinetic regions of interest, the MHD-EPIC model supports applying multiple independent kinetic regions (Tóth et al. 2016) in the same simulation domain, and it also allows rotating a box so that the corresponding PIC region does not have to be aligned with the global grid (Chen et al. 2020). These two features expand the capabilities of the MHD-EPIC model. However, not all the kinetic regions of interest can be covered by one or a few boxes. If the kinetic region moves at the global spatial scale during a simulation, the PIC box has to be very large to cover the whole region of interest, which is computationally expensive. To overcome these difficulties, the MHD-AEPIC model has been developed, which allows a dynamic PIC region of any shape (see Fig. 13).

To support dynamic PIC regions, two different PIC codes, the Adaptive Mesh Particle Simulator (AMPS) (Shou et al. 2021) and the FLexible Exascale Kinetic Simulator (FLEKS) (Chen et al. 2023), have been developed as the PIC component of the MHD-AEPIC model. Since FLEKS is more widely used for MHD-AEPIC simulations, we focus on the FLEKS code in this section.

#### Coupling Algorithm

MHD-AEPIC supports coupling with single-fluid MHD, multi-species MHD, multi-ion MHD (Glocer et al. 2009b), and five- and six-moment multi-fluid models (Huang et al. 2019). The most widely used fluid component is the single-fluid Hall MHD with a separate electron pressure equation, and we briefly describe the coupling algorithm for this case here.

In an MHD-AEPIC simulation, the PIC code covers part of the whole simulation domain. The MHD model provides the initial conditions for the PIC code at the beginning of the coupled simulation. Once the initialization is done, both the PIC and MHD models update independently for one or a few time steps until the next coupling time point is reached. During the coupling, the MHD model provides the boundary conditions for the PIC code, and the PIC code provides the updated magnetic field and plasma quantities to overwrite the overlapped MHD region. Since the MHD and PIC codes solve different sets of equations, conversion between the MHD and PIC variables is needed. When calculating PIC variables from MHD variables, we need densities, velocities, and pressures for both electron and ion species, and they are calculated as follows:

- Charge neutrality is assumed, so both the electron and ion densities can be easily obtained from total MHD density.
- From the MHD magnetic field, the current density can be calculated. Since the sum of electron and ion momentum is the total MHD momentum, and the velocity difference between electrons and ions produces the current, the electron and ion velocities can be obtained.
- Since we usually solve both ion and electron pressure equations on the MHD side, the electron and ion pressures can be obtained directly to initialize thermal PIC macro-particles.

Once the electron velocity is obtained, it is used to calculate the electric field $\mathbf {E}$ for PIC from the generalized Ohm’s law:

66 $$ \mathbf{E} = -\frac{\mathbf{U}_{e} \times \mathbf{B}}{c}, $$

where $\mathbf {B}$ is the MHD magnetic field and $\mathbf {U}_{e}$ is the electron bulk velocity including the Hall term. We note that no matter Hall physics is included or not into the MHD model, the equation above is applied to calculate the initial and boundary electric field for PIC.

Calculating MHD variables from PIC variables is more straightforward: we simply sum up the mass, momentum and energy of the electron and ion macro-particles to obtain the plasma variables required. We refer the readers to Daldorff et al. (2014) for more details. Currently, the PIC codes used for MHD-EPIC/MHD-AEPIC coupling have to use a Cartesian mesh, but the MHD model BATSU-R-US can use non-uniform Cartesian or non-Cartesian grids. The interpolation between the PIC and MHD grids is done by a second-order linear interpolation.

#### Particle-in-Cell Algorithm

The embedded PIC model is a particular version of the PIC models discussed in detail in Sect. 5. The original MHD-EPIC implementation used the iPIC3D model while MHD-AEPIC uses FLEKS. Both MHD-EPIC and FLEKS are semi-implicit (Brackbill and Lapenta 2008; Lapenta 2017; Chen and Tóth 2019), meaning that the electric field is solved for by an implicit scheme. We choose the semi-implicit PIC algorithm because it has a relaxed stability constraint so that the Debye length does not have to be resolved and the stability constraint for the time step is based on the thermal speed instead of the speed of light. Based on our numerical experiments, we found the stability of the PIC code is extremely important for a successful MHD-EPIC/MHD-AEPIC simulation. To improve the stability, we designed the Gauss’s Law satisfying Energy-Conserving Semi-Implicit Method (GL-ECSIM) (Chen and Tóth 2019), which is based on the Energy-Conserving Semi-Implicit Method (ECSIM) by Lapenta (2017). GL-ECSIM shares the same energy conservation property as ECSIM, i.e., the total energy of the system can be exactly conserved with proper parameters. In practice, we found the code is more stable with parameters that slowly dissipate the total energy numerically. In addition, satisfying Gauss’s law (charge conservation) is also crucial for the stability and accuracy of the PIC code. GL-ECSIM applies a novel method to satisfy Gauss’s lay by adjusting particle positions at the end of each cycle. The details of GL-ECSIM can be found in Chen and Tóth (2019).

In a long MHD-AEPIC simulation, the macro-particle number per cell may vary significantly due to the transport of particles. The uneven distribution of particle numbers can cause load imbalance and reduce computational efficiency. To alleviate this problem, we designed particle splitting and merging algorithms for FLEKS. A particle splitting (merging) algorithm is applied to split (merge) particles when the number of particles per cell is below (above) a threshold (Chen et al. 2023).

#### Kinetic Region Adaptation

The most important improvement of MHD-AEPIC over MHD-EPIC is the adaptive PIC region. Although the PIC grid is still Cartesian, its cells can be switched on or off so that the active cells can fit any shape of kinetic regions. We note that the PIC cells can be activated or deactivated dynamically during a simulation. The active PIC region can be defined either based on geometric or physical criteria. For physics-based adaptation, BATS-R-US calculates the physical criteria and sends the corresponding grid information to FLEKS to turn on or turn off cells.

#### Kinetic Scaling

In some applications, the difference between kinetic and global spatial and temporal scales makes it difficult, if not impossible, to resolve the kinetic scales in an MHD-EPIC, or even MHD-AEPIC simulation. Fortunately, the large separation of scales can be exploited, and the kinetic scales can be increased by changing the mass per charge ratio without affecting the global dynamics (Tóth et al. 2017). This technique is not needed or even applicable for Ganymede and Mercury simulations, where the kinetic and global scales are not very different. On the other hand, kinetic scaling is applicable and extremely useful for modeling Earth’s magnetosphere. We typically increase the kinetic scales by a factor of 4 to 16. See Tóth et al. (2017) for more detail.

### Applications

The MHD-EPIC/MHD-AEPIC model has been applied to investigate the physical processes and consequences of both magnetopause and magnetotail reconnection. In these simulations, the PIC code is usually used to cover either the magnetopause or the magnetotail current sheet, where reconnection happens. Since the initial and boundary conditions of the PIC code are obtained from the MHD model, the physical parameters inside the PIC region, such as the plasma quantities and the shape of the current sheet, are more realistic than those in a standalone PIC simulation. On the other hand, the information from the PIC code is also fed back to the MHD model so that we can evaluate the global consequences of the kinetic magnetic reconnection.

**Fig. 14:**
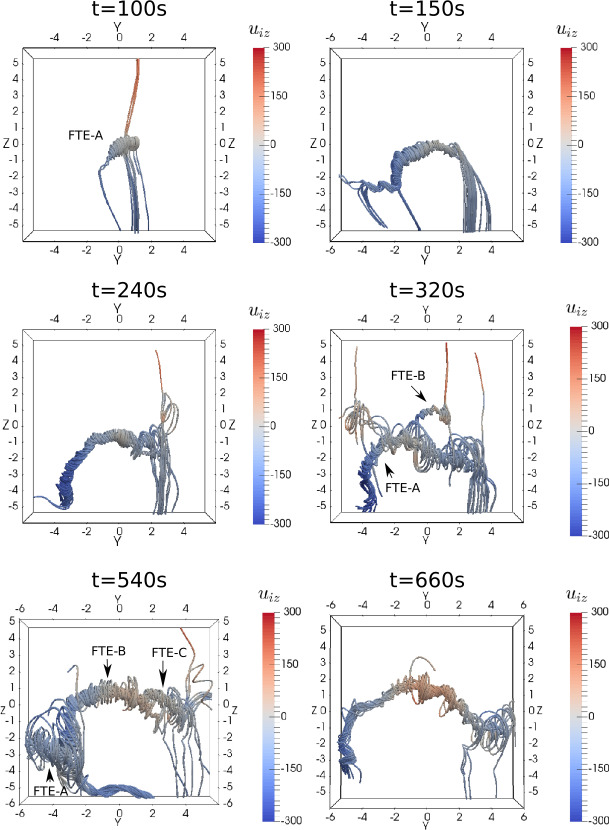
Evolution of FTEs. Viewed from the Sun, a series of snapshots are shown with magnetic field lines colored by ion velocity $u_{iz}[\mathrm{km/s}]$. Adapted from Chen et al. (2017)
- Since its ambient plasma flow speed varies, an FTE may become tilted ($t=240~\text{s}$ in Fig. 14).
- There may be multiple FTEs on the magnetopause, and a few FTEs can merge into one (from $t=320$ to $t=660~\text{s}$ in Fig. 14).
- FTEs can be dissipated at high latitudes due to the reconnection between the FTE magnetic field and the cusp field lines (Fig. 5 of Chen et al. 2017).

Here we briefly describe a few applications of the MHD-EPIC/MHD-AEPIC model to study Earth’s magnetosphere. Chen et al. (2017) studied both the kinetic features of magnetopause reconnection and the evolution of flux transfer events (FTEs) show in Fig. 14. Near the reconnection site, the simulation successfully produced key kinetic features of asymmetric magnetic reconnection, such as the crescent electron phase space distribution and the lower hybrid drift instability. Due to the multiple X-line reconnections inside the PIC code, FTEs are generated quasi-periodically at low latitudes, then propagate toward the cusps. We briefly describe the evolution of the FTEs here:

- During the growth of an FTE, its cross-section increase, and its length extends along the dawn-dusk direction (from $t=100~\text{s}$ to $t=150~\text{s}$ in Fig. 14).

Chen et al. (2020) simulated the GEM dayside kinetic processes challenge event, and compared simulation results with both MMS observations and ground-based SuperDARN observations. The MHD-EPIC simulation shows there are usually multiple X-lines at the magnetopause, and the expanding speed of the X-line endpoints is comparable with SuperDARN observations.

The MHD-EPIC model has also been applied to study magnetotail reconnection. Wang et al. (2022b) found the MHD-EPIC simulation can produce global-scale magnetospheric sawtooth-like oscillations periodically even under steady solar wind conditions, while the ideal- and Hall-MHD simulations do not produce such variations. It suggests that kinetic reconnection physics may play an important role in driving sawtooth oscillations. Recently, the development of the MHD-AEPIC model has enabled us to simulate storm events with a dynamic PIC region that covers the highly dynamic magnetotail reconnection sites.

Currently, research employing MHD-AEPIC focuses on modeling extreme geomagnetic storm events. Extreme events occur infrequently, which makes it difficult to validate MHD models employing simple numerical diffusion to approximate reconnection physics. Using a higher-fidelity model, such as MHD-AEPIC, can improve the reliability of simulations of extreme events.

## Kglobal: Particle Acceleration Self-Consistently Embedded in a Fluid Model

Solar flares convert magnetic energy into particle energy via magnetic reconnection. Observations of power-law tails in particle distribution functions imply that a large fraction of the released energy goes to energetic (i.e., non-thermal) electrons and ions (Warmuth and Mann 2016). However, the particle spectra found in particle-in-cell (PIC) simulations of reconnection in the relevant regime typically do not form power-laws, except in the limit of extremely low upstream plasma $\beta $ (Dahlin et al. 2015, 2017; Zhang et al. 2021). Why? With structures extending $\sim 10^{4}\text{ km}$ and a Debye length of $\sim 1\text{ cm}$ (for $n \sim 10^{10}\text{ cm}^{-3}$ and $T_{e} \sim 100\text{ eV}$), the corona spans ten orders of magnitude in physical scale. Explicit PIC models must resolve kinetic scales and hence can only simulate a tiny fraction of the macroscopic domain. The dependence of the Larmor radius on energy means nonthermal particles can quickly acquire orbits that approach the size of the simulation domain, halting further energy gains.

In contrast, MHD simulations study macroscopic domains with a fluid description that averages over small spatial and temporal scales. Following test particles in the MHD fields produces information about how particles gain energy but, without feedback coupling the particles and the fields, runaway energy gain can occur so that the system as a whole does not conserve total energy. It is possible to embed PIC models into MHD descriptions at selected locations, but such models presume that particle energy gain occurs in the vicinity of magnetic nulls, which is not consistent with the development and interaction of macroscale magnetic islands or the development of turbulence in large-scale current layers.

The *kglobal* model incorporates the physics necessary to explore particle energization from both the PIC and MHD descriptions (Drake et al. 2019; Arnold et al. 2019). The fundamental question is whether kinetic-scale boundary layers play an essential role in particle energy gain – or if they can be ordered out of the equations to facilitate simulations of macroscale systems. Kinetic boundary layers control the regions where $E_{\parallel}$, the component of the electric field parallel to the magnetic field, is non-zero. However, Fermi reflection rather than $E_{\parallel}$ is the dominant driver of energetic particles (Dahlin et al. 2016; Li et al. 2019a). Particle energy gain from Fermi reflection takes place over macro-scale regions and occurs even where $E_{\parallel} = 0$. As a consequence, kinetic-scale boundary layers are not required to describe the non-thermal energization in macroscale systems.

In order to keep the physics most important for describing particle energization while still being able to model macroscale systems, *kglobal* combines aspects of these descriptions. Guiding center particles move through a computational domain that includes a grid for fluid quantities. These particles feed back on the fluid through their gyrotropic pressure tensor. They can be small in number density but can contribute a pressure comparable to the pressure of the reconnecting magnetic field. The entire system conserves total energy.

The basic version of *kglobal* includes three species: fluid ions, fluid electrons, and particle electrons (the latter of which form the nonthermal population). We note that in a very strict sense the model may be viewed as multifluid plus guiding center methods. However, the motion of the fluid electrons is severely constrained: their density is determined by quasi-neutrality, their parallel velocity from the requirement that parallel currents vanish, and their perpendicular velocity equal to the $E\times B$ flow (as it is for every species). The electromagnetic fields follow the usual MHD equations

67 $$ \frac{\partial \mathbf{B}}{\partial t} = -c\boldsymbol{\nabla \times} \mathbf{E}_{\perp} \qquad \mathbf{E}_{\perp} = -\frac{1}{c}\mathbf{v}_{i} \boldsymbol{\times}\mathbf{B} $$

and the ion fluid satisfies the usual MHD continuity equation

68 $$ \frac{\partial n_{i}}{\partial t}+ \boldsymbol{\nabla \cdot}\,n_{i} \mathbf{v}_{i} = 0 $$

and energy equation

69 $$ \frac{d}{dt}\left (\frac{P_{i}}{n_{i}^{\gamma}}\right ) = 0 $$

The ion momentum equation takes the form

70 $$ \rho _{i}\frac{d\mathbf{v}_{i}}{dt} = \frac{1}{c}\mathbf{J} \boldsymbol{\times}\mathbf{B} - \boldsymbol{\nabla}P_{i} - \boldsymbol{\nabla}_{\perp} P_{ef} - m_{e}n_{ef}v^{2}_{\parallel ef} \boldsymbol{\kappa} + en_{i}E_{\parallel}\mathbf{b}- ( \boldsymbol{\nabla \cdot}\,\mathrm{T}_{ep})_{\perp} $$

in which the left-hand side and first terms on the right-hand side are the same as in MHD ($P_{i}$ and $P_{ef}$ are the ion and fluid electron pressure, respectively). However the final terms on the right-hand side include the curvature $\boldsymbol{\kappa} = \mathbf{b}\boldsymbol{\cdot \nabla}\mathbf{b}$, large-scale parallel electric field $E_{\parallel}$, and particle electron stress tensor $\mathrm{T_{ep}}$ and quantify the self-consistent back-reaction of the particles on the system.

The perpendicular motion of the particle electrons is given by the conservation of the first adiabatic invariant

71 $$ \mu _{ep} = \frac{p_{ep\perp}^{2}}{2B}=\text{const.} $$

while the parallel motion satisfies

72 $$ \frac{d}{dt}p_{e\parallel} = p_{e\parallel}\mathbf{v_{E}} \boldsymbol{\cdot \kappa} - \frac{\mu _{e}}{\gamma _{e}}\mathbf{b} \boldsymbol{\cdot \nabla}B - eE_{\parallel} $$

This equation includes a contribution from the parallel electric field given by

73 $$ E_{\parallel} = -\frac{1}{n_{i}e}\left (\mathbf{B} \boldsymbol{\cdot \nabla}\left ( \frac{m_{e}n_{ef}v^{2}_{ef\parallel}}{B}\right ) + \mathbf{b} \boldsymbol{\cdot \nabla}P_{c} + \mathbf{b} \boldsymbol{\cdot \nabla \cdot}\mathrm{T}_{ep}\right ) $$

Finally, the fluid electron density enforces quasi-neutrality

74 $$ n_{ef} = n_{i} - n_{ep} $$

the parallel flow eliminates parallel currents

75 $$ n_{ef}v_{ef\parallel} = n_{i}v_{i\parallel} - n_{ep}v_{ep\parallel} $$

and the pressure equation takes the usual form

76 $$ \frac{d}{dt}\left (\frac{P_{ef}}{n_{ef}^{\gamma}}\right ) = 0 $$

A full derivation of these equations is given in Drake et al. (2019) and Arnold et al. (2019).

Simulations with these equations pass several tests. They describe the linear propagation of stable, circularly polarized Alfvén waves and the linear growth of firehose modes. The latter plays an important role in controlling the feedback of energetic particles during magnetic reconnection since magnetic tension is suppressed on the approach to firehose marginal stability. In addition, they accurately capture the dynamics of electron acoustic waves and describe the suppression of transport of hot electrons parallel to the ambient magnetic field. The inclusion of the large scale $E_{\parallel}$ is important in describing the development of return currents that form as hot electrons escape from regions of electron acceleration in macroscale energy release events such as flares (Egedal et al. 2012).

**Fig. 15:**
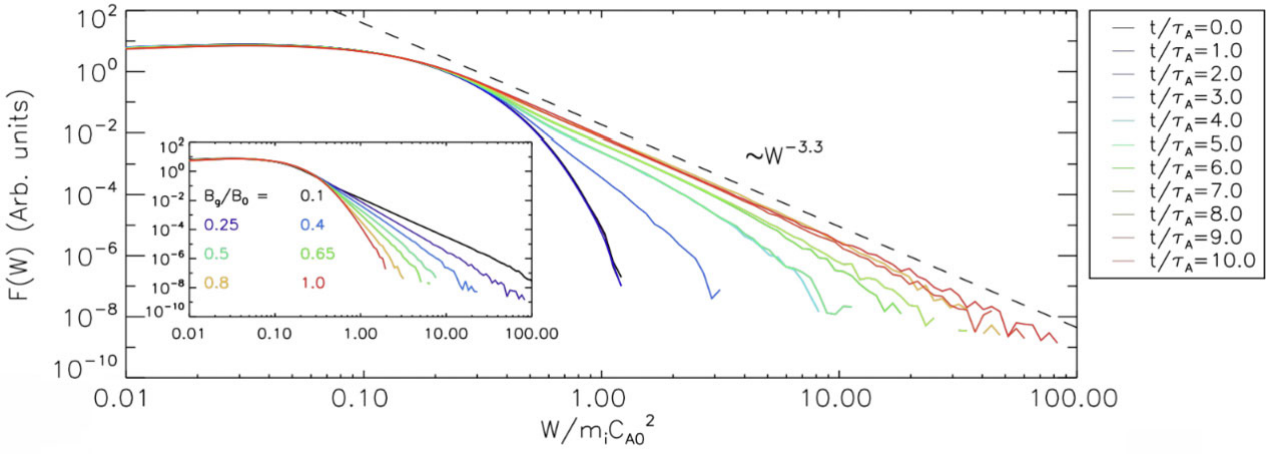
Energetic electron spectra from *kglobal*. A log-log plot of the electron differential density F(W) versus energy ($W$) at multiple times from a reconnection simulation with a guide field $B_{g}/B_{0} = 0.25$. A power-law develops after $t/\tau _{A} \sim 3-5$. Inset: The late-time F(W) for several guide fields, illustrating the dependence on the *ratio* of the guide-to-ambient magnetic field. Adapted from Arnold et al. (2021)

Reconnection simulations with *kglobal* have produced power-law spectra of energetic electrons that extend nearly three decades in energy, while simultaneously generating the super-hot thermal electrons characteristic of flare observations (Arnold et al. 2021). Figure 15 shows the electron energy spectrum for a typical simulation. Electrons in the initial Maxwellian distribution (black curve) transform into a nonthermal spectrum in a few Alfvén crossing times ($\tau _{A}$). Consistent with observations, the total energy content of the nonthermal electrons can exceed that of the hot thermal electrons even though the number density does not. The strength of the ambient out-of-plane guide field strongly impacts the energy content and power-law index of the nonthermal electrons (see inset of Fig. 15): the guide field increases the radius of curvature of a reconnected field line, thereby weakening Fermi reflection (Drake et al. 2006). In contrast, the size of the global system has relatively little influence.

The governing equations of *kglobal* can be extended to include the contributions of non-thermal (particle) ions. Unlike for electrons, whose small mass can be used to simplify the equations, the ion inertia can not be neglected and must be included. Recent work has incorporated these equations into the computational model and early results reveal the simultaneous development and evolution of extended electron and proton power law distributions (Yin et al. 2024a,b).

## Vlasov

### Overview

Eulerian Vlasov-Maxwell numerical simulations are a useful tool for investigating fundamental kinetic-scale plasma processes, such as turbulence and magnetic reconnection, as well as the interaction between the solar wind and planetary magnetospheres.

Thanks to the clean description of the plasma dynamics in the entire phase space at the expense of a larger computational cost, Eulerian algorithms complement well Particle-In-Cell (PIC) codes. The almost noise-free description of velocity space is generally guaranteed by the discretization of the plasma distribution function on a six-dimensional phase-space grid characterized by collocation points in both physical and velocity space. On the other hand, PIC methods suffer from the intrinsic stochastic shot noise which becomes especially relevant at small scales and in cases where the number of particles per cell is not large. However, in Eulerian methods, setting a six-dimensional grid in the entire phase space dramatically increases the computational cost. The bottleneck is generally constituted by the memory necessary to store the plasma distribution function, as shown in the following simple example.

**Fig. 16:**
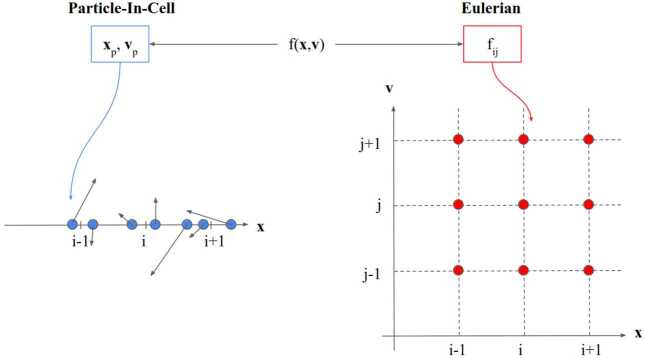
Sketch of the typical sampling of the plasma distribution function $f({\mathbf{x}},{\mathbf{v}})$ adopted in PIC (left) and Eulerian (methods). Adapted from Finelli (2022)

The main difference when sampling the plasma distribution function in PIC and Eulerian approaches is depicted in Fig. 16. In PIC methods, the grid is defined only on the physical space ${\mathbf{x}}$, and the distribution function is sampled through macroparticles (blue circles), each one representative of a large number of effective plasma particles. In the Eulerian approach, the grid is defined on the entire phase space $({\mathbf{x}}, {\mathbf{v}})$ and the distribution function is known on this ensemble of grid points (red circles). In both PIC and Eulerian methods, the electromagnetic fields and the moments of the distribution function (e.g., density, bulk speed, etc.) are defined on the physical-space grid ${\mathbf{x}}$.

Let us imagine a generic phenomenon occurring in a plasma composed of protons and electrons that requires a physical-space grid discretized with $512^{3}$ points. Since the electromagnetic fields have the same memory requirements in both PIC and Eulerian methods, we neglect them here for simplicity. PIC simulations indicate that the plasma dynamics is overall well described with $\sim 1000$ particles per cell. The memory (assuming double-precision variables) required to store particles positions and velocities is $\sim 6$ TB. Similarly, Eulerian Vlasov simulations with a homogeneous Cartesian grid in velocity space, generally adopt at least $\sim 51^{3}$ velocity-space points to well describe the fine details of the velocity plasma distribution function and preserve mass and entropy conservation, thus requiring about $\sim 260$ TB of memory. Hence, PIC simulations can usually be performed in larger physical-space computational boxes compared to Eulerian ones. Moreover, 3D simulations are more easily achievable with PIC methods while they remain often prohibitive for Eulerian codes, or require more advanced methods such as adaptive mesh refinement or sparse velocity space techniques to become tractable.

In the following, we will introduce two Vlasov-Maxwell algorithms that have been intensively used by the scientific community — namely the Vlasiator code (von Alfthan et al. 2014; Palmroth et al. 2018a) and the Hybrid Vlasov-Maxwell HVM code (Valentini et al. 2007) — and, then, discuss the results of global and local simulations of magnetic reconnection based on these two codes.

### Models and Algorithms

Picturing a virtual journey from large to small scales, the first relevant Vlasov-Maxwell model widely adopted for performing Eulerian simulations is the hybrid Vlasov-Maxwell model. The hybrid model considers protons as a kinetic species, while electrons are a background fluid. It is a low-frequency approximation of the full Vlasov-Maxwell system of equations that assumes quasi-neutrality and neglects the displacement current (Mangeney et al. 2002). Faraday’s law is used to evolve the magnetic field, while the electric field is provided by the generalized Ohm’s law which includes the Hall term, the electron pressure gradient term, and possibly the terms related to electron inertia (see, e.g., Valentini et al. 2007 for further details about the generalized Ohm’s law).

The following paragraphs present Vlasiator (von Alfthan et al. 2014; Palmroth et al. 2018a) and the Hybrid Vlasov-Maxwell (HVM) (Valentini et al. 2007) codes as particular examples of algorithms based on the Vlasov approach. The reader is invited to refer to recent review papers (Califano and Cerri 2022; Palmroth et al. 2018a) for more details on how to solve the Vlasov equation numerically.

The hybrid Vlasov-Maxwell model has been adopted for global simulations of the interaction between the solar wind and the Earth’s magnetosphere through the Vlasiator algorithm (von Alfthan et al. 2014; Palmroth et al. 2018a). The Vlasiator code adopts a splitting algorithm to decompose the six-dimensional Vlasov equation into a set of two three-dimensional advection equations (Strang 1968) in physical and velocity space, respectively. The solution of each advection equation is obtained by a semi-Lagrangian method (Zerroukat and Allen 2012), which relieves from the strict limitations to the time step length posed by the CFL condition^1^ in velocity space acceleration in regions of strong magnetic field. Position space is discretized on a cell-adaptive Cartesian grid (Honkonen et al. 2013; Ganse et al. 2023) and at each position in space the velocity-space grid is stored on a uniform, Cartesian grid. Vlasiator developed a sparse velocity-space method in which only regions of the velocity distribution function above a set phase-space density are stored and propagated (von Alfthan et al. 2014), yielding a gain of two orders of magnitude in terms of memory and computations. This technique made two-dimensional (2D position space periodic in the third dimension, 3D velocity space) magnetospheric simulations possible, as well as quasi-three dimensional simulations with a very limited extent in the third dimension (Pfau-Kempf et al. 2020). The implementation of adaptive mesh refinement in position space, allowing to focus resolution on regions of interest while saving computations in less-resolved regions, is what made full three-dimensional, global magnetospheric simulations achievable with Vlasiator on modern, bleeding-edge supercomputers (Grandin et al. 2023; Ganse et al. 2023; Palmroth et al. 2023). The electric and magnetic fields are propagated using an upwind constrained transport method (Londrillo and Del Zanna 2004) with divergence-free magnetic field reconstruction (Balsara 2009) on a uniform Cartesian grid matching the finest refinement level of the Vlasov spatial grid, requiring a dedicated coupling scheme between the grids (Papadakis et al. 2022). Note that, despite the sparse velocity-space grid, Vlasiator also requires a huge amount of RAM memory similar, as order of magnitude, with the simple estimate discussed above.

The hybrid Vlasov-Maxwell model has also been adopted for local simulations of plasma turbulence at sub-proton scales using the HVM code (Valentini et al. 2007) (see Califano and Cerri 2022 for a recent review). The HVM code reduces the six-dimensional Vlasov equation to a set of six one-dimensional advection equations. Each equation is then solved through the van Leer method (van Leer 1977). Fields are computed through the Current-Advance Method (CAM). The grid is homogeneous in both physical and velocity space. Periodic boundary conditions are implemented in physical space. In velocity space, the proton distribution function is set to zero after a large number of thermal speeds, thus ensuring that mass conservation is preserved. Numerical resolution in physical space is usually about 4-5 gridpoints per ion skin depth, while velocity-space grid resolution is of about $0.2-0.25$ ion thermal speed. The latter condition is an optimal compromise to (i) guarantee that velocity-space filamentation and distortion of the ion distribution function are well observed in velocity space, thus allowing to investigate energy conversion in the entire phase space, (ii) preserve the entropy conservation to an excellent value ($\lesssim 0.1 \%$), and (iii) maintain simulations numerically feasible.

Moving towards smaller scales, fully-kinetic Vlasov-Maxwell Eulerian algorithms have been recently implemented to describe electron-scale dynamics. Given the larger computational cost of Eulerian simulations with respect to PIC methods, the former are generally more recent than the latter and possibly implement different assumptions to simplify the Maxwell equations. The full Maxwell system has been retained in several codes (Umeda et al. 2009, 2010; Delzanno 2015; Ghizzo et al. 2017; Juno et al. 2018; Pezzi et al. 2019a; Allmann-Rahn et al. 2022). However, different approximations of the Maxwell equations have been proposed to alleviate the CFL constraint which sets a very small time step when the wave phase speed approaches the speed of light. In this regard, Wiegelmann and Büchner (2001) neglect the displacement current while allowing for charge separation, while Tronci and Camporeale (2015) ignore both the displacement current and the charge separation. Yet a different approach neglects only the transverse part of the displacement current responsible for ordinary mode propagating at the speed of lights (Schmitz and Grauer 2006a; Pezzi et al. 2019a; Shiroto 2023). Finally, an original approach has been developed based on the Vlasiator model, whereby a small section of interest from an ion-hybrid Vlasiator run is used to initialize an electron-hybrid setup in which the ions are kept static while the electron distribution function evolves (Battarbee et al. 2021). This so-called eVlasiator approach has successfully reproduced properties of electron distributions observed in the vicinity of reconnection diffusion regions (Alho et al. 2022).

### Examples of Applications Focused on the Study of Magnetic Reconnection

#### Local Simulations

In this section, we will present key results relevant to magnetic reconnection that have been obtained with the Hybrid Vlasov Maxwell code (HVM). Further treatments, based on fully-kinetic Eulerian Vlasov-Maxwell simulations, will not be covered in detail in this article, but the reader is referred to the works of Schmitz and Grauer (2006b), Inglebert et al. (2011), Zenitani and Umeda (2014), Sarrat et al. (2017), Pezzi et al. (2019a), as well as Table 2 in the review by Palmroth et al. (2018a) which lists works using Vlasov-based methods in space and astrophysics.

The HVM code, which retains alpha particles (Perrone et al. 2012; Valentini et al. 2016) and inter-particle collisions (Pezzi et al. 2019b), has been used for years to investigate plasma processes occurring at ion kinetic scales. It has been massively employed to study the properties of plasma turbulence (Valentini et al. 2010; Servidio et al. 2012, 2014, 2015; Cerri et al. 2017), showing that turbulent fluctuations generate manifestly non-Maxwellian proton distribution functions (Greco et al. 2012). This emergent velocity-space complexity has been envisioned as a cascade process occurring in velocity space (e.g., Tatsuno et al. 2009; Schekochihin et al. 2016; Servidio et al. 2017): HVM results have allowed to characterize it in a full Vlasov system rather than in the gyrokinetic approximation (Cerri et al. 2018; Pezzi et al. 2018, 2021b). Characterizing non-equilibrium plasma distribution functions is significant to understanding energy transfer and dissipation processes occurring at ion kinetic scales in nearly-collisionless plasmas such as the solar wind (Matthaeus et al. 2020; Cassak et al. 2023), as also reported in different studies based on the HVM code (Sorriso-Valvo et al. 2018; Pezzi et al. 2019c, 2021a; Fadanelli et al. 2021). In the perspective of the Holloway and Dorning (1991) work showing that non-Maxwellian plasmas can support the propagation of undamped plasma waves, the HVM code has been adopted to study the onset of a novel type of electrostatic fluctuations triggered by trapped ions (Valentini et al. 2011a,b, 2014).

One of the first studies employing the HVM code for investigating magnetic reconnection reported the onset of a fast reconnection process obtained as a result of magnetic islands developed by the electromagnetic current filamentation (Califano et al. 2001). In the following years, despite the large number of studies adopting the HVM code, the vast majority of the research work mostly focused on the investigation of fully developed plasma turbulence. More recently, Finelli et al. (2021) investigated the magnetic reconnection in a similar manner as PIC-based studies discussed in Sect. 5, that is, modeling an isolated Harris-like current sheet (Harris 1962b) in equilibrium or pressure balance. Such a current sheet, usually doubled in the physical-space domain to accommodate for periodic boundary conditions, quickly starts reconnecting thanks to an initial perturbation of proton density and/or current (thus magnetic field).

**Fig. 17:**
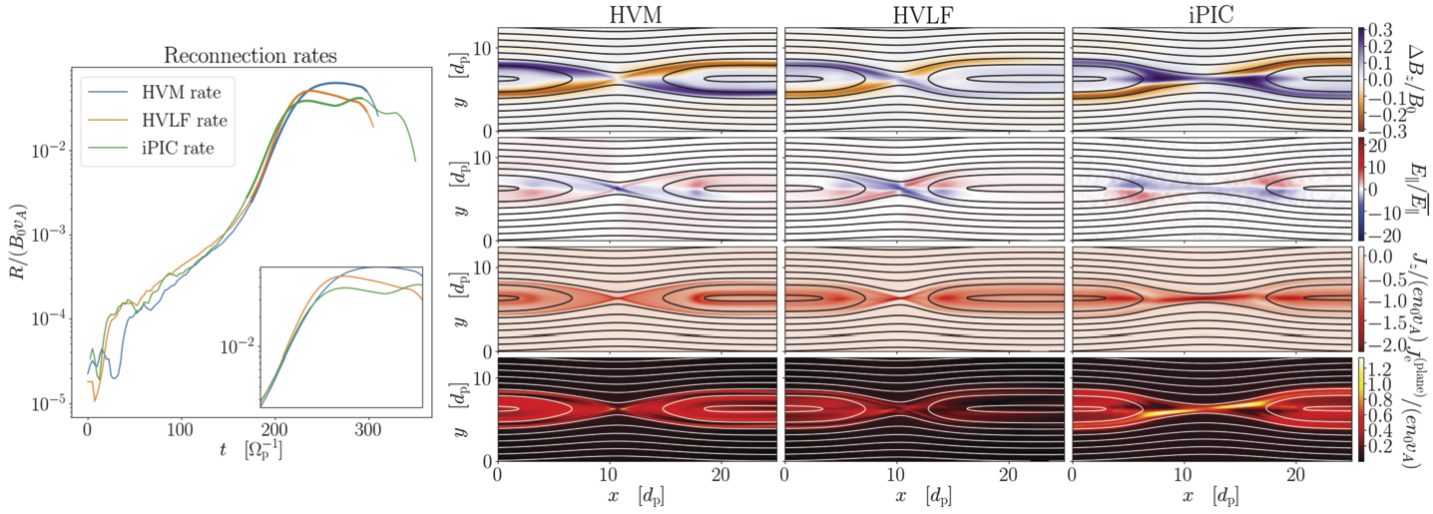
Magnetic reconnection modeled using three different codes (HVM, HVLF and PIC). Left: normalized reconnection rate $R/B_{0} v_{\mathrm{A}}$. The inset shows the time interval corresponding to the ticker curves in the main plot. To ease the comparison, the curves in the inset are shifted in time. Right: (first row) out-of-reconnection-plane magnetic field $\Delta B_{z}/B_{0} = (B_{z} - B_{z}(t=0))/B_{0}$ showing the expected Hall quadrupolar pattern; (second row) electric field parallel to the ambient magnetic field $E_{\parallel}/\overline{E_{\parallel}}$, where $\overline{E_{\parallel}}$ is the root mean square of $E_{\parallel}$ in the shown region; (third row) current density in the out-of-plane direction $J_{z}$; (fourth row) electron current density in the plane $J_{\mathrm{e}}^{\mathrm{(in}-\mathrm{plane)}}$. The superposed black or white curves are the magnetic field lines. The three columns show results from the three different models. The left column show results from HVM at the simulation time $t = 237.5 \ \Omega _{\mathrm{cp}}^{-1}$; the center column show results from the HVLF code at the simulation time $t = 232.5 \ \Omega _{\mathrm{cp}}^{-1}$; the right column show results from the PIC code at the simulation time $t = 235.0 \ \Omega _{\mathrm{cp}}^{-1}$. $\Omega _{\mathrm{cp}}$ is the proton cyclotron frequency. Adapted from Finelli et al. (2021)

In particular, Finelli et al. (2021) compared results from three different models (i) the HVM model with (isotropic) isothermal electrons including finite electron-inertia, (ii) a modified HVM model, called hybrid-Vlasov-Landau-fluid (HVLF); (iii) a fully-kinetic PIC code (iPIC3D Markidis et al. 2010). The HVLF model is equipped to include anisotropies of the gyrotropic electron pressure with a Landau-fluid (LF) closure for the transport of the gyrotropic electron thermal energy along magnetic field lines (Sulem and Passot 2015). Using these three models, Finelli et al. (2021) performed 2D-3V magnetic reconnection simulations with moderate guide field ($B_{g} = 0.25 \ B_{0}$, where $B_{0}$ is the asymptotic magnetic field) and with reduced mass ratio $m_{\mathrm{p}}/m_{\mathrm{e}} = 100$. The initial setup consists of a double Harris current sheet (Harris 1962b) perturbed by long wavelength magnetic field fluctuations with random phase (with $1 < |\mathbf{k}| d_{\mathrm{p}} < 9$, where $\mathbf{k}$ is the wave vector of the fluctuations and $d_{\mathrm{p}}$ is the proton inertial length). The size of the simulations domains is $L_{x} \times L_{y} = 24 \pi d_{\mathrm{p}} \times 12 \pi d_{ \mathrm{p}} $ discretized with $N_{x} \times N_{y} = 1024 \times 512$ grid points. In the HVM and HVLF simulations, the velocity space domain in each direction ($x$, $y$ and $z$) is [−6.4, +6.4] $v_{th,p}$, where $v_{th,p}$ is the proton thermal speed, and it is discretized by $51^{3}$ grid points. Figure 17 shows results comparing the three models.

While the reconnection linear phase evolution, as well as the overall reconnection signatures and patterns, are quite similar for all three models, Finelli et al. (2021) report that the region of intense current at the centre of the current sheet is more elongated in the case of the HVLF and PIC simulations than in the HVM run. Also, the normalized reconnection rate $R/B_{0} v_{A}$ computed in the quasi-steady state is higher for the HVM ($R/B_{0} v_{A} \sim 0.06$, where $B_{0}$ is the upstream magnetic field and $v_{A}$ is the Alfvén speed) than for the HVLF and PIC simulations ($R/B_{0} v_{A} \sim 0.04$). Despite these differences, the results of all three simulations agree qualitatively. In terms of electron dynamics, which is not captured by the HVM code, the HVLF model reproduces the main features obtained with the fully kinetic treatment of the PIC code.

Magnetic reconnection and turbulence are intricately coupled in plasmas (Stawarz et al. 2024, this collection), where coherent structures such as magnetic holes, magnetic islands, and current sheets naturally develop (see, e.g., Matthaeus et al. 2015). Then, current sheet widths tend to approach the kinetic scale, leading to magnetic reconnection. Hybrid-Vlasov simulations of turbulent plasmas have been successful in modelling both turbulence-induced “standard” reconnection with ion-coupling and electron-only magnetic reconnection (Califano et al. 2020; Arrò et al. 2020). A key result in the context of the interplay between reconnection and turbulence is the fact that turbulence is mediated by magnetic reconnection. More specifically, reconnection plays a key role in driving the onset of sub-ion turbulent cascade (Franci et al. 2017; Cerri et al. 2017; Manzini et al. 2023; Adhikari et al. 2024). Hybrid-Vlasov simulations with the HVM code have played a crucial role in providing evidence for the role played by reconnection in this context.

#### Global Simulations

The main goal of Vlasiator is to model the solar wind–magnetosphere interaction with a hybrid-Vlasov approach. For this reason, the computational efforts have been mainly directed toward performing global simulations of the entire magnetosphere. As a consequence, a broad variety of magnetospheric plasma phenomena have been investigated using Vlasiator (notably collisionless shock (e.g., Johlander et al. 2022) and foreshock physics (e.g. Turc et al. 2023), magnetosheath jets (e.g. Suni et al. 2021), auroral proton precipitation (e.g. Grandin et al. 2020, 2023) to mention a few). As magnetic reconnection plays a key role in magnetosphere dynamics, it has been investigated in several Vlasiator studies, both at the magnetopause (Pfau-Kempf et al. 2016; Hoilijoki et al. 2017, 2019; Akhavan-Tafti et al. 2020; Pfau-Kempf et al. 2020) and in the magnetotail (Palmroth et al. 2017; Juusola et al. 2018; Runov et al. 2021; Palmroth et al. 2023). Since Vlasiator does not include an explicit resistive term, magnetic reconnection is enabled by the numerical diffusivity or resistivity.

In this section, we present key results from selected Vlasiator studies in two subsections, one devoted to magnetopause reconnection and the other focusing on magnetotail reconnection. We start with discussing 2D-3V simulations, and then present 3D-3V simulations since recent algorithmic improvements allowed running global, three-dimensional (3D-3V) hybrid-Vlasov simulations of Earth’s magnetosphere (Ganse et al. 2023).

**Fig. 18:**
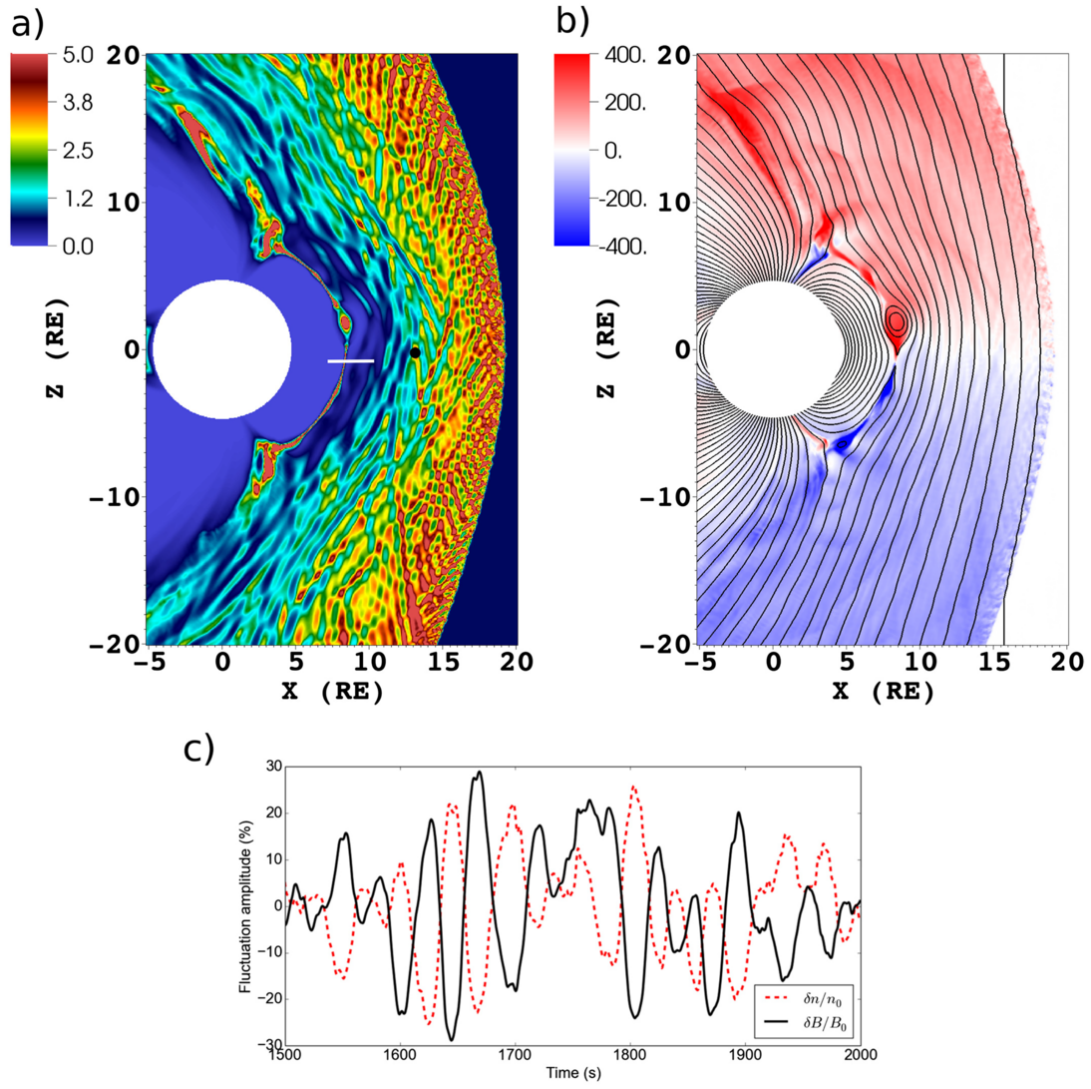
(a) Plasma $\beta $; (b) proton $V_{z}$. The black lines show the magnetic field lines. (c) Magnetic field strength (black solid) and plasma density (red dashed) fluctuations from the virtual spacecraft location indicated with the black dot in panel (a). The anticorrelation between magnetic field and density fluctuations is compatible with mirror mode waves. Adapted from Hoilijoki et al. (2017)

*Magnetopause Reconnection:* Global simulations allow us to study the interaction between the solar wind and the magnetosphere, including how and to which extent dayside magnetic reconnection is affected by the solar wind and magnetosheath dynamics. Hoilijoki et al. (2017) investigate this topic by using a 2D-3V Vlasiator global simulation in the GSE polar $xz$ plane, focusing in particular on the laminar or bursty nature of magnetic reconnection during steady solar wind conditions (Fig. 18).

The simulation domain covers $x = [-94, \ +48]\,R_{\mathrm{E}}$ and $z = [-56, \ +56]\,R_{\mathrm{E}}$ ($R_{\mathrm{E}} = 6371\, \mathrm{km}$ is the Earth’s radius) and it features a 2D line dipole centered at the origin modelling the Earth’s magnetosphere dipole and scaled to match the geomagnetic dipole strength. The steady solar wind has a density $n = 1\,\mathrm{cm}^{-3}$, a constant velocity $\mathbf{v}_{SW} = - 750\,\mathrm{km/s} \ \hat{\mathbf{x}}$ and a proton temperature of 0.5 MK. The interplanetary magnetic field (IMF) is directed purely southward and it has a magnitude of 5 nT. The resolution is uniform in the simulation domain; the spatial resolution is 300 km ($\sim 0.047 \,R_{\mathrm{E}} \sim 1.3 \ d_{p,SW}$, where $d_{p,SW}$ is the proton inertial length in the solar wind) and the velocity space resolution is 30 km/s ($\sim 0.33 \ v_{\mathrm{th,p,SW}}$, where $v_{\mathrm{th,p,SW}}$ is the solar wind proton thermal speed). The solar wind flows into the simulation domain from the boundary at $x = +48\,R_{\mathrm{E}}$ with constant parameters. The boundaries of the simulation box are periodic in the out-of-plane $y$ direction while the $-x$ and $\pm z$ boundaries apply copy boundary conditions. The inner boundary of the magnetosphere is a circle of radius $4.7\,R_{\mathrm{E}}$ centered at the origin and it enforces a static Maxwellian proton velocity distribution and perfect conductor field boundary conditions.

Hoilijoki et al. (2017) reported that, despite the steady solar wind conditions, magnetic reconnection at the subsolar magnetopause does not reach a steady state and it is very dynamic. Indeed, magnetic islands are constantly produced and the presence of multiple X-points is observed as is evident in Fig. 18b. The motion of the X-points appears to be mostly dictated by the outflow produced by the neighboring X-points. Hoilijoki et al. (2017) suggest that including the ion kinetic physics in the model promotes the development of a dynamic and bursty reconnection process at the dayside.

This study investigates also the reconnection rate at the multiple simultaneous X-points and how the rate is affected by the local plasma conditions near the X-point. In particular, the presence of mirror modes (Fig. 18c) in the magnetosheath appears to affect the reconnection rate, in agreement with spacecraft observations (Laitinen et al. 2010). The dependence of the magnetopause reconnection rate upon the IMF direction is further investigated in global Vlasiator simulation by Hoilijoki et al. (2019). In particular, the run presented in Hoilijoki et al. (2017) is compared to a run with similar parameters but with a positive component of the IMF ($B_{IMF} = [3.54, \ 0, \ -3.54] \ nT$). The Sun-ward tilt of the IMF results in a smaller tangential field at the magnetopause, leading to a reduction of the reconnection rate with respect to the purely southward-directed IMF case. The presence of a non-zero $B_{x,IMF}$ introduces an asymmetry that impacts the reconnection process in terms of flux transfer events (FTE) size, speed and occurrence rate. In particular, FTEs are observed more frequently in the Northern Hemisphere and they are smaller in size with respect to the Southern Hemisphere.

The findings of Hoilijoki et al. (2017) have been confirmed by Pfau-Kempf et al. (2020), who analyze the reconnection process in a three-dimensional setup reproducing the magnetopause surface. In particular, Pfau-Kempf et al. (2020) report a Vlasiator simulation of the noon–midnight meridional plane which is extended to cover 7 $R_{\mathrm{E}}$ in the dawn–dusk direction. The study by Pfau-Kempf et al. (2020) is the first example of a 3D-3V Vlasiator simulation of a cylindrical geometry mimicking the subsolar dayside magnetosphere. While the dimensionality is increased, the cylindrical geometry and the limited extent in the $y$ direction allow keeping the computational cost affordable and much lower than a global 3D-3V simulation modeling the entire magnetosphere.

The simulation domain covers $x = [-16, \ +31]\,R_{\mathrm{E}}$, $y = [-3.5, \ +3.5]\,R_{\mathrm{E}}$ and $z = [-35, \ +35]\,R_{\mathrm{E}}$ and the solar wind and IMF parameters are the same used for the 2D-3V run reported in Hoilijoki et al. (2017) and discussed above. The spatial resolution is $\sim 0.24\,R_{\mathrm{E}}$, which is larger than the resolution of 2D-3V Vlasiator simulations because of the increased computational cost of 3D-3V runs.

Identifying the magnetic reconnection site in 3D settings is not as straightforward as in 2D-3V simulations, where the local behaviour of the flux function allows to identify saddle points corresponding to X-points that are associated with reconnection sites. Hence, in this study the X-line location is estimated by combining the four-field junction method (Laitinen et al. 2006) with the identification of the locations exhibiting a flow reversal in the $z$ direction. However, the four-field junction method is insufficient for identifying multiple reconnection X-lines. Pfau-Kempf et al. (2020) find that, despite the uniform initial condition and the cylindrical symmetry in $y$, the X-line is not a straight line and it exhibits variations along the $y$ direction. It is suggested that structures in the magnetosheath break the translation symmetry along $y$. Analogously to Hoilijoki et al. (2017), Pfau-Kempf et al. (2020) point out that reconnection is bursty and patchy, with multiple reconnection sites being present at the various $z$ and $y$ locations across the magnetopause, despite the homogeneous and steady-state solar wind conditions.

*Magnetotail Reconnection:* Recently, magnetotail reconnection has been investigated in the context of a 3D-3V Vlasiator simulation investigating the dynamics of plasma eruptions (Palmroth et al. 2023). The three-dimensional simulation in both ordinary and velocity space is made possible by technological advances, notably by enabling static adaptive mesh refinement (AMR) for ordinary space (Ganse et al. 2023). With AMR, regions of high scientific interest such as the magnetotail plasma sheet are sampled with higher resolution (0.16 $R_{E}$) with respect to other regions in the simulations, the coarser resolution is 1.26 $R_{E}$.

The 3D-3V simulation domain covers $x = [-111, \ +50]\,R_{\mathrm{E}}$ and $y, z = [-58, \ +58]\,R_{\mathrm{E}}$. The simulation parameters and initial conditions (IMF, solar wind density and speed) are the same adopted in (Hoilijoki et al. 2017). However, since this is a 3D-3V run, the Earth’s dipole is 3D and the inner boundary is a sphere of radius 4.7 $R_{E}$, while the $\pm y$ boundaries apply copy boundary conditions, as the $\pm z$ boundaries. Differently from (Hoilijoki et al. 2017; Palmroth et al. 2018a; Juusola et al. 2018), where Ohm’s law included only the Hall term, this run includes the electron pressure gradient term as well. A polytropic closure is adopted for electrons, $\mathbf{P}_{e} = p_{e} \mathbf{I}$ and $p_{e} = n^{\gamma }T_{e}$, where $\mathbf{P}_{e}$ is the electron pressure tensor, $p_{e}$ is the scalar pressure, $T_{e}$ is the electron temperature and $n$ is the density. The polytropic index $\gamma $ is set to $5/3$ (adiabatic).

**Fig. 19:**
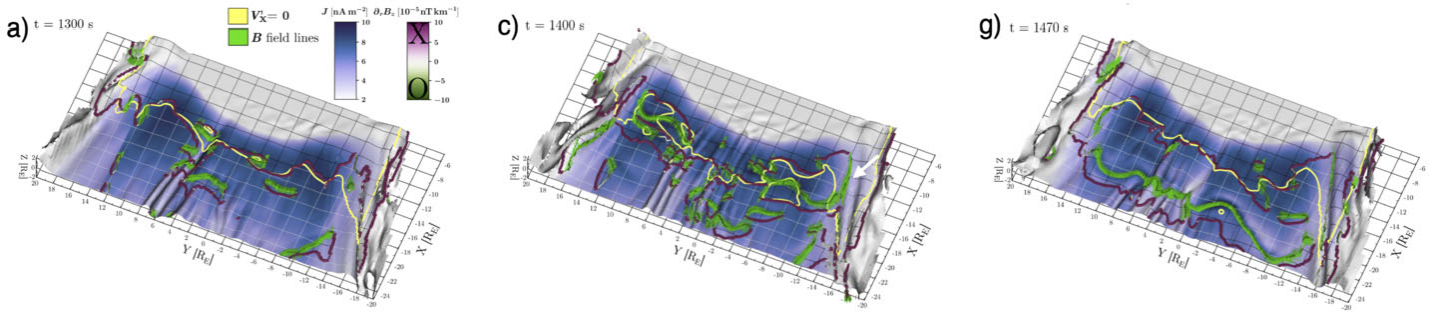
Evolution of the magnetotail current sheet in a 3D-3V Vlasiator simulation. The panels show the current sheet surface (defined as $B_{r} = 0$) at different times, $t = 1300 \ \text{s}$ (a), $t = 1400 \ \text{s}$ (c), $t = 1470 \ \text{s}$ (g). The color of the surface corresponds to the current density $J$. The yellow line indicates the flow reversal between the Earthward and tailward reconnection outflow. The magenta and green lines are locations where $B_{r} = 0$ and $B_{z} = 0$ and correspond to X-lines and O-lines (differentiated using the sign of $\partial B_{z}/\partial r$, which is positive at the X-lines and negative at the O-lines). The primary reconnection line is where the X-line (magenta) and flow reversal (yellow) contours are approximately co-located. The background grid shows the coordinates but also the magnetic-field topology: the black grid shows areas where the magnetic field is directed northward, and the white grid shows the areas where it is southward-directed. Adapted from Palmroth et al. (2023)

Palmroth et al. (2023) focus on the investigation of magnetotail, revealing complex dynamics where magnetic reconnection and kinking instability co-exist in the magnetotail current sheet. In particular, in Fig. 19 it is shown that both processes are required to induce a global topological reconfiguration of the magnetotail, with the formation of a tail-wide plasmoid which is released and rapidly moves tailward. As mentioned above, the identification of the reconnection sites is challenging in 3D systems since we cannot rely on the identification based on the flux function. The reconnection site (X-line) in the magnetotail is identified by a combination of magnetic field and velocity proxies. X-lines and O-lines are identified as the locations where both $B_{r} = B_{z} = 0$, where $B_{r}$ is the radial magnetic field component. The quantity $\partial B_{z}/\partial r$ allows us to distinguish between X-lines ($\partial B_{z}/\partial r>0$ in the magnetotail) and O-lines ($\partial B_{z}/\partial r<0$ in the magnetotail). The locations where an X-line is co-located with a $\mathrm{v_{x}}$ reversal (diverging plasma flow) are identified as reconnection sites. Palmroth et al. (2023) further confirm that reconnection is ongoing at those locations by showing reconnection signatures such as the Hall electric field and ion demagnetization. In the 3D-3V run, a dominant tail-wide reconnection X-line is found at $X \sim -15 \ R_{E}$ throughout the simulation. The dominant X-line is very dynamic and new X-lines and O-lines with limited extent in the Y-direction are constantly formed.

## The Rice Convection Model

### Introduction:

As a reminder, the equations in this section are written in SI units. The Rice Convection Model (RCM), by definition, never includes the physics of reconnection. However, reconnection is a microscale/mesoscale process. Only a small fraction of the magnetic flux in the magnetosphere is included in reconnection at any given time. Including the RCM allows us to discuss how the reconnection process impacts the rest of the magnetosphere, specifically the inner magnetosphere, where the magnetic field lines are closed. Since the RCM’s outer boundary condition comes from the plasmasheet, any process in the tail, such as reconnection, can impact the inner magnetosphere. Specifically, reconnection can generate low entropy bubbles that move toward the Earth at high speeds and can be a significant source of transport of plasma and magnetic field from the tail to the inner magnetosphere. In addition, the RCM helps quantitatively predict the plasmas from the inner magnetosphere which can reconnect on the dayside.

### Assumptions and Equations

The physics behind the RCM can be found in detail in Toffoletto et al. (2003) and Wolf (1983), and a detailed discussion of the use of the RCM can be found in Wolf et al. (2016) and Toffoletto et al. (2003), and ring current models are described by Toffoletto (2020). In the RCM, the distribution of magnetospheric particles is assumed to be isotropic, that is divided up into multiple energy channels. For each channel, a key variable is the isotropic energy invariant

77 $$ \left | \lambda _{k} \right | = W_{k}\left ( \mathbf{x}, t \right ) V^{2/3} $$

where $W_{k}$ is the particle kinetic energy, including bounce and gyro motion, $k$ is the energy channel label, and the sign of $\lambda _{k}$ is positive for positive ions and negative for electrons. The flux tube volume is

78 $$ V=\int ^{nh}_{sh} \frac{ds}{B(\mathbf{x},t)} $$

where the integral extends along the field line from the southern to the northern ionosphere.

The motions of magnetospheric particles in the inner magnetosphere are assumed to be governed by

$$ \text{Drift velocity} \ll \text{bounce motion} \ll \text{gyro motion} $$

The RCM calculates the bounce-averaged drift velocity, including gradient, curvature, and $E \times B$ drifts, i.e.,

79 $$ \mathbf{v}_{k}= \frac{\left (\mathbf{E} - \frac{1}{q_{k}}\nabla W_{k}\left (\mathbf{x}, t \right ) \right )\times \mathbf{B}(\mathbf{x},t)}{B(\mathbf{x},t)^{2}} $$

where $q_{k}$ is the charge of a particle of species k. Inertial drift is assumed to be negligible. The quantity $\eta _{k}(\mathbf{x},t)$ is defined as the number of particles per unit magnetic flux for particles of a specific chemical species and a specific value of energy invariant. It follows a conservation law (Wolf 1983)

80 $$ \bigg[ \frac{\partial}{\partial t} + \mathbf{v}_{k}\big(\lambda _{k}, \mathbf{x},t \big)\cdot \nabla \bigg]\eta _{k} = -L\big( \eta _{k} \big) $$

where $L$ is the loss rate of particles due to precipitation and charge exchange. The classic RCM neglects particles flowing up from the ionosphere to the magnetosphere. In the early 2000’s Stan Sazykin and Darren DeZeeuw implemented a grid-based scheme using the CLAWPAK package (De Zeeuw et al. 2004; Mandli et al. 2016) that was more robust but a bit more diffusive than the earlier Lagrangian scheme.

The flux tube content $\eta _{k}$ is related to the thermodynamic pressure $P$

81 $$ PV^{5/3} = \frac{2}{3} \sum _{k} \eta _{k} \lvert \lambda _{k} \rvert $$

while the flux tube content is related to the plasma distribution function $f_{k}(\lambda )$ as

82 $$ \eta _{k} = \frac{4\pi 2^{1/2}}{m^{3/2}_{k}} \int ^{\lambda _{max}}_{ \lambda _{min}}\lvert \lambda \rvert ^{1/2} f_{k}(\lambda ) d\lambda $$

where $(\lambda _{max}-\lambda _{min})$ is the width of the invariant energy channel associated with species $k$. The species $k$ is defined for a given chemical species (usually $\text{e}^{-}$, $\text{H}^{+}$, $\text{O}^{+}$), and the specific value of the energy invariant.

The electric field can be expressed as the sum of a potential component and an inductive component

83 $$ \mathbf{E} = -\nabla \Phi -\mathbf{v}_{induction}\times \mathbf{B} $$

In the RCM, the inductive electric field is included implicitly through time-dependent magnetic field mappings. The inductive magnetic field in the ionosphere is assumed to be zero there; however, it is not zero in the magnetosphere.

There are two more complications in the electric field:

1. In the classic RCM, we assume the electric field is perpendicular to the magnetic field.
2. There are two coordinate systems used in the classic RCM. One moves with the Earth as it rotates, and the potential in that system is labeled $\Phi _{i}$. The other does not rotate with the Earth, is approximately an inertial system, and is labeled $\Phi $.

We can translate from one system to the other in the ionosphere using the formula

84 $$ \Phi = \Phi _{i} - \frac{\omega _{E} B_{0} R^{3}_{E} \,\sin ^{2}(\theta _{i})}{R_{i}} $$

where $\omega _{E}$ is the angular rotation rate of the Earth, $B_{0}$ is the magnetic field at the Earth’s equator, $R_{E}$ is the radius of the Earth, $\theta _{i}$ is the colatitude, and $R_{i}$ is the radius of the ionosphere. Equation (84) applies to the equatorial plane, but it applies only to a dipole magnetic field. To compute $\Phi $ in the equatorial plane of the magnetosphere, the RCM calculates $\Phi $ by mapping between the ionosphere to the equatorial plane, assuming $\Phi $ is constant along each field line.

In the thin-shell approximation, the equation for the conservation of current is $(\nabla \cdot \mathbf{J} =0)$ can be written

85 $$ \nabla _{i} \cdot \Big[ \overleftrightarrow{\Sigma} \cdot \Big( \nabla _{i} \Phi _{i} \Big) \Big]= \Big( J_{\parallel nh} - J_{ \parallel sh}\Big) \;\sin (I) $$

where $\overleftrightarrow{\Sigma}$ is the field-line integrated conductivity tensor due to both hemispheres, $I$ is the dip angle of the magnetic field in the ionosphere, and $J_{\parallel nh} - J_{\parallel sh}$ is the ionospheric field-aligned current density.

The Vasyliunas (1970) equation, which is based on force balance

86 $$ \mathbf{J} \times \mathbf{B} - \nabla P = 0 $$

is given by

87 $$ \frac{J_{\parallel nh} - J_{\parallel sh}}{B_{i}}=\frac{\hat{b}}{B} \cdot \nabla V \times \nabla P $$

which relates field-aligned currents in the ionosphere to pressure gradients in the magnetosphere, and $B_{i}$ is the magnetic field at the southern- and northern-ionospheric footprints of the field line (assumed the same). The derivation makes use of the fact the right-hand side of Eq. (87) can be evaluated anywhere along the field line. The RCM equations are solved on a fixed ionospheric grid that has variable grid spacing in latitude to better resolve the auroral zone. The RCM grid is time-dependent in the equatorial plane, ranging from just inside the magnetopause on the dayside to $10-20 R_{E}$ on the night side.

### Inputs, Boundary, and Initial Conditions and Outputs

*The magnetic field model:* For many years, the RCM assumed a constant magnetic field, but, beginning about 2000 the RCM used a time-dependent semi-empirical model such as the Tsgyanenko models (1989, 1995, 2003). The RCM can also use the Hilmer and Voigt (1995) magnetic field model. In classic RCM runs, the magnetic field is not designed to be consistent with Ampere’s law and equation (86). Ways of including Eq. (86) consistently in the RCM are described in Sect. 9.4. In the ionosphere, where the magnetic field is assumed to be dipolar, the magnetic field is

88 $$ \mathbf{B}_{i} = -\frac{\mu _{0}}{4\pi} \frac{\mathbf{M}_{E}-3\hat{r}\big( \mathbf{M}_{E} \cdot \hat{r} \big)}{R^{3}_{i}} $$

where $\hat{r}$ is the radial unit vector in the ionosphere, and $\mathbf{M}_{E}$ is the magnetic moment, which is in the southern direction, and

89 $$ B_{i} = \hat{r} \cdot \mathbf{B}_{i} $$

*Ionospheric conductance:* Ionospheric conductance has two major drivers: Solar heating (e.g., the Sheffield University Ionosphere Plasmasphere (SUPIM) model, Bailey et al. 1997). The second is Auroral heating. The standard treatment uses the electron precipitating energy flux and average energy (Robinson et al. 1987).

*Loss models:* Separate models are needed for electrons and ions. The simplest electron loss model assumes a fixed fraction of strong pitch-angle scattering, often between $33\%$ and $67\%$ (Schumaker et al. 1989). That procedure is reasonable for the plasma sheet but overestimates the electron loss rate in the inner magnetosphere. A slightly more sophisticated model (Chen and Schulz 2001) is somewhat more realistic. For the Ion loss model, there are many theoretical models of ion charge exchange. The overall ion loss rate for an energy and $L$ value is calculated using an algorithm developed by James Bishop (Freeman et al. 1993; Bishop 1996).

The RCM needs boundary conditions both at its outer (large $L$) boundary and at its lowest (low $L$) boundary. The large$-L$ boundary depends on MLT as well as UT. Note that the large$-L$ boundary can’t be aligned with the grid, except in a few cases. At this boundary, the number $\eta _{s}$ which is the number of particles for a given type $s$ per weber of magnetic flux, is needed as well as the potential distribution, which can be a simple function of solar wind conditions or an empirical model such as the Weimer model (1985).

The low-L boundary (low-latitude) is set at least a few degrees latitude from the equator. Given the aligned-dipole assumption, the latitudinal current density should, in principle, be zero at the equator. The RCM and many other models use a thin-wire approximation to represent the region near the equator, to provide a boundary condition

90 $$ \frac{\partial J_{\theta}}{\partial \theta} + S \frac{\partial J_{\phi}}{\partial \phi} = 0 $$

which was derived by Blanc and Richmond (1980). Here $J$ is the current density, $\theta $ is the colatitude coordinate, and $\phi $ is the longitudinal coordinate. $S$ is a function of $\phi $ that was defined by Blanc and Richmond (1980).

*Initial conditions:* These are needed to provide the value of the initial value of $\eta _{k}$, which is a function of grid location and time. The RCM can be initialized with an empty value and run for a period of time to fill in the inner magnetosphere. Alternatively, the RCM uses the Spence et al. (1989) model for the initial pressure distribution. Earlier versions of the RCM assumed a Maxwellian distribution, but more recent versions have the option to assume a kappa distribution (e.g., Yang et al. 2015).

*RCM outputs:* The main RCM outputs are the electric potential $(\Phi )$, field-aligned currents $(J_{\parallel nh}-J_{\parallel sh})$, the distribution function $(\eta _{s})$ and moments (pressure and density) within the RCM modeling region in the ionosphere and the magnetospheric equatorial plane.

### Generalizations of the RCM

There have been many modifications to the RCM, particularly since 2000.

*Other planets:* Tom Hill and several of his students modified the RCM to be appropriate for Jupiter and Saturn. Jupiter’s moon Io has volcanoes that loft neutrals and positive ions into the inner magnetosphere, and there is also a similar effect at Saturn’s moon Enceladus. Centrifugal force is stronger than gravity near Io, and the region beyond Io is consequently interchange unstable. In the simulations, plasma develops finger-like structures, moving outward because of centrifugal force and azimuthally because of Coriolis force. The clearest magnetospheric signature of interchange transport occurs in the inner magnetosphere of Saturn, where the hot plasma injection-dispersion structures are evident (Hill et al. 2005 and references therein).

*CRCM (Comprehensive Ring Current Model):* This model (Fok et al. 2001) was similar to the classic RCM, except that it used a much more complete equation for the distribution function. Whereas the classic RCM assumed an isotropic pitch-angle distribution, CRCM assumes conservation of the first and second invariant. Additional terms account for precipitation and charge-exchange losses and pitch-angle scattering. CIMI (Comprehensive Inner Magnetosphere Model) includes radiation belt electrons and the plasmasphere (Fok et al. 2014).

*RCM-E:* In the classic RCM, the magnetic field was not required to satisfy the force balance equation $\mathbf{J}\times \mathbf{B} = \nabla P$ but $P=\big( 2/3 \big) V^{-\gamma} \sum _{k} \lvert \lambda _{k} \rvert \eta _{k} $ and $\eta _{k}$ was based on the theoretical equation (80), with $\gamma = 5/3$. The RCM-E (equilibrium) is run for a small time step (typically $1-5$ minutes), and a modified MHD code, called the “friction code”, recalculates the magnetic field in order to make it approximately consistent with $\mathbf{J}\times \mathbf{B} = \nabla P$ (Lemon et al. 2003). For conditions of strong convection, the time development of the RCM-E would cause the magnetic field to be tail-like and more like a substorm growth phase. If $PV^{\gamma}$ was constant on the nightside large $L$-boundary, it became difficult to form a realistic strong ring current (Lemon et al. 2004). The RCM-E usually exhibited the pressure balance inconsistency (Erickson and Wolf 1980). In other words, the more theoretically consistent model became a less realistic representation of observations. Other modelers have developed models that are variants of the RCM-E — e.g., Chen et al. (2012) or the RAM-SCB model (Zaharia et al. 2006) — that use an alternative ring current and force balance model. The solution to the pressure balance inconsistency turned out to be bursty bulk flows (BBFs), which are localized regions of the inner and middle plasma sheet (Angelopoulos et al. 1992) that flow rapidly earthward. The flow bursts, which are also often called “bubbles”, often move very fast (typically $400~\text{km}/\text{s}$). These flows correspond to regions of low $PV^{\gamma}$ (Pontius and Wolf 1990). Angelopoulos et al. (1992) found that BBFs account for a large fraction of the total earthward flow in the plasma sheet. BBFs usually terminate about the inner edge of the plasma sheet, although some of the fast flows penetrate the ring current (Gkioulidou et al. 2014; Yang et al. 2016). Lemon et al. (2004) produced a substantial ring current injection by reducing the $PV^{\gamma}$ at the RCM’s outer boundary over a limited region of local time, simulating a ring current injection during a storm. Yang et al. (2014a) showed a possible relation to the streamers observed in the polar cap and bubbles in the plasma sheet, and Yang et al. (2014b) argued that this effect could account for pressure balance inconsistency and that during storms low entropy flux tubes could account for up to $60\%$ of the ring current (Yang et al. 2015). See also the Sect. 9.5 below.

*RCM-I (RCM-Inertial):* The main problem with using RCM-E to represent BBFs is they move so fast that the assumption of force balance is not valid. Yang et al. (2019) developed a more complex version of the RCM that includes inertial effects in a very approximate way. Equation (85) is replaced by a much more complex expression that involves a $\partial \Phi / \partial t$ term.

### Large, Coupled Models That Include RCM

Single fluid, global MHD models have become powerful tools in recent years (e.g., Lyon et al. 2004; Raeder et al. 2001, Zhang et al. 2019). However, these models do not capture all the important physics in the inner magnetosphere where gradient and curvature drifts become important but are neglected in MHD. Coupling these models is a daunting task as the modeling regions overlap in space and information is fed back and forth between them. There are different physics assumptions associated with each model: the RCM model assumes slow flow and force balance and neglects waves, while MHD does not. However, MHD does not include energy-dependent drifts that are important in the inner magnetosphere. There have been several successful efforts to couple the RCM with global MHD, which provides many of the inputs used by the RCM (boundary and initial conditions) such as the magnetic field, plasma density, and pressure as well as the ionospheric potential. In return, the RCM provides the density and pressure that are derived from computing the moments from the RCM distribution function that have been subject to energy-dependent drifts.

*SMWF:* The earliest successful coupling effort was De Zeeuw et al. (2004) which merged the BATS-R-US Global MHD (Tóth et al. 2005) code with the RCM. The RCM was embedded in the Global MHD code as a subroutine that later became part of the Space Weather Modeling Framework (SWMF) (Tóth et al. 2005). Each coupling exchange requires computing many field-line integrals to obtain the flux tube volume (Eq. (78)), which is used by the RCM, and the mapping of the 2D RCM quantities into the 3D domain of the MHD code, which is then used to update the MHD. This field line tracing requires using a parallelized and efficient field line tracer that exploits the nested adaptive grid used in the MHD code (e.g., De Zeeuw et al. 2004. This version of the model demonstrated that including the RCM increased the pressure in the inner magnetosphere ring current region as compared to standalone MHD. The RCM also was able to model the Region-2 currents in the ionosphere. Later versions of the SWMF also include other inner magnetosphere models such as the Comprehensive Ring current model (CRCM) (Glocer et al. 2013) and other models (Tóth et al. 2012), becoming the first coupled magnetosphere model to be used in the NASA’s Community Coordinated Modeling Center (CCMC).

*LFM-RCM:* Pembroke et al. (2012) coupled the RCM to the Lyon Fedder Mobary (LFM) global MHD code (Lyon et al. 2004) that included the MIX ionosphere model (Merkin and Lyon 2010). This approach used a loose coupling scheme where the models (LFM, MIX, and RCM) ran independently as separate processes and used the InterComm software package to exchange information at pre-set intervals (Lee and Sussman 2004). In this model, RCM returned both pressure and density to the MHD code and included a simple static plasmasphere based on the Gallagher et al. (2000) empirical model. Since the RCM was only tracking the distribution function and not computing the potential as in the standalone RCM, the assumption of zero dipole tilt could be relaxed in the coupled model, allowing for more realistic simulations. The resulting coupled model was very dynamic, especially during geomagnetic storm simulations, and significantly impacted the ring current region (e.g., Wiltberger et al. 2017). To keep the code stable, the RCM boundary was restricted to regions where the field line average plasma beta was less than 1. With moderately strong solar wind driving, the coupled model produced a strong ring current and Region-2 currents.

*OpenGGCM-RCM:* Hu et al. (2010) and Cramer et al. (2017) coupled the OpenGGCM global MHD code to RCM (Raeder et al. 2001) that also includes the Coupled Thermosphere-Ionosphere Model (CTIM) (Fuller-Rowell et al. 1996). The RCM is embedded within the MHD code, where the feedback to the MHD code used a configurable ramp-up region based on the strength of the magnetic field for numerical stability. Cramer et al. (2017) found that most of the transport of plasma to the inner magnetosphere is via low entropy bubbles, consistent with Yang et al. (2015). Raeder et al. (2016) also used the coupled model to simulate a geomagnetic storm and showed that it developed subauroral polarization streams (SAPS) from electron precipitation computed from the MHD code. Hu et al. (2011) examined the entropy profile in an idealized Open GGCM simulation and found that violations of the frozen-in-flux in MHD could lead to an entropy profile that produced a low entropy bubble that was earthward of an entropy enhancement. Such a configuration causes the bubble/blob pair to move earthward/tailward, which thins the current sheet in the region between them and can ultimately result in tearing or other configuration changes.

*MAGE:* The newest version of a global magnetosphere model is the Multiscale Atmosphere Geospace Environment Model (MAGE) that couples the RCM to the Grid Agnostic MHD for Extended Research Application (GAMERA) global MHD code (Zhang et al. 2019; Sorathia et al. 2021), the ReMIX ionosphere model (Merkin and Lyon 2010) which is a revised version of the MIX solver, and the NCAR Thermosphere-Ionosphere-Electrodynamics General Circulation Model (TIE-GCM) (Roble et al. 1988). The GAMERA MHD model is derived from the LFM model but with improved numerical algorithms and updated software designed for efficient use on modern supercomputers. The coupling to the RCM has also been significantly improved and modernized, for example, it uses a highly configurable and customizable parallel field line tracer. Other improvements include moving the RCM boundary in MAGE further from the Earth compared to the coupled LFM-RCM code, allowing more plasma from the plasmasheet to move into the RCM modeling region, and the option of a Maxwellian distribution to compute RCM distribution functions replaced with a Kappa distribution (Sciola et al. 2023). The new model also includes improved loss rate mechanisms for electrons (Bao et al. 2023) where the electron precipitation model is based on RCM-computed electron energy fluxes that are used to modify the ionospheric conductances (Lin et al. 2022a). The use of this conductance model was found to influence the formation of the SAPS channel (Lin et al. 2021). The model also includes a dynamic plasmasphere density that is tracked using a zero-energy channel in the RCM that is fed back to the MHD model (Bao et al. 2023). Pham et al. (2022) used the coupled MAGE model to investigate the impact on thermospheric density perturbations produced by traveling ionospheric disturbances. Sciola et al. (2023) found that in the MAGE model, fast magnetospheric flows associated with low entropy channels can contribute over $50\%$ of the ring current population, consistent with Yang et al. (2015) and Cramer et al. (2017).

### Summary

While the RCM does not model reconnection, it is impacted by it. Reconnection in the tail produces low entropy flux tubes that rapidly interchange their way toward the Earth (e.g., Wiltberger et al. 2015; Sorathia et al. 2021). Some of these flux tubes make it into the inner magnetosphere and play an important role in the formation and structure of the ring current region. It can also have ionospheric effects such as the formation of streamers. Over the years, the RCM has helped illuminate the impact of processes in the tail on the inner magnetosphere. This is especially true using the new generation coupled models that have been developed in recent years. However, there are several limitations in the models that present challenges. One is the modeling of the region in the tail where the magnetic field is transitioning from a stretched tail-like configuration to a dipole. When fast flows appear in this region, as they often do, neither MHD, which neglects gradient/curvature drifts, nor the RCM, which assumes slow flow, are applicable.

Furthermore, including the RCM in MHD models can also affect the location and effectiveness of dayside reconnection as well. Since the Region-2 FACs modeled by the RCM shields the inner magnetosphere from convection, it also strongly affects the distribution of return flow back to the dayside boundary. The RCM has been successful in modeling the plasmaspheric plumes that can bring dense plasmas to the dayside reconnection regions (Goldstein et al. 2002; Huba and Sazykin 2014; Bao et al. 2023). Dayside SWMF runs that include RCM all successfully place MMS within 1 $R_{E}$ of at an X-line (or separatrix) whenever clear EDRs are observed (e.g. Reiff et al. 2017).

The next generation coupled models will need to add the effect of ionospheric plasma sources (e.g., Glocer et al. 2009a; Varney et al. 2016), which will ultimately require a multi-fluid MHD model coupled to a ring current model and includes a model of ionospheric outflow to track all the species in the magnetosphere.

## Conclusions

In this paper, we have presented a “brief” overview of the large collection of computational methods that are used to study magnetic reconnection. It should be clear to the reader of this text, that simulating magnetic reconnection is nearly a separate field of study in and of itself. It should be and has been (Büchner et al. 2003) the topic of entire books. The single element to take away from this paper is that simulating a multifaceted and multiscale problem like magnetic reconnection is not a simple endeavor. Both plasma models and simulation initial conditions must be tuned carefully to match the goals of the study.

We have reviewed simulation methods for magnetic reconnection in space plasmas, from macroscopic MHD scales to microscopic kinetic scales. Basically, macroscopic plasma behaviors can be simulated based on fluid modes, and as we resolve smaller scale physics, kinetic models need to be implemented in simulations. In addition, we have reviewed novel approaches to incorporate multi-scale physics.

MHD simulations are useful to study large scale physics, including performing global simulations for planetary magnetospheres. We have discussed the basic algorithm for MHD simulations, and also how to implement test particles that follow MHD fields. Hall MHD simulations contain Hall physics, which allows kinetic scale waves to propagate, mediating fast reconnection. We have reviewed recent progresses of Hall MHD studies, and also the effect of the electron inertia term and EMHD.

Hybrid PIC simulations and full PIC simulations include particle kinetic physics, where particle motions are directly solved by equations of motion of particles. For hybrid simulations, we have reviewed techniques to overcome the limitation of spatiotemporal resolution in global models, and also the implementation of electron kinetic physics into hybrid simulations. For full PIC simulations, we have reviewed simulation studies of magnetic reconnection in the magnetotail and magnetopause, particle acceleration, and shock driven reconnection.

Next, we have reviewed two novel approaches to address multi-scale physics in magnetic reconnection: embedded PIC, and kglobal. In the embedded PIC approach, the macro-scale region is solved using MHD equations, and local kinetic domains are embedded in the MHD domain, where full PIC techniques are employed. In kglobal, on the other hand, equations for the ion and electron fluids are combined with the particle equations for electrons, and the macro-scale evolution is modified by the kinetic physics.

Finally, we have reviewed two types of other simulation techniques: Vlasov simulations and the Rice convection model. In Vlasov simulations, kinetic effects are implemented in simulations by solving 2D-3V or 3D-3V Vlasov equations. We have discussed recent progresses of studies by Vlasov models from local reconnection simulations to global simulations. The Rice convection model is a kinetic approach to simulate physics of the inner magnetosphere, where bounce averaged particle drift motion is taken into account.

### Outlook

We first wish to stress that even relatively simple numerical models continue to shed light on the essential physics of magnetic reconnection, even though some of these models have been around for decades. A good example of this is the explanation of fast magnetic reconnection in collisionless plasmas–the longstanding Reconnection Rate Problem. Only in the last few years, built upon the endeavors of previous theoretical efforts, has a convincing theory that passed the cross-examination of all these numerical models (i.e., PIC, hybrid, Hall-MHD, EMHD, resistive-MHD, pair plasmas) been developed to offer the rate prediction (Liu et al. 2022). Comparing and contrasting “numerical experiments” of reconnection in these models provides invaluable, rigorous constraints to a theory. Such constraints from different models will continue to play a pivotal role in the theory development of any nonlinear phenomenon in plasmas, especially when one attempts to discern the “cause” from the “consequences”.

A major driver on the progress of simulations of reconnection has been the uncanny steady exponential increase in computing power known as Moore’s law (Schaller 1997). If technology manages to continue Moore’s law, current simulations will undoubtedly continue to yield major insights. For example, fully kinetic particle in cell simulations are only just now reaching around 100 ion inertial scale sizes in three dimensions. However, such massive simulations require extremely large data storage, with significant environmental implications.

Beyond increasing computational power, more efficient algorithms continue to be a source of study. For example, GPUs have allowed a significant increase in computational speed for modest sized systems (Bard and Dorelli 2014). For global hybrid models, novel algorithms that drive particles based on “events” rather than time steps are another new direction. The HYPERS global hybrid simulations (Omelchenko and Karimabadi 2012) described in Sect. 4 is one such example.

A major thrust in the coming decade is expected to be the feedback between the multiple disparate scales associated with reconnection. One such multi-scale example is the connection between microscales and mesoscales during reconnection at the dayside of Earth’s magnetosphere, where the formation of x-lines depends strongly on magnetic geometry. Coupling different models, as described in Sect. 6, is a promising way forward to address these questions, but there are significant challenges to overcome, especially in regards to boundary conditions and disparate length and time scales.

Another example of such multi-scale physics is reconnection particle energization throughout the heliosphere. A major challenge in the effort to understand the energization of electrons and ions has been the inability to explore the kinetic dynamics of particles in very large systems. Because particle-in-cell (PIC) and hybrid models have to resolve kinetic scales, which are a small fraction of typical macroscales (a factor of around $10^{-10}$ in the case of solar flares). The consequence is that although PIC simulations have been successful in identifying some of the dominant acceleration mechanisms of electrons and ions (Drake et al. 2006; Dahlin et al. 2014; Guo et al. 2014; Li et al. 2019a), their success in producing the extended powerlaws seen in flare and magnetotail observations has been limited. The largest PIC simulations have revealed electron powerlaws that extend only a single decade in energy (Li et al. 2019a) while observations reveal that powerlaws in flares extend across many decades in energy (Lin et al. 2003; Vilmer 2012). The production of powerlaw distributions of ions has been an even greater challenge (Zhang et al. 2021, 2024). However, PIC modeling revealed that Fermi reflection during the growth and merger of large-scale flux ropes dominates particle acceleration (Dahlin et al. 2016; Guo et al. 2014; Li et al. 2019a), a result that led to a new computational model, kglobal, that combined MHD fluid and particle descriptions while eliminating the kinetic scales that constrained the macro-scale modeling of flares (Drake et al. 2019; Arnold et al. 2019). A major accomplishment was the first exploration of magnetic reconnection-driven electron acceleration in macro-systems which produced electron powerlaw distributions extending nearly three decades in energy and revealed that the ambient guide field is the dominant control factor of the powerlaw index (Arnold et al. 2021). The success of the kglobal model motivates two major extensions: to include particle ions so that the partitioning of energy between the two species can be explored; and to incorporate the kglobal particle algorithm into a global simulation code such as the Adaptively Refined MHD Solver (ARMS) (DeVore 1991) flare simulation code or the Block Adaptive Tree Solar-wind Roe Upwind Scheme (BATS-R-US) (Gombosi et al. 2002). The resulting model would be able to simulate macroscale energy release during magnetic reconnection with energetic electrons and produce synthetic photon spectra for comparison with remote observations of solar flares observations.

## Footnotes

1 The CFL condition dictates that an element of the distribution function $f$ may not cross more than one velocity space grid spacing in one time step under the acceleration due to the Lorentz force.
